# Rational Molecular Design for Boosting Afterglow Efficiency in Nonplanar Carbazolocarbazoles

**DOI:** 10.1021/jacsau.4c01002

**Published:** 2025-01-23

**Authors:** Po-Cheng Liu, Jian Lei, Cheng-Chan Liu, Yu-Tzu Fan, Tien-Lin Wu

**Affiliations:** † Department of Chemistry, National Tsing Hua University, No. 101, Sec. 2, Kuang-Fu Rd., Hsinchu 300044, Taiwan; ‡ Institute of Atomic and Molecular Sciences, Academia Sinica, No. 1, Sec. 4, Roosevelt Rd., Taipei 106319, Taiwan; § College of Semiconductor Research, National Tsing Hua University, No. 101, Sec. 2, Kuang-Fu Rd., Hsinchu 300044, Taiwan

**Keywords:** carbazolocarbazole, room-temperature phosphorescence, afterglow, spin−orbital coupling, photoluminescence quantum yield

## Abstract

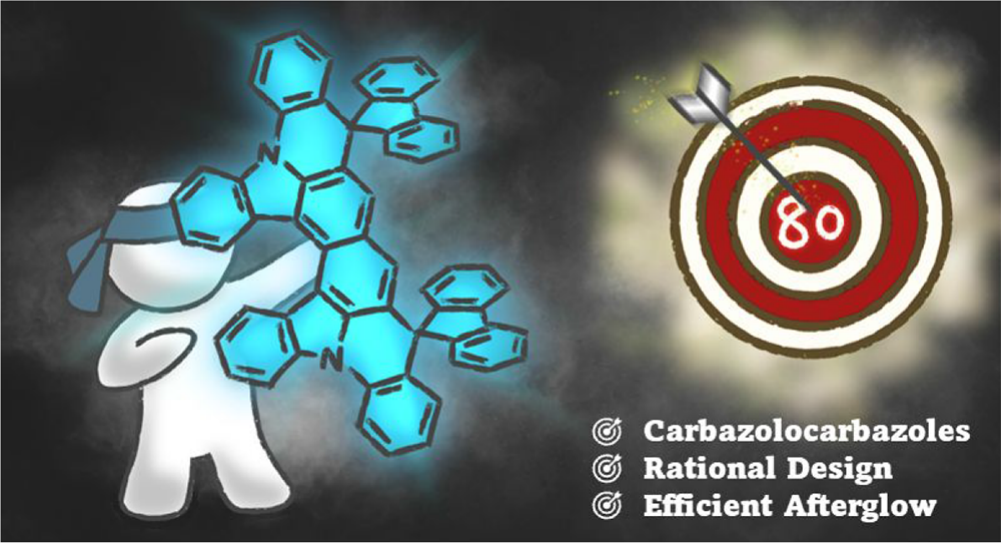

Carbazolocarbazole (CCz) represents a novel class of heterocycles in the carbazole family, distinguished by its unique structure and related properties. However, the significant challenges associated with its synthesis have constrained the exploration and related studies in recent years. Herein, we presented a thorough and reproducible method for synthesizing gram-sized helical CCz, extending its functionalization to investigate both the pristine CCz and its six derivatives with various substituents. This work is the debut of CCz compounds displaying afterglow characteristics in the solid state. The CCz derivatives demonstrated impressive ultralong room-temperature phosphorescence (URTP) lifetimes, ranging from 0.52 to 0.72 s, which are considered relatively long among the carbazole family. The URTP of the CCz series occurs due to the efficient intersystem crossing (ISC) process from the singlet to the triplet-excited states, facilitated by the enhanced spin–orbit coupling (SOC). Based on the comprehensive investigation of seven CCz compounds, we proposed a strategy for elevating the photoluminescence quantum yield (PLQY) of organic URTP emitters. Notably, the most rigid CCz derivatives, SpiroCCz, exhibited the highest total PLQY of 79.9%, one of the highest carbazole-based afterglow emitters in a polymer matrix. Overall, helical CCz derivatives are innovative and promising candidates for afterglow applications in optoelectronic devices, bioimaging, and security printing.

## Introduction

**Figure 1:**
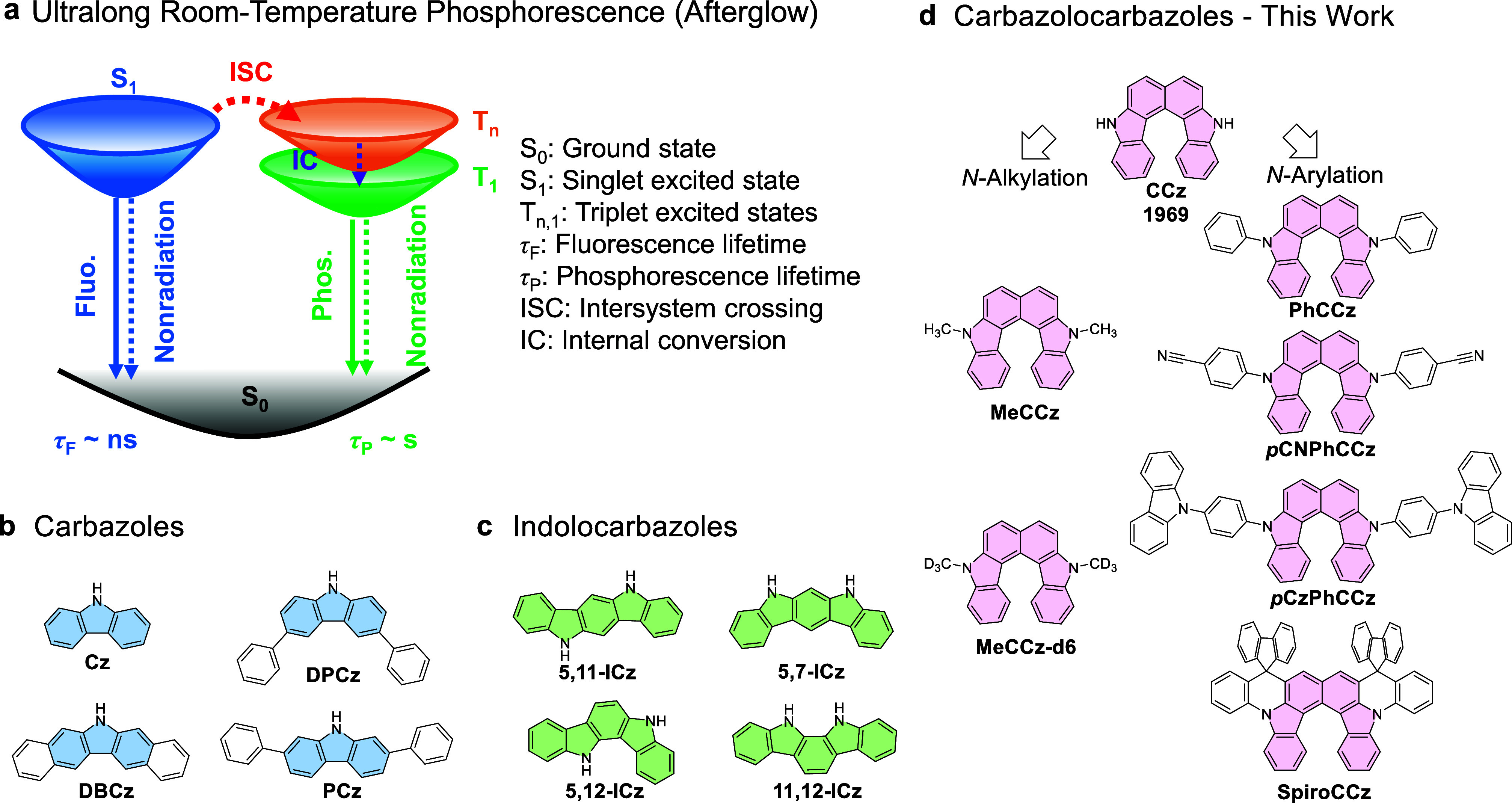
(a) Simplified Jablonski diagram depicting the source of ultralong room-temperature phosphorescence. (b) Carbazole and its derivatives with an afterglow. (c) Indolocarbazole isomers with an afterglow. (d) Carbazolocarbazoles presented in this work.

Afterglow luminescence is a phenomenon in which some materials emit light for a long-lasting time after being excited, and the emission lifetime of afterglow generally exceeds 100 ms.^1,2^ The duration time can vary from a few seconds to several hours, influenced by the matrix selection or the excitation condition. Consequently, afterglow emitters have attracted significant attention for their potential applications in bioimaging,^3,4^ sensing,^5^ security,^6,7^ and optoelectronics.^8,9^ Ultralong room-temperature phosphorescence (URTP)^10−12^ is a term used to describe the phenomenon of afterglow in organic materials. The general strategy for achieving RTP is to introduce a noble metal^13,14^ or heavy atom^15−18^ to enhance spin–orbital coupling (SOC) between singlet and triplet-excited states.^19^ However, RTP or even URTP from purely organic fluorophores is rarely observed. Figure 1a reveals that achieving long-lived and highly efficient URTP requires two conditions: (i) a fast ISC process of S_1_ to T_*n*_ (*n* ≥ 1) and (ii) reduced radiative and nonradiative decay rates of T_1_ to S_0_.^20,21^ Crafting pure organic molecules to achieve this remains difficult due to the inherent properties of excited-state dynamics, such as a low intersystem crossing (ISC) rate, weak SOC, spin-forbidden triplet states, and significant nonradiative decay.^22,23^ In general, they suffer from the issues of slow radiative triplet decay rates (*k*_RTP_ < 1 s^–1^), slow *k*_ISC_ of S_1_ to T_*n*_, and fast nonradiative decay rates (*k*_nr,T_) of T_1_ to S_0_. Therefore, the main challenges in developing pure organic materials with URTP are (i) enhancing the ISC process of S_1_ to T_*n*_, which dominates the quantum efficiency ratio from the triplet state, and (ii) suppressing nonradiative decay of T_1_ to S_0_, which determines the RTP lifetime and quantum efficiency.

Carbazole is a heterocyclic and aromatic compound that consists of two benzene rings fused with a pyrrole ring. It is widely used as a building block for organic semiconductors, especially light-emitting materials.^24^ Carbazole derivatives have been extensively investigated for their photophysical and electrochemical properties, such as fluorescence, phosphorescence, electroluminescence, and charge transport.^25^ Specifically, carbazole derivatives, as illustrated in Figure 1, are the most renowned among organic afterglow systems. According to El-Sayed’s rule,^26^ lone pairs on carbazole’s nitrogen are vital for enhancing SOC and facilitating the ISC process. Moreover, commercial carbazole has recently become one of the triggers for persistent luminescence.^27^ Prof. Liu’s group separated the isomer of carbazole (1*H*-benzo[*f*]indole) from several commercial carbazoles and revealed that low-concentration impurities could stimulate the ultralong phosphorescence in their carbazole derivatives. Furthermore, the inheritance concept of slightly doping 6*H*-dibenzo-[*b*,*h*]carbazole (DBCz) in carbazole can produce a white afterglow with a high PLQY.^28^ Such low-doping or impurity-caused ultralong luminescence is also observed and studied in diphenylamine-based compounds.^5^ Besides, organic afterglow can be induced by matrix and substituent engineering. In Figure 1b, two examples of diphenylcarbazoles, DPCz^29^ and PCz,^30^ exhibit URTP in a rigid polymer or small-molecule host. Their afterglow durations exceed 7 s, highlighting their potential use in coatings and anticounterfeiting applications. Besides, carbazole derivatives with an extended indole structure, known as indolocarbazoles, also exhibit obvious afterglow properties in crystals or polymers. Figure 1c depicts various indolocarbazole isomers displaying URTP in polymer films, such as PVA and PMMA.^31,32^ By adjusting their doping ratios and temperatures, it is possible to achieve tunable afterglow in terms of both color and lifetime. The fields in question have reached a high level of development, offering a wealth of research and various derivative compounds. Some carbazole and indolocarbazole derivatives are even commercially available. However, these derivatives with URTP typically suffer from low photoluminescence quantum yield (PLQY) issues. Therefore, investigating new frameworks and tactics becomes essential to overcome this challenge.

Carbazolocarzole (CCz) is recognized as an infrequent member of the carbazole family, mainly due to its challenging synthesis.^33^ The initial documentation and synthesis of the helical isomer of CCz (Figure 1d) can be traced back to the publication by Zander *et al.* in 1969.^34^ Later on, the nonplanar conformation of helical CCz was revealed by XRD single-crystal analysis, and its helicity and chirality were further examined by Pischel *et al.*(35) Subsequently, only Icli *et al.* delved into its photophysical properties, detailing observations on steady-state fluorescence and emission quenching phenomena.^36^ After 2000, only one paper reported the functionalization of helical CCz for use in organic solar cell devices.^37^ In addition, prior research on nonplanar hydrocarbons like homotruxene observed RTP in the solid state,^38,39^ highlighting the significance of molecular configuration. The nonplanar geometry of aromatics enhances SOC^9,40^ and facilitates the ISC and RISC processes between singlet and triplet-excited states.^41−43^ The abovementioned literature indicates that research on helical CCz is currently scarce but holds considerable promise, sparking our interest in further examining its potential in photonics.

In this work, we investigated the rare and secret carbazole derivative, helical CCz, and discovered that its afterglow originated from RTP for the first time. Seven CCz compounds were designed and prepared for further exploration to solve the low PLQY issue among recent afterglow emitters, as shown in Figure 1d. The highly twisted conformations and nitrogen atoms with long pairs in the CCz series, confirmed by single-crystal XRD analysis, might enhance SOC strength and accelerate the ISC process. Based on a comprehensive experimental and theoretical study, we presented an approach for achieving high PLQY through modifications of functional groups. Theoretical studies align with experimental results and suggest that a rigid configuration could boost PLQY in afterglow materials. As a result, the long-lived and highly emissive SpiroCCz exhibited an afterglow lifetime of 0.72 s and a PLQY of up to ∼80%. Further demonstrations of SpiroCCz for data security highlight its potential due to its high PLQY attributes. Our novel discovery of CCz and proposed strategy could significantly impact organic RTP research.

## Results and Discussion

**Scheme 1:**
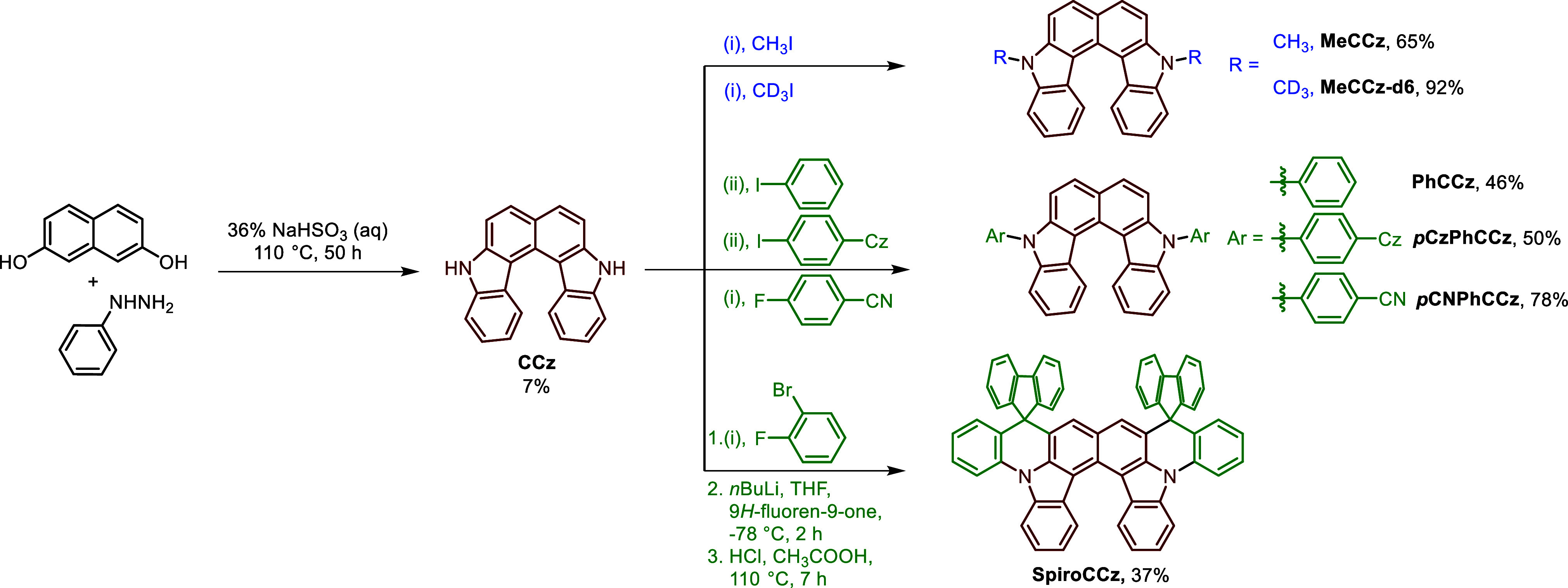
Synthetic Routes of all CCz Derivatives Conditions: (i) Cs_2_CO_3_, DMF, 150 °C, 24 h. (ii) Pd(OAc)_2_, [(*t*-Bu)_3_PH]BF_4_, NaO*t*Bu, 1,4-dioxane, 105 °C, 24 h.

Scheme 1 presents the complete synthetic plan of CCz and its derivatives. First, we stirred and refluxed two starting materials, 2,7-dihydroxy-naphthalene and phenylhydrazine, in an aqueous sodium bisulfite solution for 50 h to afford the pristine CCz. To functionalize the CCz, the first step of nucleophilic aromatic substitution (S_N_Ar) and Buchwald–Hartwig coupling were applied to achieve two *N*-alkylated (MeCCz and MeCCz-d6) and three *N*-arylated (PhCCz, *p*CzPhCCz, and *p*CNPhCCz) targets, respectively. SpiroCCz, the final product, utilized an initial S_N_Ar reaction, followed by two steps of lithiation and electrophilic intramolecular aromatic substitution, resulting in the most rigid and π-extended framework among all CCz compounds. Moreover, a high-performance liquid chromatography (HPLC) method monitors impurity levels and ensures product purity (Figures S1–S9), while ruling out any impact from impurity-induced afterglow.^27^ Particularly, for carbazole (Figure S5), we synthesized it in the laboratory instead of purchasing it commercially. The Supporting Information contains details of all synthetic procedures and characterizations of ^1^H/^13^C NMR spectra, HPLC chromatograms, high-resolution mass spectrometry data, and elemental analysis results. In addition, the thermal physical properties of all compounds were measured via thermal gravimetric analysis (TGA) and differential scanning calorimetry (DSC), as shown in Figure S10. All CCz derivatives show high thermal stability, with decomposition temperature (*T*_d_, 5% weight-loss) higher than 300 °C, as summarized in Table S1. PhCCz is the only compound exhibiting characteristics of glass transition, with its glass transition temperature (*T*_g_) measured at 236 °C—a relatively high value among organic small molecules.

**Figure 2:**
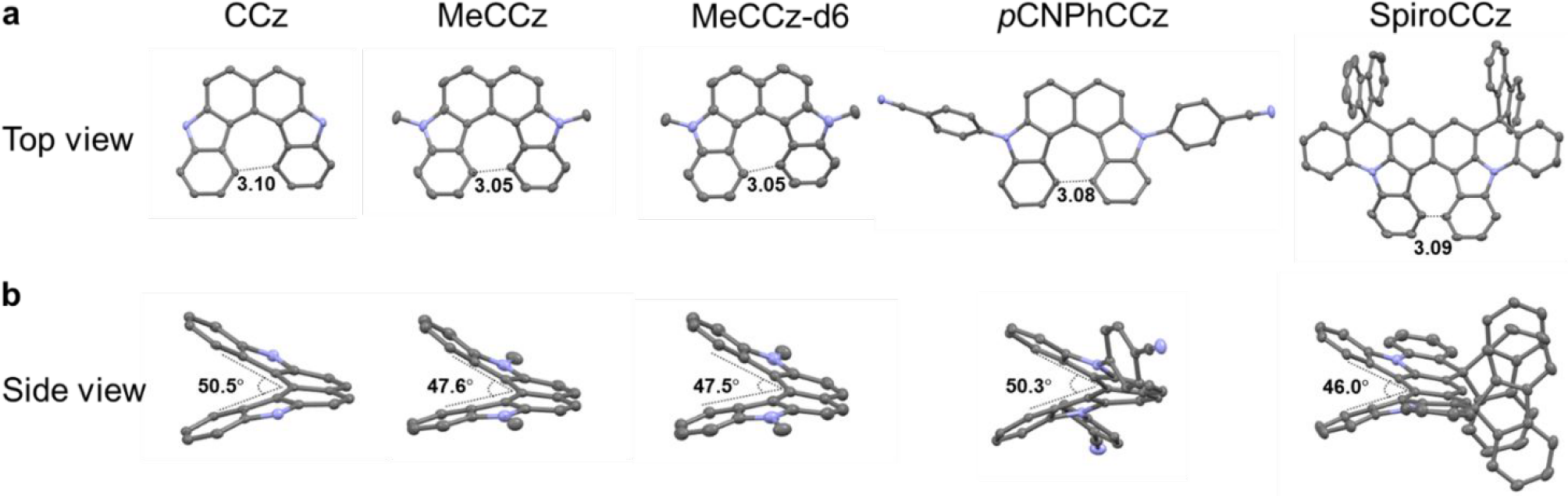
Single-crystal structures of five CCz compounds with thermal ellipsoids set at 50% probability. (a) Top view of five CCz compounds with selected distances given in Å. (b) Side view of five CCz compounds with corresponding dihedral angles. Hydrogen atoms (in both top and side views) are omitted for clarity.

To investigate the helicity of CCz compounds, five single crystals of CCz, MeCCz, MeCCz-d6, *p*CNPhCCz, and SpiroCCz were obtained and subsequently analyzed via X-ray diffraction (XRD), as shown in Figure 2. The dihedral angles of the five helical CCz structures range from approximately 45° to 50°, which are much smaller than those of [6]helicene and its analogs (50–59°).^44−46^ In helical CCz, the embedded five-membered rings constrain the distance between outer rings, leading to a more rigid helicene architecture. Their distances of the closest carbon atom are approximately 3.10 Å (Figure 2a), much shorter than the 4.14 Å observed in [6]helicene. Furthermore, the functionalized CCz derivatives show smaller angles (Figure 2b) than those of pristine CCz, implying that the substituents might further compress the helical structure. For *N*-alkylated derivatives of MeCCz and MeCCz-d6, the dihedral angles have nearly identical values (47.5°) and are both lower than the pristine CCz (50.5°). Moreover, the *N*-arylated *p*CNPhCCz exhibited a slightly decreased angle of 50.3°. Notably, SpiroCCz had the smallest angle value of 46.0°, which could lead to the most rigid conformation in this study. All single-crystal parameters are summarized in Table S2, and their ORTEP plots are displayed in Figure S11.

**Figure 3:**
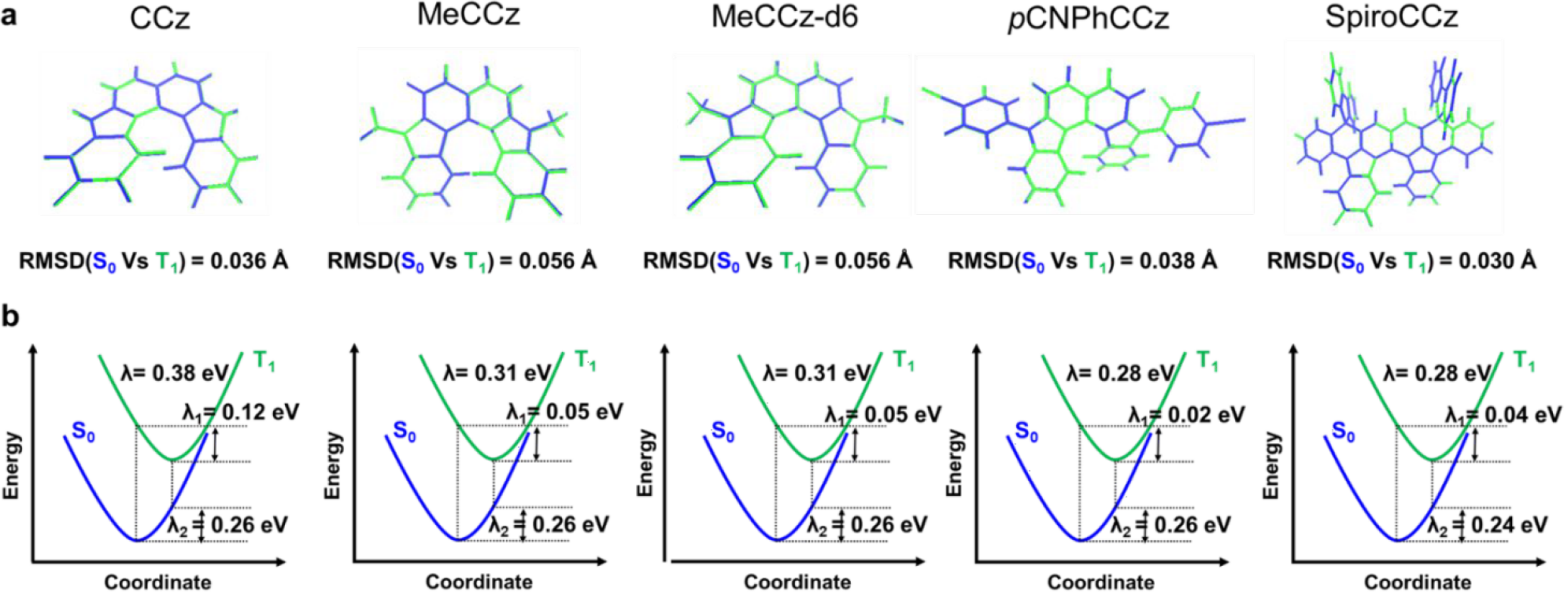
(a) Calculated RMSD between optimized ground (S_0,_ blue) and excited (T_1_, green) states of five CCz compounds in the single crystal at the B3LYP/6-31G(d) level. (b) Calculated reorganization energies (λ) with optimized ground (S_0,_ blue) and excited (T_1_, green) geometries of five CCz compounds in the solid phase at the B3LYP/6-31G(d) level.

**Figure 4:**
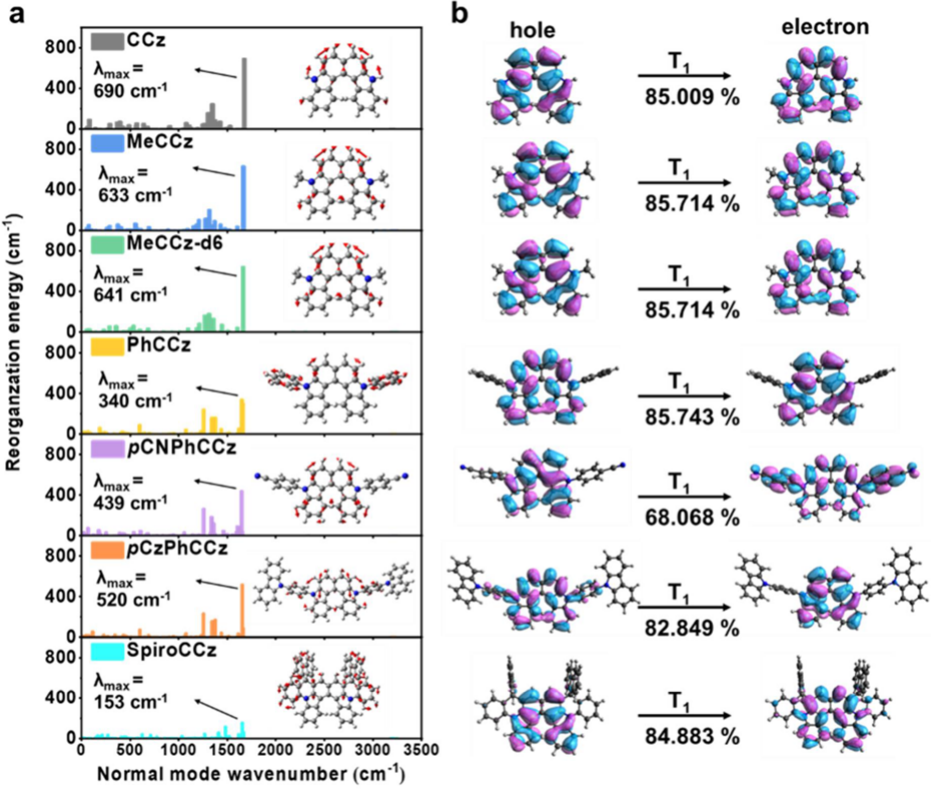
(a) Normal mode reorganization energy between S_0_ and T_1_ in the solid phase. (b) Natural transition orbitals (NTOs) for T_1_ based on the optimized S_0_ geometries in toluene. All calculations are at the B3LYP/6-31G(d) level.

To gain insights into molecular design, we carried out theoretical calculations to investigate the RTP behaviors of CCz derivatives under the polarizable continuum model (PCM)^47^ in toluene and the ONIOM method^48^ based on the QM/MM model in the solid phase (Figure S12, Tables S3 and S4). Furthermore, we calculated the root-mean-square deviation (RMSD) between S_0_ and T_1_ geometries in the solid phase to characterize structural deformations. In Figure 3a, the RMSD value of 0.030 Å for SpiroCCz is lower than those of CCz (0.036 Å), MeCCz (0.056 Å), MeCCz-d6 (0.056 Å), and *p*CNPhCCz (0.038 Å). The total reorganization energy (λ) between S_0_ and T_1_ of the five compounds is provided in Figure 3b. Both substituted CCz (*N*-alkylation and *N*-arylation) series could reduce the energy level compared to pristine CCz of 0.38 eV. *N*-arylated compounds, *p*CNPhCCz and SpiroCCz, exhibited lower values of 0.28 eV. Moreover, we depicted the reorganization energy from T_1_ to S_0_ for each normal mode in Figure 4a. The major contribution to C–H and C–C stretching modes in most compounds stems from the CCz skeleton. It is worth noting that SpiroCCz exhibits the lowest maximum reorganization energy contribution (λ_max_ = 153 cm^–1^) as the stretching vibration of the CCz core is transferred to the spirofluorene units. These results indicate that introducing spirofluorene units can inhibit T_1_ geometrical changes of molecules, potentially reducing the nonradiative decay rate of T_1_ and thereby improving RTP’s lifetime and quantum yield. Furthermore, the RMSD of 0.022 Å and reorganization energy of 0.17 eV for SpiroCCz were also the lowest among others in the S_1_ to S_0_ process (Figures S13 and S14).

Next, we analyzed the characters of the excited states using the natural transition orbitals (NTOs) for all molecules, as shown in Figure 4b. The transitions from hole to electron NTOs of T_1_ are dominant, with proportions larger than 82% for all molecules except for *p*CNPhCCz (∼68%). This result suggests an n−π*/π–π* mixture and intramolecular charge transfer (ICT) characteristics for *p*CNPhCCz. For S_1,_ T_1_, T_2_, and T_3_ states, most holes and electrons also extend to the CCz core due to their π-conjugated electron systems (Figure S15). More results of excited-state transition properties are listed in Tables S5 and S6. In addition, HOMO and LUMO levels of all molecules were calculated, as illustrated in Figure S16. Their electron distributions of HOMO and LUMO are mainly localized on the CCz cores. The LUMO levels of *p*CNPhCCz, *p*CzPhCCz, and SpiroCCz are relatively lower than those of CCz, MeCCz, MeCCz-d6, and PhCCz molecules. The results suggest that the introduction of electron-donating or electron-withdrawing groups on the CCz core could significantly alter the transition properties of molecules.

As previously mentioned, fast ISC of S_1_ to T_*n*_ and slow radiative processes of T_1_ to S_0_ are crucial to approach RTP’s high efficiency and long-lived lifetimes. According to Marcus eq (eq 1):^49,50^
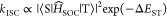
1

The ISC rate is influenced by the SOC and the energy difference (Δ*E*_ST_) between singlet (S) and triplet (T) states. In Figure S17, all CCz compounds displayed large SOCs on the transitions (S_1_ to T_2_ and T_3_) with lower energy gaps, resulting in effective ISC pathways from S_1_ to T_2_ and S_1_ to T_3_. Compared to pristine CCz, the oscillator strength (*f*) of SpiroCCz for the S_1_ state sharply enhanced from 0.0926 to 0.4052 owing to the conjugated structure and electron localization in SpiroCCz. For the T_1_ to S_0_ process, the oscillator strengths (*f*) of CCz, MeCCz, PhCCz, *p*CNPhCCz, *p*CzPhCCz, and SpiroCCz were 1.49 × 10^–9^, 1.36 × 10^–8^, 1.65 × 10^–9^, 2.08 × 10^–8^, 1.18 × 10^–8^, and 1.20 × 10^–9^, respectively. The radiative rate of T_1_ to S_0_ (*k*_r,T_) can be estimated using Einstein’s spontaneous *k*_r,T_ = *E*_T_*f*/1.499 s^–1^, suggesting the lowest *k*_r,T_ of SpiroCCz. In addition, RTP lifetime is determined by the radiative and nonradiative (*k*_nr,T_) decay rates (eq 2):^49^
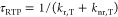
2

Given the theoretical findings, SpiroCCz will likely achieve the highest PLQY and longest RTP lifetime among CCz derivatives owing to its nonplanar and rigid framework.

**Figure 5:**
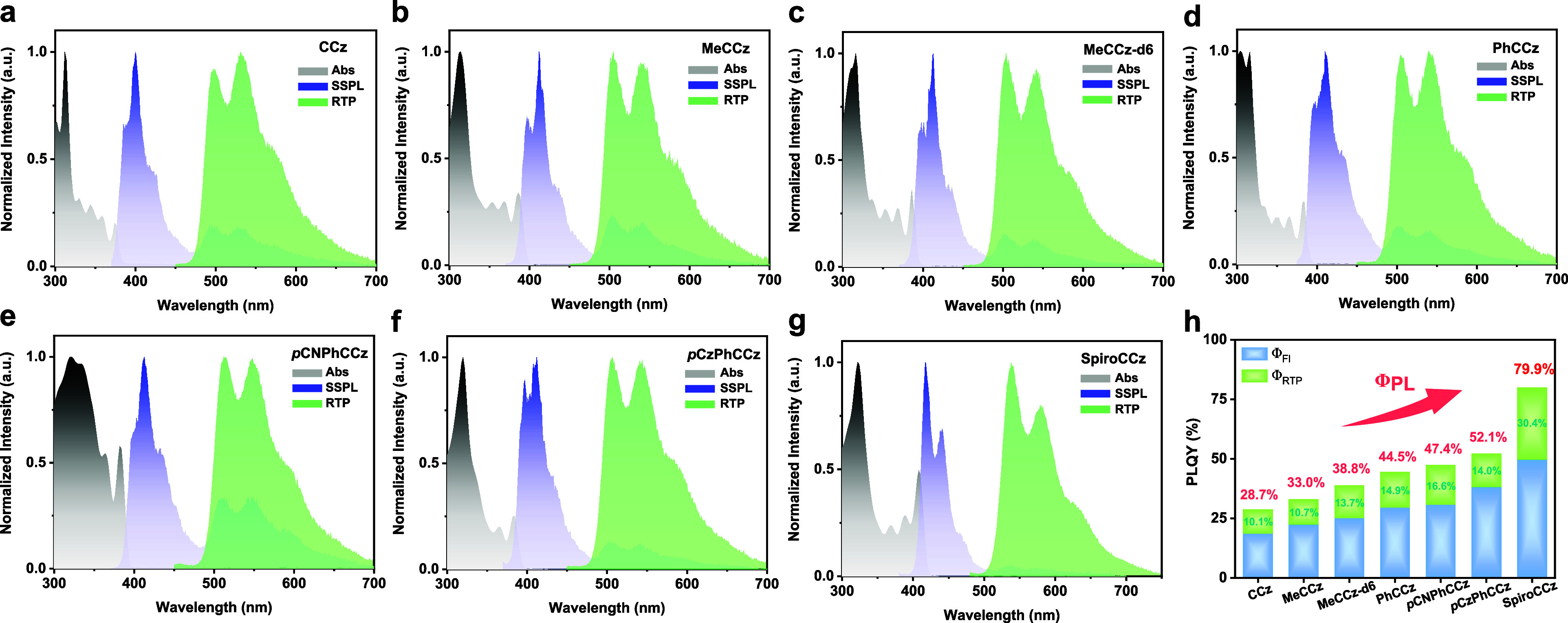
(a–g) Absorption (Abs) of CCz’s toluene solutions (10^–5^ M) at room temperature; steady-state PL (SSPL) and time-gated PL (RTP) spectra of the doped films (2 wt % CCz compounds in PMMA) at room temperature. (h) Φ_PL_ and Φ_RTP_ values of the doped films (2 wt % CCz compounds in PMMA) at room temperature.

Figure 5a–g shows the absorption and photoluminescence spectra of all of the CCz compounds. Seven emitters in toluene (10^–5^ M) exhibited major absorption bands around 312–322 nm, generally assigned as the π → π* transitions. Apart from *p*CNPhCCz, their absorption patterns are comparable, suggesting that major electron transitions occur on the CCz core. Figure S18 shows the steady-state PL spectra of CCz compounds in solutions (10^–5^ M), demonstrating fluorescence with two prominent peaks; their initial emission peak shifts from 382 to 419 nm, attributed to the CCz modifications with extended π-conjugation. Additionally, most CCz compounds exhibit locally excited (LE) properties, as verified by their emission spectra in various solvents. Only *p*CNPhCCz with electron-withdraw CN groups displayed partial ICT due to an extra emission band (Figure S18f) in dichloromethane, consistent with DFT predictions. Nevertheless, the afterglow is not detectable for all compounds in solutions. On the other hand, the steady-state PL spectra of 2 wt % CCz emitters doped in PMMA films show two band regions (∼400 and ∼500 nm) under vacuum or nitrogen; one is recognized as fluorescence (Fl), while the other is likely related to RTP. Due to substituent changes, all compounds’ PL bands in the Fl region (∼400 nm) shifted gradually from 400 to 419 nm (Table 1). Compared to their PL spectra in solution (Figure S18a), most solid-state Fl emissions shift to longer wavelengths and change patterns, with the second peak becoming dominant, indicating the effect of solid-state aggregation. Among all CCz compounds, SpiroCCz displays the same Fl peak values (419 nm) and similar patterns under both solution and film conditions, suggesting a lower tendency to aggregate. The additional spirofluorene units in SpiroCCz introduce significant steric hindrance and a rigid scaffold, further decreasing the degree of molecular packing and inhibiting structural vibrations. The lower intensity components observed in the second region (∼500 nm) could only be measured without oxygen, indicating that its origin is possibly from phosphorescence. To confirm the hypothesis, time-gated spectra with a 6 ms delay at room temperature (Figure 5a–g) were measured for all compounds, supporting their RTP characteristics. Most CCz compounds exhibit comparable RTP patterns with peaks near 500 nm, whereas SpiroCCz demonstrates an RTP peak shifted to 537 nm because of its extended conjugation area, lowering the triplet energy level.

To comprehensively understand CCz emitters, the absolute quantum yield measurement method was employed to determine the PLQY values of CCz compounds within an integrating sphere. We measured the doped PMMA films (2 wt % CCz emitters) under nitrogen to determine their total PLQYs (Φ_PL_s), which significantly rose from pristine CCz (28.7%) to SpiroCCz (79.9%), as shown in Figure 5h. Additionally, oxygen gas is used to quench the triplet exciton for determining the values of RTP’s QY (Φ_RTP_). In the *N*-alkylation series, MeCCz has a slightly increased Φ_PL_ of 33.0% compared to CCz, but their Φ_RTP_s remain nearly identical. The deuterium methyl groups in MeCCz-d6 suppress the nonradiative pathway,^50^ leading to a higher Φ_RTP_ of 13.7%. Furthermore, the *N*-arylation series has significantly enhanced both the Φ_PL_s and Φ_RTP_s to an impressive degree. PhCCz exhibited Φ_PL_ (44.5%) and Φ_RTP_ (14.9%) higher values than those of the *N*-alkylation series. The additional modifications to the phenyl ring with a donor (*p*CzPhCCz) or an acceptor (*p*CNPhCCz) could further increase their Φ_PL_s to 47.4 and 52.1%, respectively. Remarkably, SpiroCCz achieves the highest Φ_PL_ of 79.9% and Φ_RTP_ of 30.4%, which is relatively high compared to reported organic afterglow emitters. (Figure S19) The stiff and contorted structure of SpiroCCz effectively inhibits nonradiative pathways and aggregation-caused quenching (ACQ), leading to an exceptionally high PLQY. These findings strongly support our proposed strategy via theoretical predictions.

**Figure 6:**
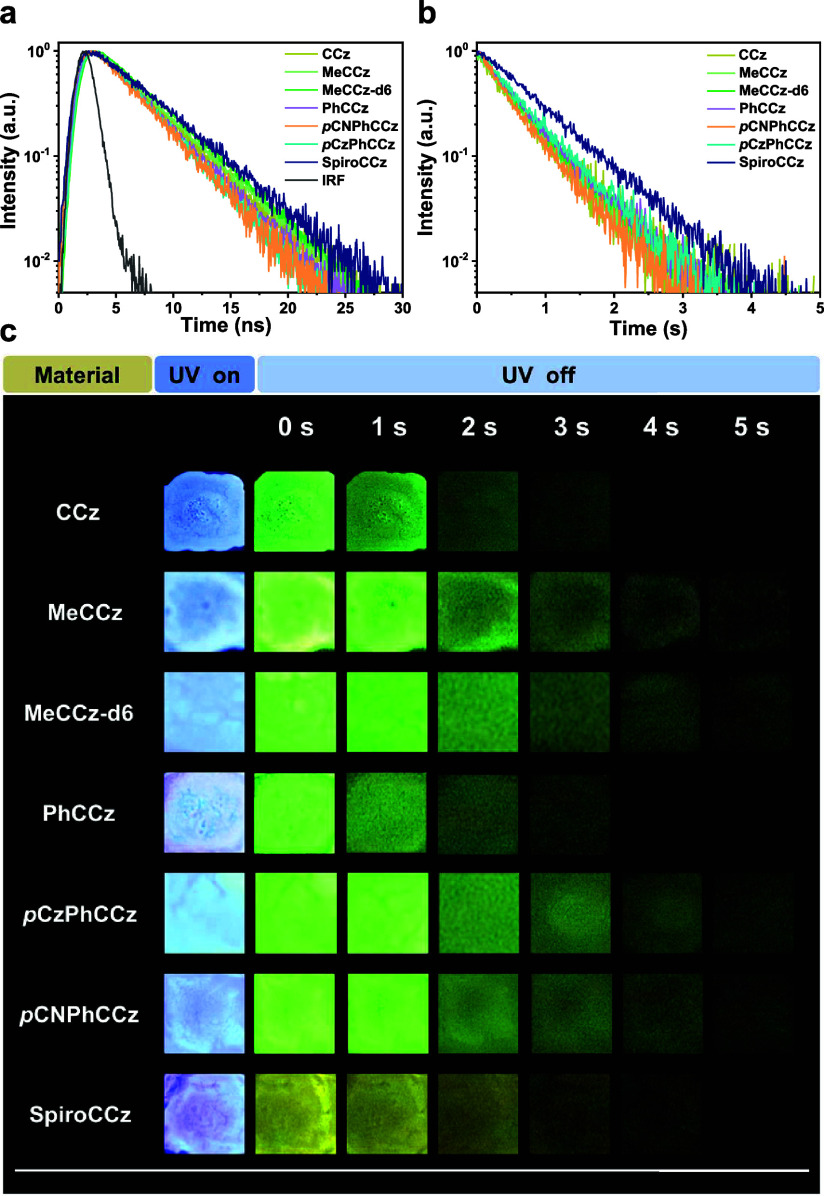
(a) Fluorescence decay curves of the doped films. (b) Afterglow decay curves of the doped films. (c) Camera-recorded images of the doped films displaying RTP afterglows under nitrogen with a 365 nm UV lamp. Doped films: 2 wt % CCz compounds in PMMA.

We then measured the doped films (2 wt % CCz compounds in PMMA) using a time-resolved spectrometer and a camera to investigate the excited state dynamics. Their transient PL spectra and photographs are displayed in Figure 6. Based on the above steady-state PL results, two regions (∼400 and ∼500 nm) are selected for the time-resolved PL measurements of each CCz emitter. Figure 6a shows the decay curves of Fl bands obtained through the time-correlated single-photon counting (TCSPC) method, and their fitted lifetimes are all around 4 ns, which reflects the general behavior of prompt fluorescence. For the RTP bands at around 500 nm, the decay profiles of seven CCz compounds display ultralong lifetimes ranging from 456 to 719 ms, proving afterglow characteristics at room temperature. (Figure 6b) Moreover, their afterglow was visible to the naked eye, and a camera recorded their duration. As shown in Figure 6c, all CCz compounds persist in emitting light for up to 5 s after the 365 nm UV lamp is turned off. We measured temperature-dependent transient PL at RTP regions to further confirm the afterglow’s origin. As the temperature decreases from 300 K, all emitters’ lifetimes gradually increase, which reveals the general behavior of phosphorescence. (Figure S20 and Table S7) The UV–vis absorption and fluorescence spectra, PLQYs, and corresponding rate constants of all CCz compounds were recorded and are summarized in Table 1. By combining measured PLQYs, calculated emission lifetimes, and theoretical simulations, we could further elaborate and analyze the excited-state dynamics for all emitters. While unsubstituted CCz exhibits enhanced SOC emitting URTP, it has the lowest PLQY (28.7%) and *k*_ISC_ (2.53 × 10^7^ s^–1^) of the entire series. MeCCz with methyl groups enhances the PLQY by marginally suppressing the vibration modes; its RTP component does not increase expectedly and results in an even slower ISC process (*k*_ISC_: 2.49 × 10^7^ s^–1^). Using deuterium methyl groups in MeCCz-d6 further constrains the structure stretching, increases the Φ_RTP_ to 13.7%, and elevates *k*_ISC_ to 3.23 × 10^7^ s^–1^. As expected, the introduction of aryl groups provides even greater improvements in decreasing *k*_nr_ and boosting *k*_ISC_. PhCCz, *p*CNPhCCz, and *p*CzPhCCz showed shorter prompt lifetimes (τ_Fl_ < 4 ns) and higher PLQYs (44.5, 47.4, and 52.1%) compared to those of CCz and alky-substituted CCz. As a result, the *N*-arylation approach successfully increases *k*_ISC_ up to 4.77 × 10^7^ s^–1^ and reduces *k*_nr_ below 1.15 s^–1^. With *N*-arylation and a rigid configuration, the well-designed SpiroCCz achieves outstanding performance among all CCz emitters. Based on simulations, the rigid structure of SpiroCCz significantly suppressed nonradiative channels arising from structural relaxations and stretching modes. More importantly, SpiroCCz contains the highest PLQY at 79.9% and a significantly elevated Φ_RTP_ of 30.4%. Meanwhile, SpiroCCz’s τ_RTP_ extends to the longest lifetime of 719 ms, with its visibility lasting up to 5 s to the naked eye, as shown in Figure 6c. As expected, the *k*_nr_ has been limited to only 0.27 s^–1^, and *k*_ISC_ has increased to the highest value of 6.47 × 10^7^ s^–1^. A decreasing trend of Δ*E*_ST_ in the helical CCz series correlates with an increase in *k*_ISC_, consistent with eq 1. In short, the excited-state dynamics and PLQY trends closely align with the theoretical results, validating our proposed approach for realizing an emissive afterglow molecule, SpiroCCz.

**Table 1:** Summary of the Photophysical Properties of the CCz Compounds

compound	λ_abs,max_^a^ (nm)	λ_SSPL,max_^b^ (nm)	λ_RTP_^b^ (nm)	Δ*E*_*ST*_^c^ (eV)	Φ_PL_^d^ (%)	Φ_RTP_^e^ (%)	τ_Fl_^f^ (ns)	τ_RTP_^f^ (ms)	*k*_RTP_^g^ (s^–1^)	*k*_nr_^g^ (s^–1^)	*k*_ISC_^g^ (10^7^ s^–1^)
CCz	312	400	497, 531	0.73	28.7	10.1	4.0	518	0.19	1.38	2.53
MeCCz	314	412	505, 543	0.68	33.0	10.7	4.3	530	0.20	1.26	2.49
MeCCz-d6	316	412	504, 542	0.68	38.8	13.7	4.2	534	0.26	1.15	3.23
PhCCz	316	411	505, 541	0.67	44.5	14.9	3.8	549	0.27	1.01	3.92
*p*CNPhCCz	322	413	513, 548	0.68	47.4	16.7	3.5	456	0.36	1.15	4.77
*p*CzPhCCz	320	409	507, 544	0.68	52.1	14.0	3.5	590	0.24	0.81	4.03
SpiroCCz	322	419	537, 578	0.64	79.9	30.4	4.7	719	0.42	0.27	6.47

a Absorption maximum in toluene (10^–5^ M).

b Steady-state PL maximum and RTP maximum bands.

c Δ*E*_ST_ = *E*_S_ – *E*_T_.

d Absolute total PLQY value.

e RTP component of PLQY.

f Lifetimes of the prompt fluorescence (τ_Fl_) and RTP (τ_RTP_).

g Rate constants of RTP, nonradiative decay, and ISC.

**Figure 7:**
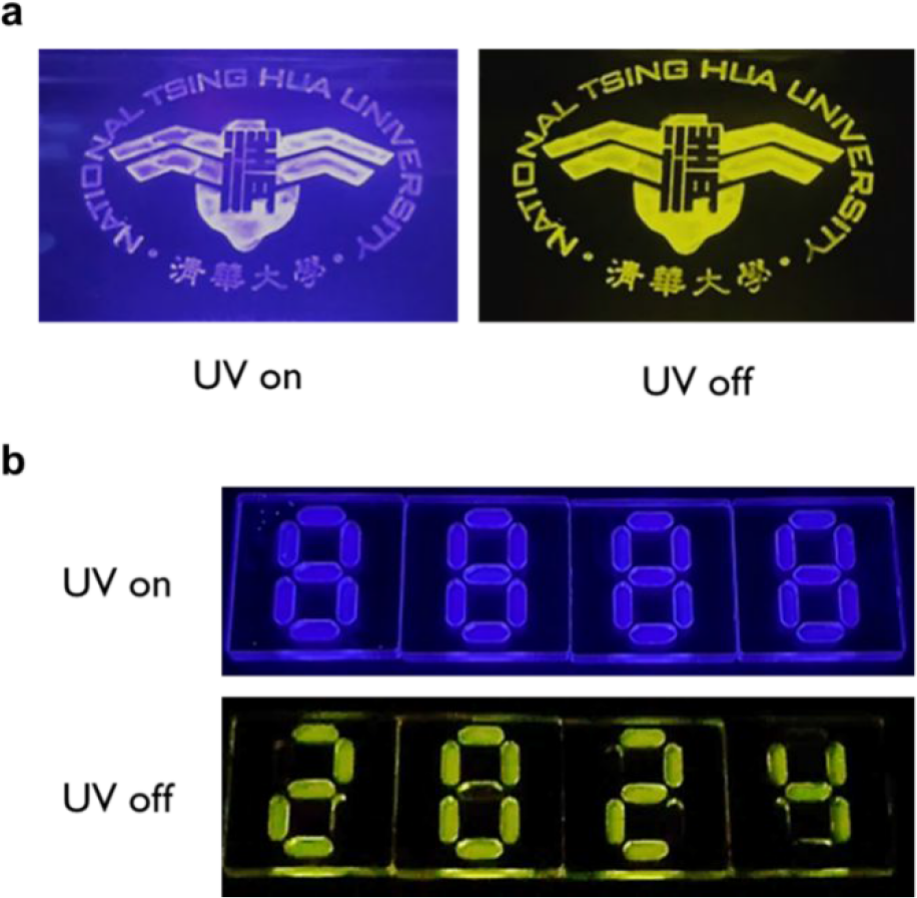
(a) Images of an anticounterfeiting application with the NTHU logo shown before (UV on) and after (UV off) turning off the 365 nm UV lamp. (b) Photographs of the information encryption before (UV on) and after (UV off) removal of the 365 nm UV lamp.

To further demonstrate the afterglow application using SpiroCCz, we initially varied its doping ratio in PMMA films and examined the related effects. Dopant concentrations of 0.1, 1, 2, and 10 wt % in PMMA films and a neat SpiroCCz film were employed for PL, transient PL, and PLQY measurements. Figure S21 illustrates the outcomes for the various doping ratios, as summarized in Table S8. At doping concentrations ranging from 0.1 to 2 wt %, the emission patterns and afterglow lifetimes (around 0.7 s) are similar (Figure S21c), suggesting minimal impact from aggregation with ratios below 2 wt %. The PLQY trends indicate that the 2 wt % doped film still exhibits the highest values, accompanied by a rise in RTP components (from 5.4 to 30.4%). However, with the doping ratio reaching 10 wt %, the PL spectrum shows an enhanced intensity of the second fluorescence band, while the URTP lifetime decreases to 0.6 s, and the PLQY drops to 50.8%. Moreover, SpiroCCz's neat film exhibited the lowest PLQY of 5.6% without a detectable afterglow. These results indicate that increasing the dopant concentration beyond 10 wt % would significantly suffer from the ACQ issue, adversely affecting the URTP emission. Therefore, we choose a 2 wt % doping ratio as the optimized condition for subsequent afterglow applications under a nitrogen atmosphere. Data security applications, including anticounterfeiting and encryption, were chosen to demonstrate using an ink derived from a SpiroCCz-doped-PMMA solution. Figure 7a displays an anticounterfeiting example using our university logo. The design pattern is invisible to the naked eye on paper, and it glows blue under a 365 nm UV lamp. Once the UV source is turned off, a high-contrast yellow afterglow appears and lasts about 5 s, illustrating its anticounterfeiting feature. (Movie S1) Furthermore, we presented the method of encrypted information by labeling the digital numbers “8888” using two formulas: one with 1 wt % SpiroCCz and 1 wt % fluorescent 9, 10-diphenylanthracene in a PMMA, and another with just 2 wt % 9, 10-diphenylanthracene in PMMA. When exposed to 365 nm UV light, the blue-illuminated “8888” becomes visible to the naked eye. After the UV source is turned off, the fluorescent area promptly vanishes, and then the hidden yellow message “2024” appears, as shown in Figure 7b.

## Conclusions

In summary, we have explored and functionalized the rare helical CCz species. This research represents the first discovery of the afterglow phenomenon in helical CCz and its six derivatives. The nonplanar geometry and nitrogen’s lone pair simultaneously enhance the SOC strength and ISC rate. At the same time, the PMMA matrix creates a confined environment that suppresses nonradiative pathways from triplet-excited to ground states. As a result, all compounds emit URTP in PMMA films, with lifetimes reaching 719 ms and visible durations lasting up to 5 s. More importantly, both experimental and theoretical findings have validated the PLQY enhancement of afterglow emitters through CCz functionalization (via N-alkylation and N-arylation). The well-designed and rigid SpiroCCz achieved the highest Φ_PL_ of 79.9% and Φ_RTP_ of 30.4%, with a reduced ACQ effect due to its highly twisted configuration in the solid state. This study uncovers the hidden aspects of the CCz family and offers a blueprint for developing organic afterglow systems. Such innovative design strategies for efficient URTP could open doors to various potential applications.

## Methods

### General Synthetic Procedures

All chemicals and reagents were purchased from commercial suppliers and used without further purification. Air-sensitive reactions were conducted under a nitrogen atmosphere using Schlenk techniques, and no special precautions were taken to prevent exposure to air or moisture during the workup. Column chromatography was carried out using silica gel (Geduran, Si 60, 63–200 μm). Analytical thin-layer chromatography (TLC) was performed with silica plates with aluminum backings (200 μm with an F-254 indicator). TLC visualization was accomplished with a 254/365 nm UV lamp. High-performance liquid chromatography was carried out on an Agilent 1260 Infinity II Quaternary System with a 4 μm Poroshell 120 EC-C18 column.

### Structural Characterization and X-ray Crystallography

^1^H NMR and ^13^C NMR spectra were determined on a Bruker AV-400 NMR spectrometer in CDCl_3,_*d*_*6*_-acetone, or *d*_*6*_-DMSO, and all NMR data were processed in TopSpin 3.6.5. The abbreviations have been used for multiplicity assignments: “s” for singlet, “d” for doublet, “t” for triplet, “q” for quartet, and “m” for multiplet. ^1^H and ^13^C NMR spectra were referenced to the residual solvent peaks with respect to TMS (δ = 0 ppm). Mass spectra were performed on the JEOL Model: JMS-T200GC AccuTOF GCx at the NYCU Instrumentation Resource Center. Elemental analyses were performed using an analyzer (Elementar vario EL CUBE CHN-OS Rapid, German) at the NCHU Instrumentation Center. Single crystals of CCz, MeCCz, MeCCz-d6, *p*CNPhCCz, and SpiroCCz were prepared via a slow slovent evaporation process or thermal gradient sublimation. X-ray diffraction was carried out on an X-ray diffractometer (Rigaku XtaLAB Synergy R, DW system, HyPix-Arc 150) at NTHU Instrumentation Center.

### Photophysical Measurements

Dilute solution samples were prepared in HPLC-grade solvents. UV–vis absorption spectra were recorded on a Hitachi U-3300 spectrophotometer, and fluorescence spectra in solution were recorded on a Hitachi F-7000 fluorescence spectrophotometer with a 1 cm quartz cuvette. The absolute photoluminescence quantum yields (PLQYs) were determined using an integrating sphere system (LQ-100X-PL, Enlitech) under a nitrogen atmosphere. Thermogravimetric analysis (TGA) and differential scanning calorimetry (DSC) were performed on a thermal analyzer (2-HT, Mettler-Toledo) at a heating rate of 10 °C/min from 30 to 800 °C under nitrogen. Steady-state and time-gated PL spectra and transient PL decay measurements were obtained using a time-resolved PL instrument. (FLS 980, Edinburgh) Photographs and videos were recorded by Canon EOS M50 or iPhone 12.

## Supporting Information Available

Additional data: synthetic details, materials characterization, and theoretical calculations (PDF)
Anticounterfeiting demo video (MP4)

## Accession Codes

X-ray Single-Crystals’ CIFs deposited at CCDC 2388623, 2388629–2388632 for CCz, MeCCz, MeCCz-d6, *p*CNPhCCz, and SpiroCCz, respectively.
