# Supplementary material for: Rational Molecular Design for Boosting Afterglow Efficiency in Nonplanar Carbazolocarbazoles
Source: JACS Au. 2025 Jan 23;5(2):756–65. doi: 10.1021/jacsau.4c01002 (PMC11863156; doi:10.1021/jacsau.4c01002)
Supplement: Supplementary file 1 — au4c01002_si_001.pdf [file au4c01002_si_001.pdf]

Supporting Information

Rational Molecular Design for Boosting Afterglow Efficiency in Nonplanar Carbazolocarbazoles

Po-Cheng Liu,[a,‡] Jian Lei,[a,b,‡] Cheng-Chan Liu,[a] Yu-Tzu Fan,[a] and Tien-Lin Wu[a,c,*]

E-mail: tlwu@mx.nthu.edu.tw

[a] Department of Chemistry, National Tsing Hua University No. 101, Sec. 2, Kuang-Fu Rd., Hsinchu 300044, Taiwan.

[b] Institute of Atomic and Molecular Sciences, Academia Sinica, No. 1, Sec. 4, Roosevelt Rd., Taipei 106319, Taiwan.

[c] College of Semiconductor Research, National Tsing Hua University No. 101, Sec. 2, Kuang-Fu Rd., Hsinchu 300044, Taiwan.

Table of Content:

I. Synthetic Procedure, Characterization, and Purity……S1

II. Thermal Properties……S10

III. Single-Crystal Analysis……S11

IV. Theoretical Calculations……S13

V. Photophysical Properties……S25

VI. Appendix……S29

VII. References……S56

I. Synthetic Procedure, Characterization, and Purity

Synthesis of 5,10-dihydrocarbazolo[3,4-c]carbazole (CCz)

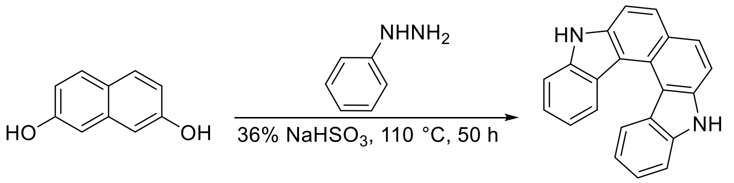

2,7-dihydroxy-naphthalene (50.00 g, 0.31 mol) in a solution of 36% $NaHSO_3$ (400 mL) and phenylhydrazine (70.4 g, 0.65 mol) were placed in a two-necked round-bottomed flask covered with aluminum foil and fitted with a reflux condenser and a mechanical stirrer. The reaction was heated to 110 °C under nitrogen atmosphere. For every 10 hours, an additional portion of phenylhydrazine (33.5 g, 0.31 mol) was added, and the reaction mixture was stirred at 110°C for another 10 hours. This procedure was repeated four times, totaling 50 hours of reaction time. The mixture was cooled down to room temperature and filtered through Celite with diethyl ether as eluent. The filtrate was collected and then concentrated under reduced pressure to remove some of the solvent. The residue was extracted with 50 ℃ of 2% KOH solution and diethyl ether for three times under appropriate precautions. The combined organic extracts were concentrated and transferred to a round-bottomed flask. The residue was further treated with 10% HCl solution and heated under reflux for 10 minutes. The mixture was cooled down and extracted with diethyl ether three times. The combined organic layer was dried over anhydrous $MgSO_4$ and filtered. The filtrate was concentrated, and the resultant residue was added small amount of dichloromethane. The brown suspension was allowed to stand in refrigerator overnight to precipitate the product. The product was isolated by filtration and thorough washing with methanol/dichloromethane affording the product (6.74 g, 7%) as a yellowish-white solid. ^{1}H NMR (400 MHz, DMSO, δH): 11.87 (s, 2H), 8.01 (d, J = 8.6 Hz, 2H), 7.72-7.65 (m, 6H), 7.40 (td, J = 1.0, 7.6 Hz, 2H), 7.12 (td, J = 0.9, 7.5 Hz, 2H); ^{13}C NMR (100 MHz, DMSO, δC): 139.9, 139.2, 127.9, 125.2, 123.6, 123.5, 123.4, 117.2, 113.9, 110.9, 109.6, 105.5; HRMS (FD) m/z: [M+] calcd. for $C_{22}H_{14}N_2$ 306.11625, found 306.11588. Elemental Anal. calcd. for $C_{22}H_{14}N_2$: C 86.25, H 4.61, N 9.14 found: C 86.09, H 4.48, N 9.41.

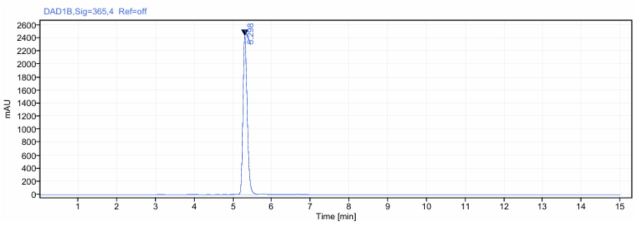

Signal: DAD1B,Sig=365,4 Ref=off

RT [min]	Type	Width [min]	Area	Height	Area%	Name
5.298	BV	0.78	18608.82	2434.82	100.00	
		**Sum**	**18608.82**			

Figure S1. HPLC trace of CCz.

Synthesis of 5,10-dimethyl-5,10-dihydrocarbazolo[3,4-c]carbazole (MeCCz)

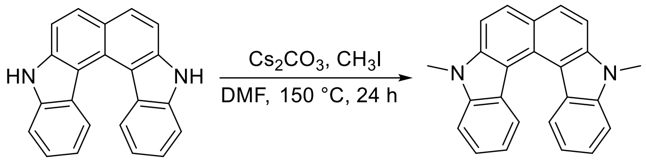

To a solution of 5,10-dihydrocarbazolo[3,4-*c*]carbazole (300 mg, 0.98 mmol) and cesium carbonate (958 mg, 2.94 mmol) in anhydrous DMF (2.5 mL), iodomethane (417 mg, 2.94 mmol) was added dropwise. The reaction mixture was raised up to 150 °C and stirred for 24 h. The mixture was cooled down and poured into water and extracted with CH_2Cl_2 for three times. The combined organic extracts were dried over anhydrous $MgSO_4$ and filtered. The filtrate was concentrated, and the resultant residue was purified through column chromatography (1:1 n-hexane–CH_2Cl_2) to afford the product (213 mg, 65%) as light-yellow solid. ^{1}H NMR (400 MHz, $CDCl_3$, δH): 8.03 (d, J = 8.7 Hz, 2H), 7.92 (d, J = 8.1 Hz, 2H), 7.62 (d, J = 8.7 Hz, 2H), 7.57 (d, J = 8.0 Hz, 2H), 7.48 (td, J = 1.1, 7.0 Hz, 2H), 7.20 (td, J = 1.0, 7.0 Hz, 2H), 4.04 (s, 6H); ^{13}C NMR (100 MHz, $CDCl_3$, δC): 141.1, 140.3, 128.0, 126.2, 124.2, 124.1, 124.0, 123.7, 117.7, 114.6, 108.2, 107.0, 29.5; HRMS (FD) m/z: [M+] calcd. for $C_{24}H_{18}N_2$ 334.14645, found 334.14568. Elemental Anal. calcd. for $C_{24}H_{18}N_2$: C 86.20, H 5.43, N 8.38 found: C 86.05, H 5.33, N 8.32.

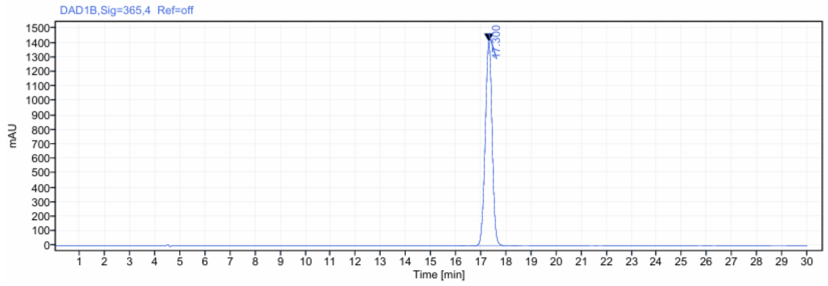

Signal: DAD1B,Sig=365,4 Ref=off

RT [min]	Type	Width [min]	Area	Height	Area%	Name
17.300	BB	2.34	26722.34	1406.56	100.00	
		**Sum**	**26722.34**			

Figure S2. HPLC trace of MeCCz.

Synthesis of 5,10-bis(methyl-d3)-5,10-dihydrocarbazolo[3,4-c]carbazole (MeCCz-d6)

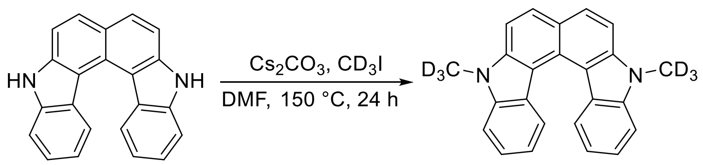

5,10-bis(methyl-d3)-5,10-dihydrocarbazolo[3,4-c]carbazole was prepared in a similar method as 5,10-dimethyl-5,10-dihydrocarbazolo[3,4-c]carbazole from 5,10-dihydrocarbazolo[3,4-c]carbazole (300

mg, 0.98 mmol) and iodomethane-d$_3$ (426 mg, 2.94 mmol). The product was obtained as a yellow solid. (305 mg, 92%) ^{1}H NMR (400 MHz, CDCl$_3$, δH): 8.03 (d, J = 8.8 Hz, 2H), 7.92 (d, J = 8.0 Hz, 2H), 7.62 (d, J = 8.7 Hz, 2H), 7.57 (d, J = 8.0 Hz, 2H), 7.48 (td, J = 1.1, 7.0 Hz, 2H), 7.20 (td, J = 1.0, 7.0 Hz, 2H); ^{13}C NMR (100 MHz, CDCl$_3$, δC): 141.1, 140.3, 128.0, 126.3, 124.2, 124.1, 124.0, 123.7, 117.7, 114.6, 108.2, 107.0, 29.0, 28.8; HRMS (FD) m/z: [M+] calcd. for $C_{24}H_{12}N_2D_6$ 340.18082, found 340.18083. Elemental Anal. calcd. for $C_{24}H_{12}N_2D_6$: C 84.67, H 7.10, N 8.23 found: C 84.66, H 5.27, N 8.19.

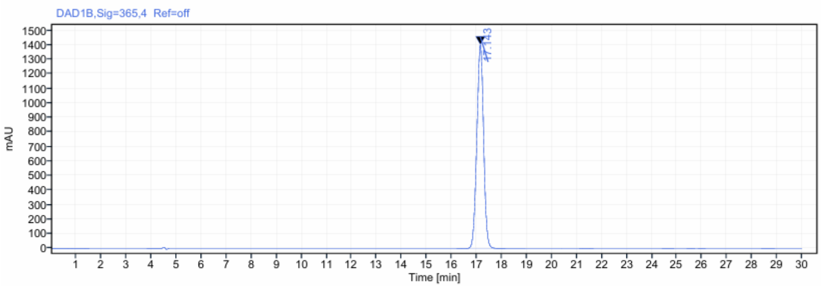

Signal: DAD1B,Sig=365,4 Ref=off

RT [min]	Type	Width [min]	Area	Height	Area%	Name
17.143	VB	2.34	26248.88	1400.86	100.00	
		**Sum**	**26248.88**			

Figure S3. HPLC trace of MeCCz-d6.

Synthesis of 5,10-diphenyl-5,10-dihydrocarbazolo[3,4-c]carbazole (PhCCz)

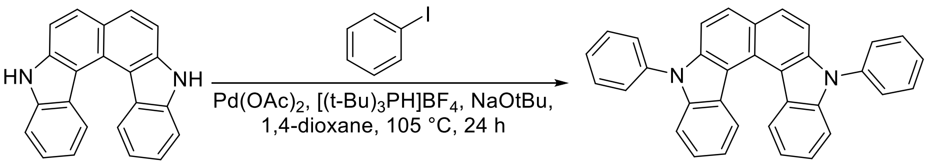

To a mixture of 5,10-dihydrocarbazolo[3,4-*c*]carbazole (3.0 g, 9.8 mmol), sodium *tert*-butoxide (2.8 g, 29.4 mmol), tri-*tert*-butylphosphonium tetrafluoroborate (1.14 g, 3.9 mmol), and palladium acetate (290 mg, 1.3 mmol) were added 1,4-dioxane (98 mL). Iodobenzene (2.2 g, 10.8 mmol) was added dropwise into the mixture. The reaction was stirred under nitrogen atmosphere at 105 °C for 24 h. After cooling the reaction to room temperature, the mixture was filtered through celite eluded with CH_2Cl_2 and concentrated under reduced pressure. The residue was purified by column chromatography (2:1 n-hexane–CH_2Cl_2) to afford the product (2.1 g, 46%) as white solid. ^{1}H NMR (400 MHz, CDCl$_3$, δH): 8.04 (d, J = 8.0 Hz, 2H), 7.93 (d, J = 8.7 Hz, 2H), 7.70-7.65 (m, 8H), 7.57-7.51 (m, 6H), 7.44 (td, J = 1.1, 7.0 Hz, 2H), 7.28 (td, J = 1.1, 7.1 Hz, 2H); ^{13}C NMR (100 MHz, CDCl$_3$, δC): 141.2, 140.5, 137.7, 129.9, 128.1, 127.9, 126.4, 125.2, 124.4, 124.1, 124.0, 118.6, 115.5, 109.5, 108.6; HRMS (FD) m/z: [M+] calcd. for $C_{34}H_{22}N_2$ 458.17775, found 458.17717. Elemental Anal. calcd. for $C_{34}H_{22}N_2$: C 89.05, H 4.84, N 6.11 found: C 88.75, H 4.77, N 6.08.

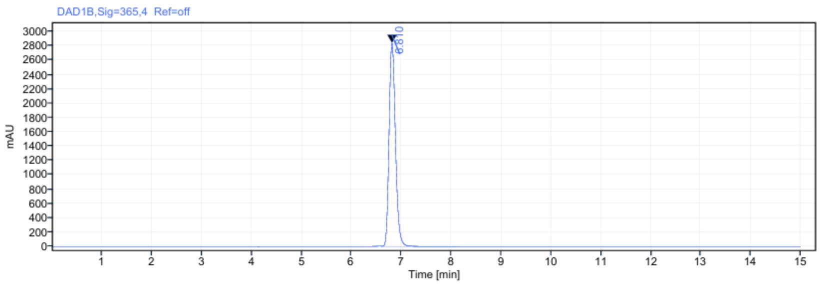

Signal: DAD1B,Sig=365,4 Ref=off

RT [min]	Type	Width [min]	Area	Height	Area%	Name
6.810	VB	1.55	25276.96	2841.95	100.00	
		Sum	25276.96			

Figure S4. HPLC trace of PhCCz.

Synthesis of 9H-carbazole

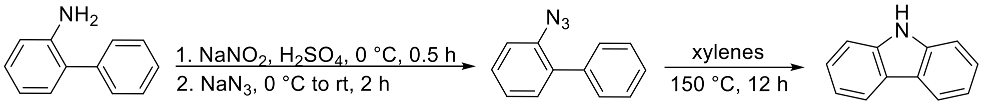

2-Aminobiphenyl (1.7 g, 5.0 mmol) was dissolved in a solution containing 12.0 mL water and 2.0 mL concentrated sulfuric acid. The mixture was cooled down to 0 °C and a solution of sodium nitrite (840 mg, 6.1 mmol) in water (9.4 mL) was added dropwise. The resulting solution was stirred at 0 °C for 30 min. Then, a solution of sodium azide (1.12 g, 8.6 mmol) in water (7 mL) was added dropwise to the cold solution and the mixture stirred for a further 2 h. The reaction was then quenched with 100 mL of a 2 M potassium carbonate solution and extracted with CH_2Cl_2. The organic layer was dried with anhydrous $MgSO_4$ and concentrated under reduced pressure to obtain the crude product. Afterwards, the residue was purified by column chromatography (1:1 n-hexane–CH_2Cl_2) to afford 2-azidobiphenyl (1.94 g, 99%) as a light-yellow oil.

2-Azidobiphenyl (2.0 g, 10.2 mmol) was then dissolved in xylenes (100 mL) and stirred at 150 °C in the sealed tube for 12 h. After the reaction was cooled to room temperature, the solvent was removed under reduced pressure. The residue was recrystallized from toluene to afford 9H-carbazole (1.133 g, 66%) as white sheet crystals.[1] The HPLC result of Lab-Cz was similar to the reported data, which indicates the absence of carbazole isomers, 1*H*-benzo[*f*]indole.[2] ^{1}H NMR (400 MHz, Acetone, δH): 10.32 (s, br, 1H), 8.11 (d, J = 7.8 Hz, 2H), 7.51 (d, J = 8.4 Hz, 2H), 7.38 (t, J = 7.6 Hz, 2H), 7.17 (t, J = 8.1 Hz, 2H); ^{13}C NMR (100 MHz, Acetone, δC): 141.1, 126.5, 124.1, 120.9, 119.7, 111.8.

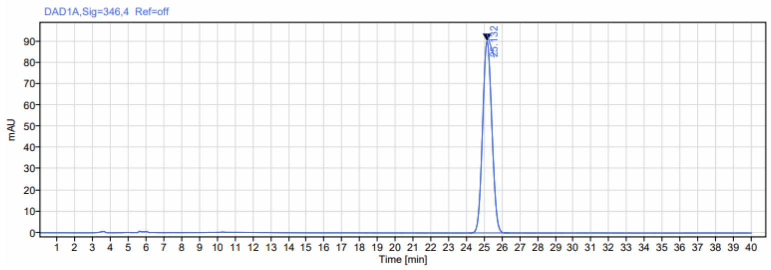

Signal: DAD1A,Sig=346,4 Ref=off

RT [min]	Type	Width [min]	Area	Height	Area%	Name
25.132	BB	2.25	3268.54	90.12	100.00	
		**Sum**	**3268.54**			

Figure S5. HPLC trace of 9*H*-carbazole.

Synthesis of 9-(4-iodophenyl)-9H-carbazole

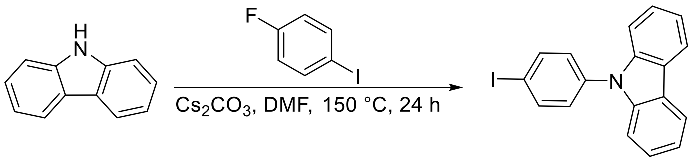

To a solution of 9*H*-carbazole (400 mg, 2.4 mmol) and cesium carbonate (3.118 g, 9.57 mmol) in anhydrous DMF (6 mL), 4-fluoroiodobenzene (2.124 g, 9.57 mmol) was added dropwise. The reaction mixture was raised up to 150 °C and stirred for 24 h. The mixture was cooled down and poured into water and extracted with CH_2Cl_2 for three times. The combined organic extracts were dried over anhydrous $MgSO_4$ and filtered. The filtrate was concentrated, and the resultant residue was purified through column chromatography (8:1 n-hexane–CH_2Cl_2) to afford the product (828 mg, 94%) as white solid. ^{1}H NMR (400 MHz, $CDCl_3$, δH): 8.14 (d, J = 7.6 Hz, 2H), 7.92 (dt, J = 2.2, 8.7 Hz, 2H), 7.43-7.36 (m, 4H), 7.34-7.27 (m, 4H); ^{13}C NMR (100 MHz, $CDCl_3$, δC): 140.5, 139.1, 137.5, 128.9, 126.1, 123.5, 120.4, 120.2, 109.5, 92.0.

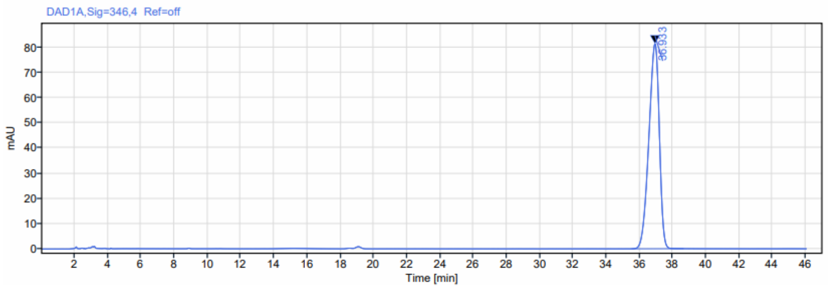

Signal: DAD1A,Sig=346,4 Ref=off

RT [min]	Type	Width [min]	Area	Height	Area%	Name
36.933	BB	3.14	3560.02	81.36	100.00	
		**Sum**	**3560.02**			

Figure S6. HPLC trace of 9-(4-iodophenyl)-9*H*-carbazole.

Synthesis of 5,10-bis(4-(9H-carbazol-9-yl)phenyl)-5,10-dihydrocarbazolo[3,4-c]carbazole (pCzPhCCz)

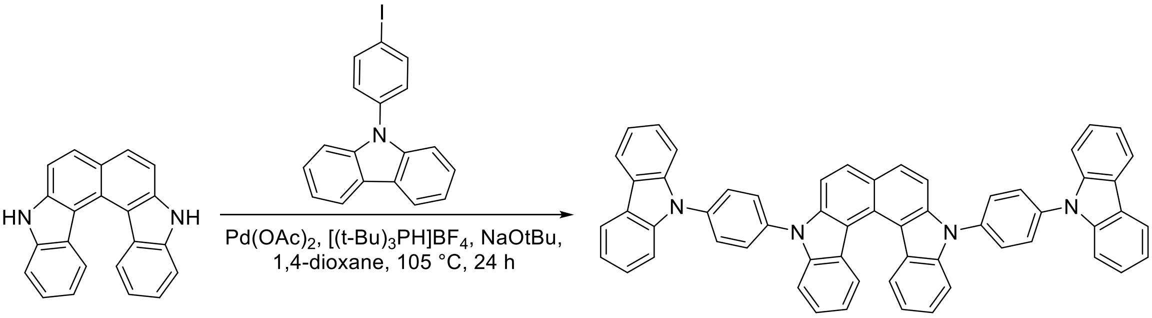

To a mixture of 5,10-dihydrocarbazolo[3,4-*c*]carbazole (300 mg, 0.98 mmol), 9-(4-iodophenyl)-9*H*-carbazole (796 mg, 2.16 mmol), sodium *tert*-butoxide (377 mg, 3.92 mmol), tri-*tert*-butylphosphonium tetrafluoroborate (342 mg, 1.18 mmol), and palladium acetate (88 mg, 0.39 mmol) were added 1,4-dioxane (9.8 mL). The reaction was stirred under nitrogen atmosphere at 105 °C for 24 h. After cooling the reaction to room temperature, the mixture was filtered through celite eluded with CH_2Cl_2 and concentrated under reduced pressure. The residue was purified by column chromatography (1:1 n-hexane–CH_2Cl_2) to afford the product (385 mg, 50%) as white solid. ^{1}H NMR (400 MHz, $CDCl_3$, δH): 8.21 (d, J = 7.8 Hz, 4H), 8.08 (d, J = 7.9 Hz, 2H), 8.04 (d, J = 8.9 Hz, 2H), 7.92 (q, J = 8.6 Hz, 8H), 7.71 (d, J = 8.7 Hz, 2H), 7.69 (d, J = 8.1 Hz, 2H), 7.63 (d, J =8.2 Hz, 4H), 7.54-7.49 (m, 6H), 7.38-7.31 (m, 6H); ^{13}C NMR (100 MHz, $CDCl_3$, δC): 141.1, 140.7, 140.4, 137.3, 136.5, 129.2, 128.4, 126.5, 126.2, 125.5, 124.6, 124.4, 124.0, 123.7, 120.5, 120.4, 119.0, 115.8, 109.8, 109.6, 108.7; HRMS (FD) m/z: [M+] calcd. for $C_{58}H_{36}N_4$ 788.29345, found 788.29424. Elemental Anal. calcd. for $C_{58}H_{36}N_4$: C 88.30, H 4.60, N 7.10 found: C 87.86, H 4.46, N 6.97.

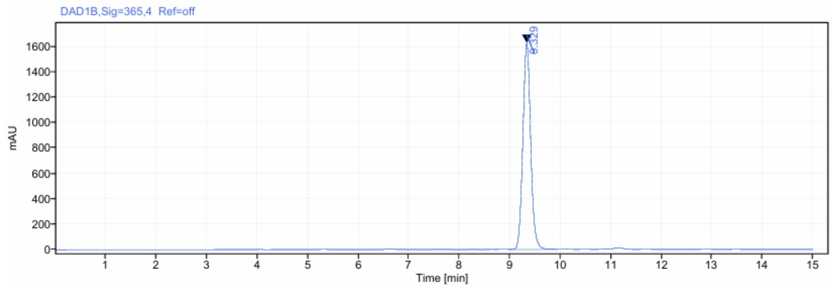

Signal: DAD1B,Sig=365,4 Ref=off

RT [min]	Type	Width [min]	Area	Height	Area%	Name
9.329	VV	0.92	17569.74	1626.60	100.00	
		**Sum**	**17569.74**			

Figure S7. HPLC trace of *p*CzPhCCz.

Synthesis of 4,4'-(carbazolo[3,4-c]carbazole-5,10-diyl)dibenzonitrile (pCNPhCCz)

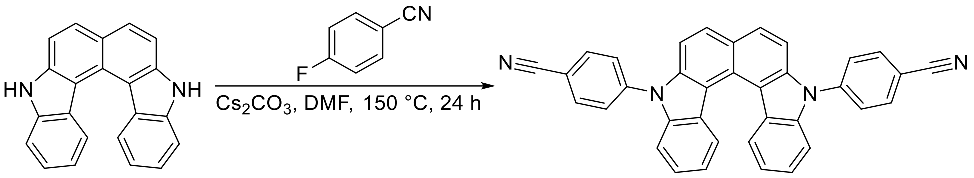

To a mixture of 5,10-dihydrocarbazolo[3,4-*c*]carbazole (400 mg, 1.31 mmol), 4-fluorobenzonitrile (634 mg, 5.23 mmol), and cesium carbonate (1.704 g, 5.23 mmol)were added anhydrous DMF (3.26 mL). The reaction mixture was raised up to 150 °C and stirred for 24 h. After reaction completion, the mixture was added to brine and extracted with CH_2Cl_2. The combined extracts were dried over anhydrous $MgSO_4$ and concentrated under reduced pressure. The residue was purified by column chromatography (1:1 n-hexane–CH_2Cl_2) to afford the product (518 mg, 78%) as light-yellow solid. ^{1}H NMR (400 MHz, $CDCl_3$, δH): 7.97-7.93 (m, 8H), 7.83 (dt, J = 1.9, 6.7 Hz, 4H), 7.54 (d, J = 8.6 Hz, 4H), 7.46 (td, J = 1.1, 7.6 Hz, 2H), 7.28 (td, J = 1.1, 7.6 Hz, 2H); ^{13}C NMR (100 MHz, $CDCl_3$, δC): 141.8, 140.5, 140.2, 139.5, 134.0, 128.5, 128.2, 126.5, 126.0, 124.8, 123.7, 119.6, 118.2, 116.3, 111.3, 109.3, 108.5; HRMS (FD) m/z: [M+] calcd. for $C_{36}H_{20}N_4$ 508.16825, found 508.16842. Elemental Anal. calcd. for $C_{36}H_{20}N_4$: C 85.02, H 3.96, N 11.02 found: C 85.19, H 3.72, N 11.03.

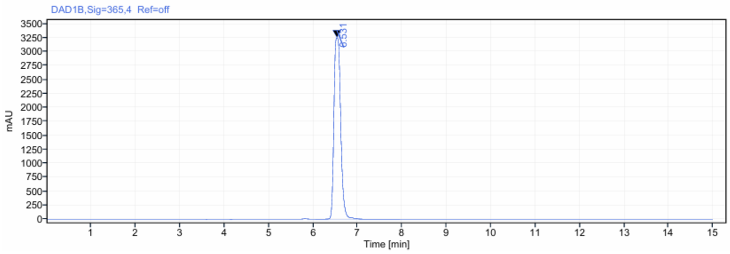

Signal: DAD1B,Sig=365,4 Ref=off

RT [min]	Type	Width [min]	Area	Height	Area%	Name
6.531	VB	1.90	34047.19	3252.88	100.00	
		Sum	34047.19			

Figure S8. HPLC trace of *p*CNPhCCz.

Synthesis of 5,10-bis(2-bromophenyl)-5,10-dihydrocarbazolo[3,4-c]carbazole

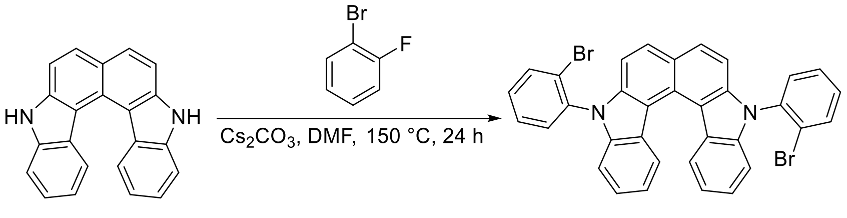

To a solution of 5,10-dihydrocarbazolo[3,4-*c*]carbazole (800 mg, 2.61 mmol)and cesium carbonate (3.4 g, 10.45 mmol) in anhydrous DMF (6.5 mL), 1-bromo-2-fluorobenzene (1.372 g, 7.84 mmol) was added dropwise. The reaction mixture was raised up to 150 °C and stirred for 24 h. The mixture was

extracted with CH_2Cl_2 and washed with brine. The combined organic extracts were dried over anhydrous $MgSO_4$ and filtered. The filtrate was concentrated, and the resultant residue was purified through column chromatography (2:1 n-hexane–CH_2Cl_2) to afford the product (836 mg, 52%) as light-yellow solid. ^{1}H NMR (400 MHz, $CDCl_3$, δH): 8.09 (qd, J = 2.2, 8.5 Hz, 2H), 7.96-7.92 (m, 3H), 7.88 (dt, J = 1.4, 8.1 Hz, 1H), 7.78 (qd, J = 1.6, 7.7 Hz, 1H), 7.64 (tt, J = 1.5, 7.6 Hz, 1H), 7.59-7.40 (m, 7H), 7.30-7.26 (m, 3H), 7.20 (d, J = 8.7 Hz, 1H), 7.17 (dd, J = 3.0, 8.2 Hz, 1H); ^{13}C NMR (100 MHz, $CDCl_3$, δC): 141.3, 141.3, 140.9, 140.5, 140.4, 140.4, 137.0, 136.9, 136.7, 134.3, 134.2, 132.0, 130.9, 130.6, 130.2, 129.9, 128.8, 128.3, 127.9, 126.6, 126.5, 126.4, 125.3, 124.7, 124.3, 124.2, 124.2, 118.9, 118.8, 118.7, 115.7, 115.6, 115.4, 109.7, 108.8, 108.7; HRMS (FD) m/z: [M+] calcd. for $C_{34}H_{20}N_2Br_2$ 613.99987, found 613.99951.

Synthesis of dispiro[fluorene-9,10'-indolo[3,2,1-de]indolo[3',2',1':4,10]acridino[2,3-b]acridine-13',9''-fluorene] (SpiroCCz)

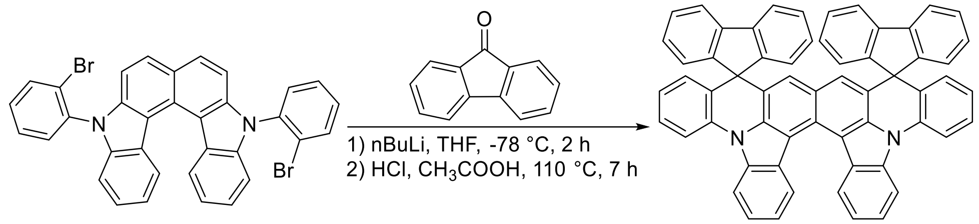

In a two-neck flask, 5,10-bis(2-bromophenyl)-5,10-dihydrocarbazolo[3,4-*c*]carbazole (300 mg, 0.49 mmol) was dissolved in dehydrated THF (4 mL) under nitrogen atmosphere. The solution was cooled down to −78 °C for 10 minutes. Then *n*BuLi (0.73 mL, 1.02 mmol) was added dropwise and the mixture was stirring at −78 °C for 2 h. After that, 9*H*-fluoren-9-one (193 mg, 1.07 mmol) was added, and the mixture was stirred under room temperature overnight. The reaction was quenched with water and extracted with CH_2Cl_2. The combined organic extracts were dried over anhydrous $MgSO_4$, and the solvent was removed under reduced pressure. The residue was dissolved in acetic acid (4 mL) and the solution was heated to 100 °C before concentrated hydrochloric acid (1-2 mL) was added dropwise. The resulting mixture was stirred at 110 °C under reflux for 7 h. After cooling to room temperature, the mixture was poured into ice water and filtered to get crude product. The crude product was purified by column chromatography (3:1 n-hexane–CH_2Cl_2) to afford the product (271 mg, 71%) as yellow solid. ^{1}H NMR (400 MHz, $CDCl_3$, δH): 8.45 (d, J = 8.4 Hz, 2H), 8.34 (d, J = 7.6 Hz, 2H), 8.17 (d, J = 7.7 Hz, 2H), 7.81 (d, J = 7.7 Hz, 2H), 7.75 (d, J = 7.6 Hz, 2H), 7.64 (td, J = 1.1, 7.2 Hz, 2H), 7.44-7.35 (m, 6H), 7.26 (td, J = 1.2, 7.3 Hz, 2H), 7.20-7.16 (m, 4H), 6.97 (td, J = 1.1, 7.8 Hz, 2H), 6.92 (dd, J = 0.8, 7.8 Hz, 2H), 6.86 (td, J = 1.0, 7.5 Hz, 2H), 6.74 (s, 2H), 6.60 (dd, J = 1.4, 7.8 Hz, 2H); ^{13}C NMR (100 MHz, $CDCl_3$, δC): 155.9, 154.3, 140.6, 138.4, 137.6, 137.2, 136.5, 130.1, 128.8, 128.5, 128.4, 128.3, 127.9, 127.7, 127.3, 127.0, 126.2, 124.9, 124.8, 123.8, 123.5, 120.6, 120.2, 120.1, 120.0, 115.6, 115.0, 112.8, 56.9; HRMS (FD) m/z: [M+] calcd. for $C_{60}H_{34}N_2$ 782.27165, found 782.27266. Elemental Anal. calcd. for $C_{60}H_{34}N_2$: C 92.04, H 4.38, N 3.58 found: C 91.60, H 4.22, N 3.64.

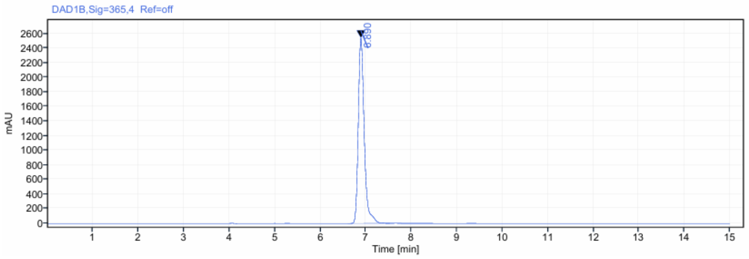

Signal: DAD1B,Sig=365,4 Ref=off

RT [min]	Type	Width [min]	Area	Height	Area%	Name
6.890	BV	0.92	23169.05	2538.04	100.00	
		**Sum**	**23169.05**			

Figure S9. HPLC trace of SpiroCCz.

II. Thermal Properties

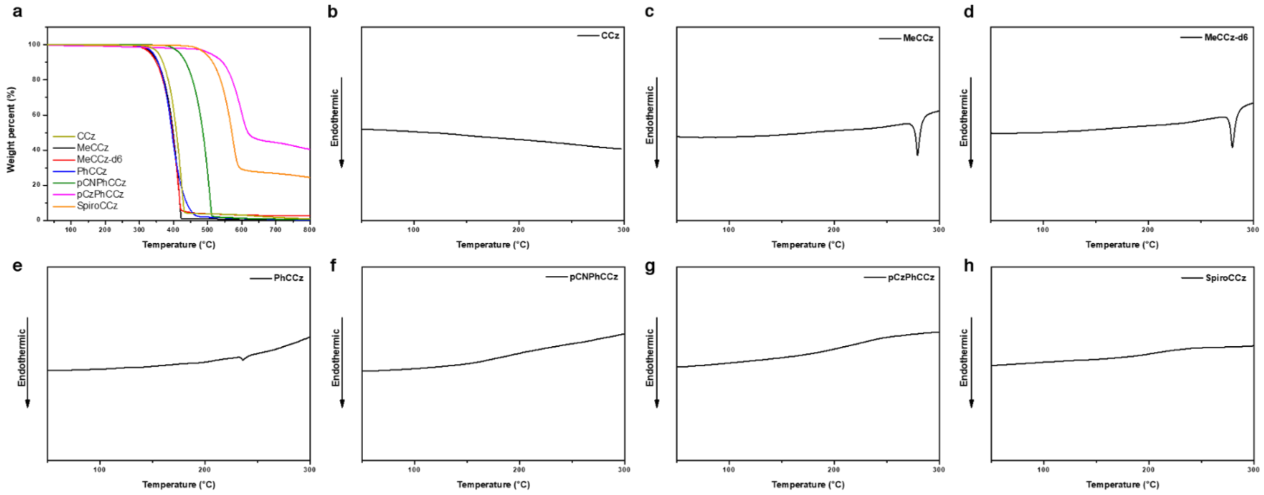

Figure S10. (a) Thermal gravimetric analysis (TGA) for all CCz emitters. (b) - (h) The differential scanning calorimetry (DSC) for all CCz emitters.

Table S1. Summary of the thermal physical properties of CCz compounds.

Compound	T_d (°C) [a]	T_g (°C) [b]	T_m (°C) [b]
CCz	356	-	-
MeCCz	336	-	279
MeCCz-d6	332	-	280
PhCCz	341	236	-
*p*CNPhCCz	419	-	-
*p*CzPhCCz	508	-	-
SpiroCCz	494	-	-

[a] Decomposition Temperature (T_d) corresponding to 5% weight loss. [b] Glass transition temperature (T_g) and melting point (T_m).

III. Single-Crystal Analysis

Table S2. The crystal data and structure refinements of five CCz compounds

Compound	CCz	MeCCz	MeCCz-d6	*p*CNPhCCz	SpiroCCz
Empirical formula	$C_{44}H_{28}N_4$	$C_{24}H_{18}N_2$	$C_{24}H_{12}D_6N_2$	$C_{36}H_{20}N_4$	$C_{60}H_{34}N_2$
Formula weight	612.70	334.40	340.44	508.56	782.89
Temperature/K	99.99(10)	100.00(10)	99.99(17)	100.00(10)	100.00(10)
Crystal system	triclinic	monoclinic	monoclinic	triclinic	orthorhombic
Growth	Solution (DMF)	Solution (Tol)	Solution (Tol)	Sublimation	Sublimation
Space group	P-1	$P2_1/c$	$P2_1/c$	P-1	$P2_12_12_1$
a/Å	10.9055(4)	10.03952(17)	10.0406(3)	10.12470(10)	37.10347(12)
b/Å	11.6274(3)	6.44071(11)	6.4322(2)	11.1321(2)	25.77824(7)
c/Å	11.9751(5)	26.3167(5)	26.3065(8)	12.9505(2)	16.70064(5)
α/°	90.236(2)	90	90	109.4480(10)	90
β/°	93.558(3)	100.6011(16)	100.679(3)	105.2530(10)	90
γ/°	105.336(3)	90	90	104.8890(10)	90
Volume/Å^3	1461.25(9)	1672.63(5)	1669.53(9)	1228.96(3)	15973.53(8)
Z	2	4	4	2	16
ρ_{calc}g/cm^3	1.393	1.328	1.354	1.374	1.302
μ/mm^{-1}	0.639	0.602	0.603	0.641	0.577
F(000)	640.0	704.0	704.0	528.0	6528.0
Crystal size/mm^3	0.23 × 0.22 × 0.13	0.08 × 0.07 × 0.03	0.07 × 0.03 × 0.01	0.23 × 0.2 × 0.15	0.09 × 0.07 × 0.03
Radiation	Cu Kα (λ = 1.54184)	Cu Kα (λ = 1.54184)	Cu Kα (λ = 1.54184)	Cu Kα (λ = 1.54184)	Cu Kα (λ = 1.54184)
2Θ range for data collection/°	7.398 to 134.15	6.834 to 134.152	6.838 to 154.726	7.836 to 153.98	4.174 to 149.7
Index ranges	-12 ≤ h ≤ 13<br>-11 ≤ k ≤ 13<br>-14 ≤ l ≤ 14	-11 ≤ h ≤ 11<br>-7 ≤ k ≤ 7<br>-31 ≤ l ≤ 31	-12 ≤ h ≤ 12<br>-7 ≤ k ≤ 5<br>-32 ≤ l ≤ 32	-12 ≤ h ≤ 12<br>-12 ≤ k ≤ 14<br>-16 ≤ l ≤ 13	-45 ≤ h ≤ 45<br>-23 ≤ k ≤ 32<br>-19 ≤ l ≤ 20
Reflections collected	20001	8711	16890	13488	240331
Independent reflections	5197 [R_{int} = 0.0310, R_{sigma} = 0.0255]	2979 [R_{int} = 0.0182, R_{sigma} = 0.0217]	3419 [R_{int} = 0.0526, R_{sigma} = 0.0234]	4915 [R_{int} = 0.0135, R_{sigma} = 0.0137]	31643 [R_{int} = 0.0292, R_{sigma} = 0.0177]
Data/restraints/parameters	5197/0/433	2979/0/238	3419/0/237	4915/0/362	31643/0/2235

Goodness-of-fit on F^2	1.034	1.079	1.121	1.035	1.019
Final R indexes [I>=2σ (I)]	R_1 = 0.0786, wR_2 = 0.2011	R_1 = 0.0376, wR_2 = 0.0980	R_1 = 0.0487, wR_2 = 0.1159	R_1 = 0.0346, wR_2 = 0.0878	R_1 = 0.0342, wR_2 = 0.0900
Final R indexes [all data]	R_1 = 0.0813, wR_2 = 0.2056	R_1 = 0.0409, wR_2 = 0.1004	R_1 = 0.0603, wR_2 = 0.1341	R_1 = 0.0356, wR_2 = 0.0885	R_1 = 0.0389, wR_2 = 0.0929
Largest diff. peak/hole / e Å^{-3}	0.92/-0.54	0.30/-0.32	0.26/-0.26	0.26/-0.22	0.24/-0.17

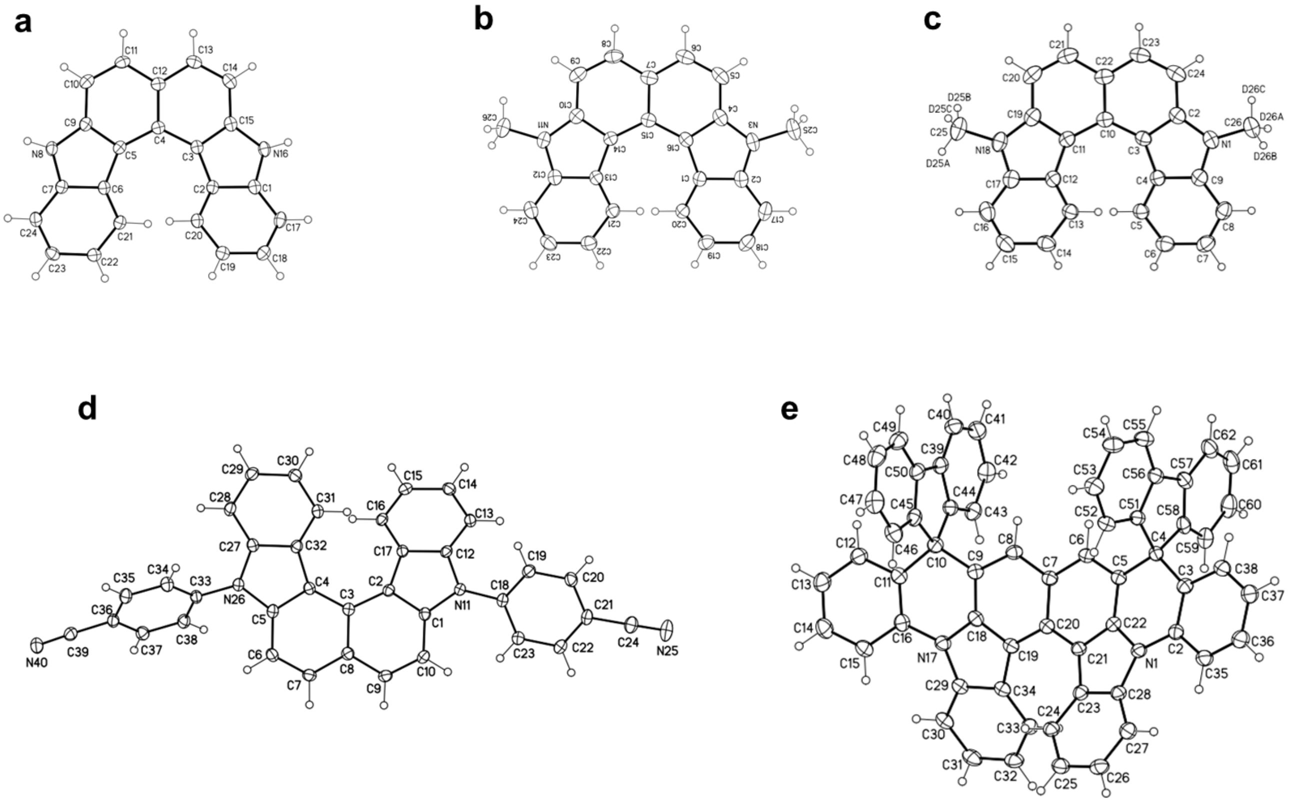

Figure S11. ORTEP plots of (a) CCz, (b) MeCCz, (c) MeCCz-d6, (d) *p*CNPhCCz, and (e) SpiroCCz with 50% thermal ellipsoids.

IV. Theoretical Calculations

The structure optimization and frequency analysis of five molecules were calculated with Gaussian 16.[3] The ground state (S_0) geometries were optimized using the density functional theory (DFT). The time-dependent density functional theory (TD-DFT) with the Tamm-Dancoff approximation (TDA) was adopted to optimize the geometries of the first excited singlet (S_1) and triplet (T_1) states.[4] We employed the polarizable continuum model (PCM)[5] and the two-layer ONIOM model[6] of the quantum mechanics/molecular mechanics (QM/MM) method to assess basic photophysical data in toluene and the thin film environments, respectively. As shown in **Figure S12**, the innermost molecule was found to be the higher layer by the accurate high-level QM method, and the outermost molecules were treated as the lower layer using the efficient universal force field (UFF) method.[7] In the QM/MM geometry optimization process, only the atoms in the QM part can move and others in the MM part were frozen. The initial geometries of the five molecules in the solid phase were based on the measured crystalline structures.

Several functionals with different percentages of Hartree-Fock exchange (HF%), including B3LYP (20%), PBE0 (25%), BMK (42%), M06-2X (54%), CAM-B3LYP (19% HF at short range and 65% HF at long range), and ωB97XD (22.2% HF at short range and 100% HF at long range) are tested.[4, 8, 9] Different functionals were used to calculate fluorescence emission wavelengths, and these wavelengths changed significantly. It was found that the best approach for calculating the fluorescence peak (λ_{Fl}), phosphorescence peak (λ_{Ph}), and the singlet-triplet gap (ΔE_{ST}) was B3LYP/6-31G (d). We carried out calculations of the adiabatic ΔE_{ST}, which are defined as the energy differences between the minima potential energies of the S_1 and T_1 states. Vibrational frequency analyses were also carried out to confirm the local minima which showed no imaginary frequency. Many previous studies [4, 8] on emitters suggest that basis sets show no obvious effect on excitation energies. Thus we choose the 6-31G(d) basis set to save on computational costs.[9, 10] Here, we neglected Zero-Point Vibrational

Energies (ZPVE) between ground- and excited-states, because it is independent of functional and basis sets.[11] Besides, ZPVE is much smaller in comparison with the excitation energies.[12] The spin-orbit coupling (SOC) matrix between S_1 (S_0) and T_n (n=1-3) states were calculated with the optimized geometries of S_1 and T_1 by the ORCA program, respectively.[13] And oscillator strength of $T_1 \rightarrow S_0$ was obtained by Dalton package.[14]

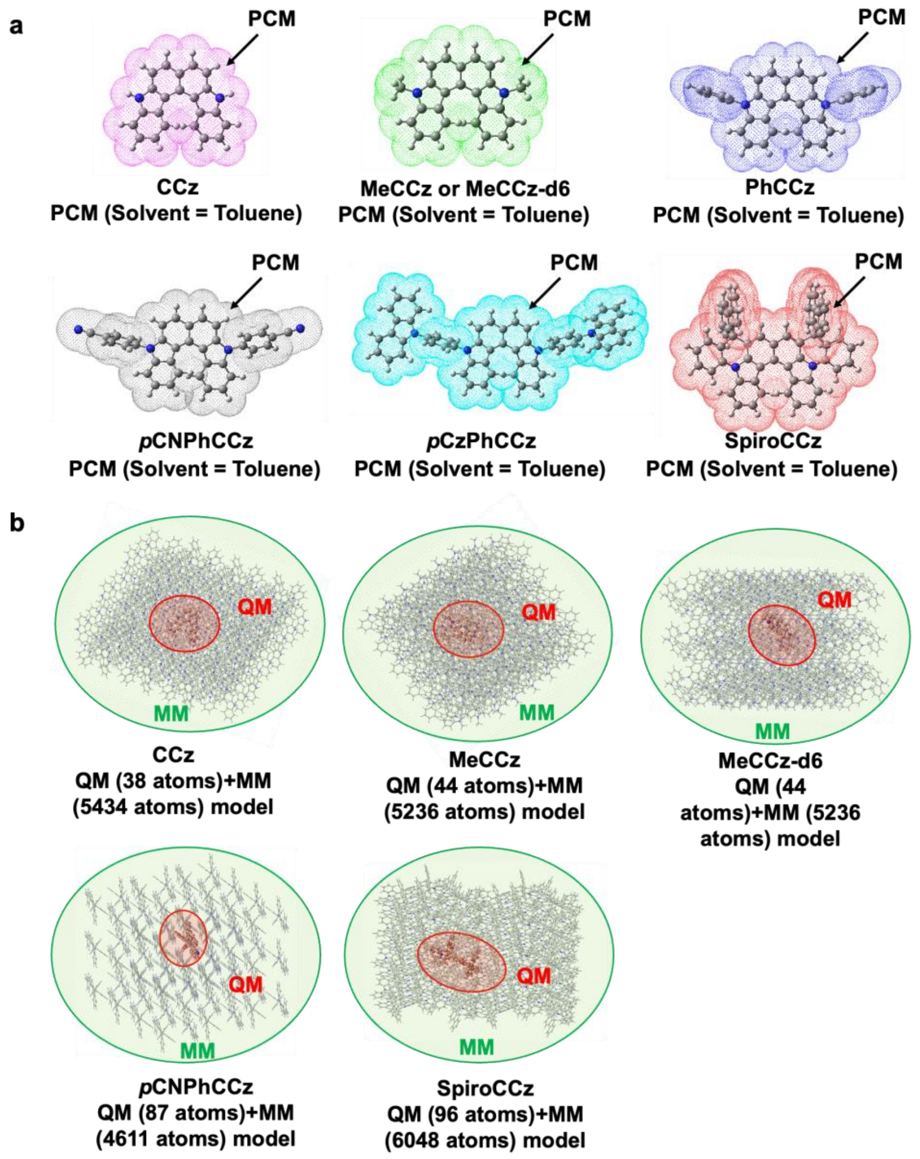

Figure S12. (a) PCM model: (solvent = toluene) for the seven molecules. (b) ONIOM model: single CCz, MeCCz, MeCCz-d6, *p*CNPhCCz, or SpiroCCz molecule is treated as the high layer (QM), respectively, and surrounding molecules are regarded as the low layer (MM).

Table S3. Calculated excited-state characteristics of seven molecules in toluene.

Method (Solid)	λ_{Fl}[a] [nm]	λ_{Ph}[b] [nm]	$\Delta E(S_1)$[c] [eV]	$\Delta E(T_1)$[c] [eV]	ΔE_{ST}[d] [eV]	HOMO[e] [eV]	LUMO[e] [eV]	ΔE_g[e] [eV]
				CCz				
B3LYP	390	549	3.38	2.52	0.86	-4.90	-1.28	3.62
CAM-B3LYP	352	523	3.81	2.70	1.11	-6.04	-0.20	5.84
PBE0	376	539	3.51	2.57	0.94	-5.16	-1.21	3.95
BMK	358	503	3.71	2.78	0.93	-5.55	-0.71	4.85
M06-2X	349	477	3.83	2.94	0.89	-6.03	-0.56	5.47
ωB97X-D	350	513	3.84	2.76	1.08	-6.64	0.31	6.95
				MeCCz or MeCCz-d6				
B3LYP	397	549	3.32	2.49	0.83	-4.84	-1.26	3.58
CAM-B3LYP	358	528	3.74	2.67	1.07	-5.98	-0.19	5.80
PBE0	383	545	3.44	2.53	0.90	-5.10	-1.20	3.91
BMK	365	508	3.64	2.74	0.89	-5.49	-0.69	4.80
M06-2X	355	482	3.74	2.90	0.85	-5.98	-0.56	5.43
ωB97X-D	355	517	3.76	2.72	1.04	-6.58	0.32	6.91
				PhCCz				
B3LYP	396	551	3.31	2.51	0.81	-4.94	-1.34	3.60
CAM-B3LYP	357	523	3.75	2.69	1.06	-6.07	-0.26	5.81
PBE0	383	541	3.43	2.55	0.88	-5.22	-1.29	3.93
BMK	365	506	3.63	2.76	0.88	-5.60	-0.79	4.81
M06-2X	355	479	3.74	2.91	0.82	-6.10	-0.66	5.44
ωB97X-D	353	512	3.78	2.74	1.03	-6.67	-0.25	6.42
				*p*CNPhCCz				
B3LYP	496	563	2.84	2.45	0.39	-5.15	-2.15	3.00
CAM-B3LYP	361	532	3.71	2.65	1.06	-6.36	-0.70	5.65
PBE0	452	553	3.09	2.50	0.59	-5.43	-2.05	3.38
BMK	371	514	3.56	2.72	0.84	-5.90	-1.25	4.65
M06-2X	360	486	3.69	2.87	0.82	-6.38	-1.09	5.29
ωB97X-D	357	519	3.75	2.71	1.04	-6.96	-0.19	6.76
				*p*CzPhCCz				
B3LYP	399	553	3.27	2.50	0.78	-5.15	-1.47	3.69
CAM-B3LYP	334	525	3.85	2.68	1.17	-6.36	-0.40	5.95
PBE0	373	544	3.45	2.54	0.91	-5.43	-1.40	4.03
BMK	348	507	3.69	2.75	0.95	-5.84	-0.87	4.98

M06-2X	338	480	3.81	2.91	0.90	-6.35	-0.73	5.62
ωB97X-D	331	513	3.88	2.74	1.14	-6.96	0.20	7.16
				SpiroCCz				
B3LYP	418	672	3.07	2.13	0.94	-5.06	-1.53	3.53
CAM-B3LYP	372	565	3.55	2.52	1.02	-6.09	-0.46	5.63
PBE0	402	585	3.19	2.38	0.81	-5.32	-1.47	3.85
BMK	381	540	3.41	2.59	0.82	-5.65	-0.98	4.68
M06-2X	369	514	3.52	2.75	0.77	-6.16	-0.84	5.31
ωB97X-D	366	546	3.60	2.60	1.00	-6.69	0.06	6.75

[a] Fluorescence peaks (λ_{Fl}). [b] Phosphorescence peaks (λ_{Ph}). [c] The adiabatic energies of the lowest excited singlet state ($\Delta E(S_1)$) and triplet state ($\Delta E(T_1)$). [d] The singlet-triplet splitting energy (ΔE_{ST}). [e] HOMO and LUMO orbital energies, and the energy gap (E_g) between HOMO and LUMO.

Table S4. Calculated excited-state characteristics of five molecules in the solid phase.

Method (Solid)	λ_{Fl}[a] [nm]	λ_{Ph}[b] [nm]	$\Delta E(S_1)$[c] [eV]	$\Delta E(T_1)$[c] [eV]	ΔE_{ST}[d] [eV]	HOMO[e] [eV]	LUMO[e] [eV]	ΔE_g[e] [eV]
				CCz				
B3LYP	391	562	3.34	2.47	0.88	-4.93	-0.96	3.97
CAM-B3LYP	338	519	3.88	2.70	1.18	-6.18	0.20	6.38
PBE0	365	534	3.56	2.57	0.99	-5.24	-0.85	4.39
BMK	346	499	3.77	2.78	1.00	-5.70	-0.31	5.39
M06-2X	334	474	3.89	2.94	0.95	-6.18	-0.18	6.00
ωB97X-D	335	508	3.90	2.75	1.15	-6.76	0.73	7.50
				MeCCz				
B3LYP	391	562	3.34	2.95	0.40	-4.93	-0.96	3.97
CAM-B3LYP	350	538	3.78	2.64	1.14	-6.04	0.29	6.33
PBE0	377	553	3.47	2.51	0.96	-5.09	-0.74	4.35
BMK	359	517	3.67	2.71	0.96	-5.55	-0.21	5.34
M06-2X	348	491	3.79	2.87	0.92	-6.03	-0.09	5.95
ωB97X-D	348	526	3.81	2.70	1.11	-6.62	0.83	7.45
				MeCCz-d6				
B3LYP	391	562	3.34	2.47	0.88	-4.93	-0.96	3.97
CAM-B3LYP	350	538	3.78	2.64	1.14	-6.04	0.29	6.33
PBE0	377	553	3.47	2.51	0.96	-5.10	-0.73	4.37
BMK	359	517	3.67	2.71	0.96	-5.57	-0.21	5.36

M06-2X	491	348	3.79	2.87	0.92	-6.05	-0.08	5.98
ωB97X-D	348	526	3.81	2.70	1.11	-6.64	0.84	7.48
				*p*CNPhCCz				
B3LYP	459	574	2.96	2.87	0.09	-5.46	-1.71	3.75
CAM-B3LYP	355	543	3.75	2.62	1.13	-6.66	-0.55	6.11
PBE0	421	563	3.20	2.47	0.73	-5.09	-0.74	4.35
BMK	366	524	3.61	2.68	0.93	-6.19	-1.08	5.11
M06-2X	353	495	3.74	2.85	0.89	-6.66	-0.92	5.74
ωB97X-D	350	529	3.79	2.68	1.11	-6.62	0.83	7.45
				SpiroCCz				
B3LYP	409	590	3.11	2.34	0.77	-5.00	-1.24	3.76
CAM-B3LYP	361	562	3.59	2.52	1.07	-6.19	-0.16	6.03
PBE0	394	581	3.23	2.38	0.85	-5.25	-1.18	4.07
BMK	371	542	3.45	2.58	0.87	-5.66	-0.64	5.02
M06-2X	360	514	3.55	2.73	0.82	-6.17	-0.55	5.62
ωB97X-D	356	548	3.63	2.58	1.05	-6.77	0.36	7.13

[a] Fluorescence peaks (λ_{fl}). [b] Phosphorescence peaks (λ_{ph}). [c] The adiabatic energies of the lowest excited singlet state ($\Delta E(S_1)$) and triplet state ($\Delta E(T_1)$). [d] The singlet-triplet splitting energy (ΔE_{ST}). [e] HOMO and LUMO orbital energies, and the energy gap (E_g) between HOMO and LUMO.

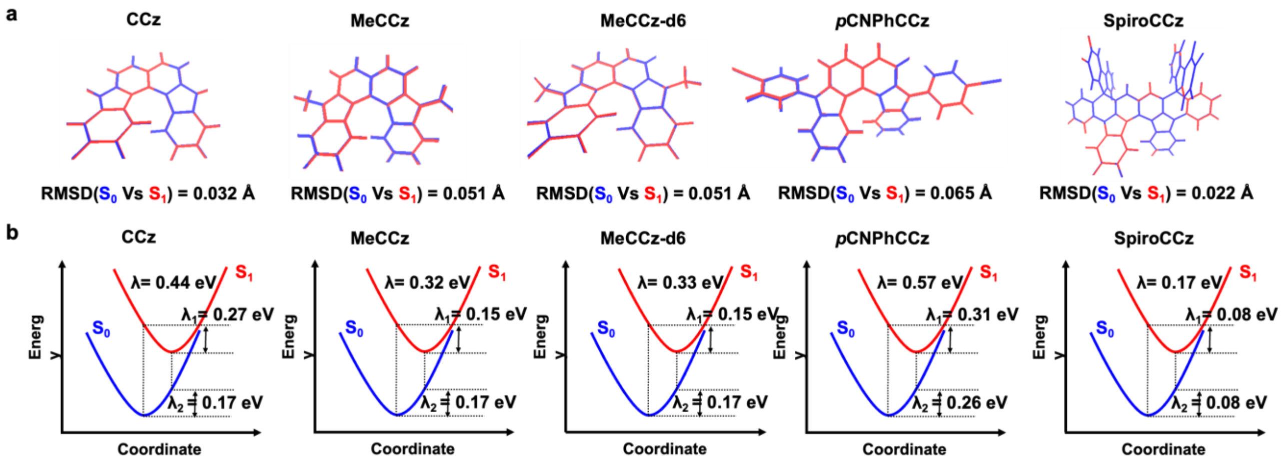

Figure S13. (a) Calculated root-mean-square-deviation (RMSD) and (b) reorganization energy (λ) between the optimized S_0 (blue) and S_1 (blue) geometries in the solid phase at B3LYP/6-31G(d) level.

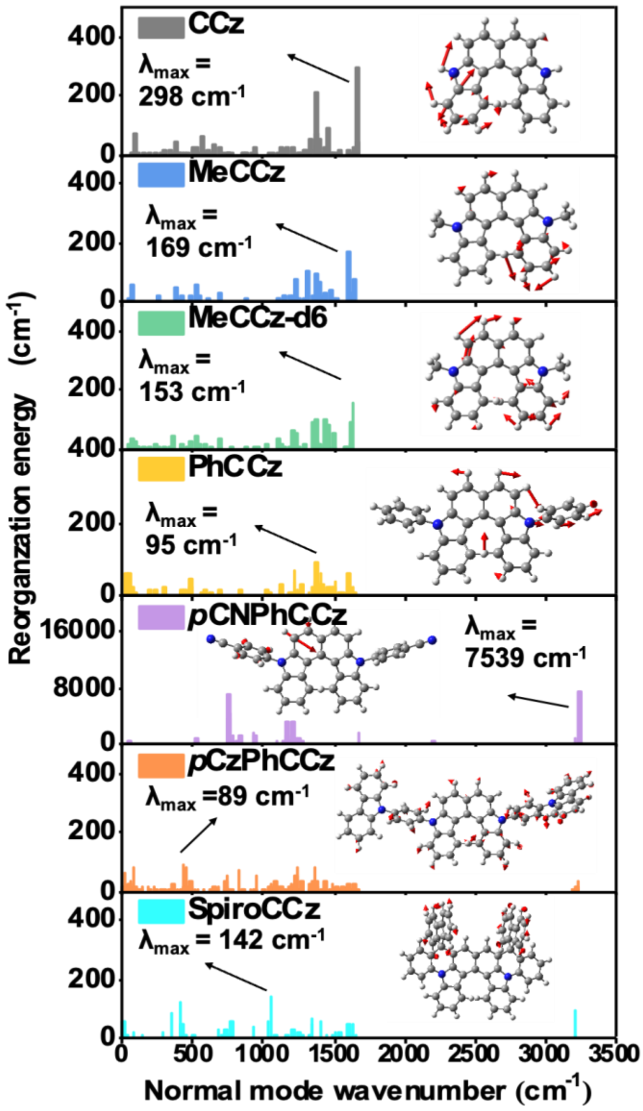

Figure S14. Normal mode reorganization energy between S_0 and S_1 based on the optimized S_1 geometries in the solid phase at B3LYP/6-31G(d) level.

As shown in **Figure S14**, we depicted reorganization energy in the S_1 to S_0 process for each normal mode. Interestingly, the maximum contribution of reorganization energy (λ_{max}) for CCz, MeCCz, MeCCz-d6, PhCCz, *p*CNPhCCz, *p*CzPhCCz, and SpiroCCz are 298, 169, 153, 95, 7536, 89, 142 cm^{-1}, respectively. Obviously, λ_{max} of *p*CNPhCCz is largest than other molecules, due to charge transfer in S_1. Compared with CCz, λ_{max} of SpiroCCz is smaller with spiro stretching vibration. Therefore, introducing spiro units can decrease reorganization energy and suppress nonradiative decay process of S_1.

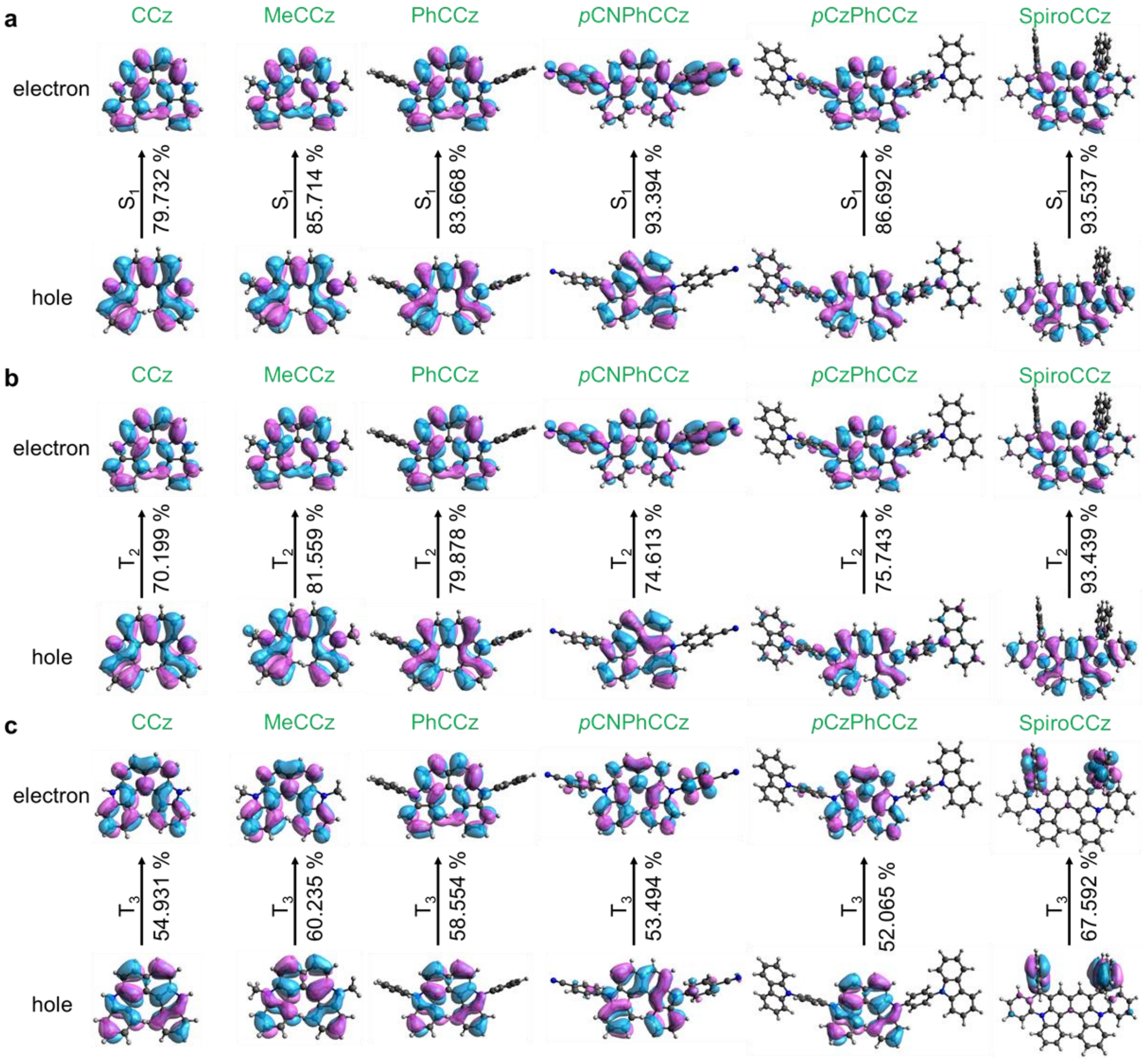

Figure S15. Natural transition orbitals (NTOs) for S_1, T_2, and T_3 based on the optimized S_0 geometries in toluene at B3LYP/6-31G(d) level.

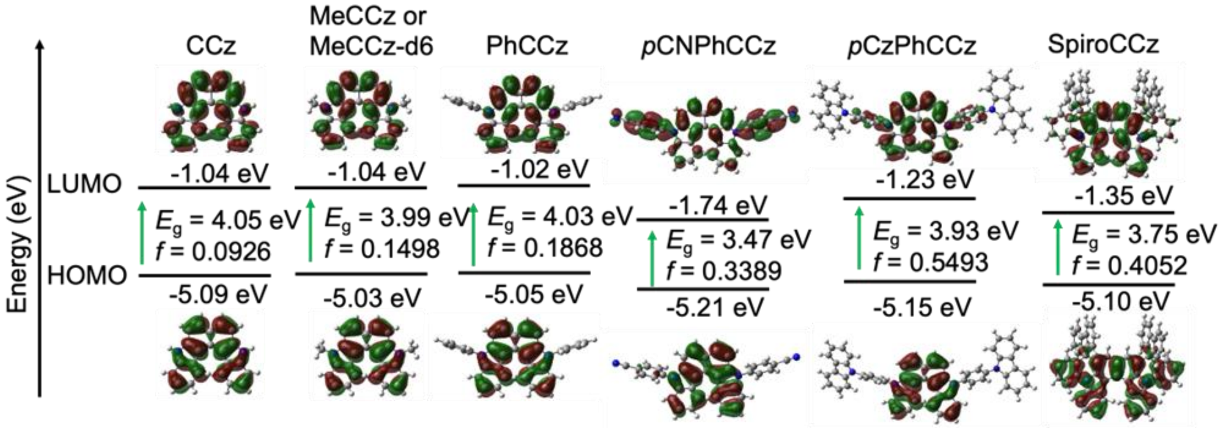

Figure S16. HOMO/LUMO levels, energy gaps (E_g), and oscillator strength based on the optimized S_1 geometries in toluene at B3LYP/6-31G(d) level.

Table S5. Calculated vertical absorption properties of the triplet states for seven molecules.

State	Main configuration	CI coefficient	E_{abs} (eV)[a]	λ_{abs} (nm)[b]	f[c]
		CCz			
	HOMO-3→LUMO+1	-0.12778			
T_1	HOMO-1→LUMO+1	-0.11411	2.5797	480.61	0.0000
	HOMO→LUMO	0.65217			
	HOMO-4→LUMO+3	0.10385			
	HOMO-3→LUMO	-0.18411			
T_2	HOMO-2→LUMO+2	-0.10645	3.0345	408.59	0.0000
	HOMO-1→LUMO	0.59166			
	HOMO→LUMO+1	0.25636			
	HOMO-3→LUMO	-0.25181			
T_3	HOMO-1→LUMO	-0.33999	3.1946	388.11	0.0000
	HOMO→LUMO+1	0.52085			
		MeCCz or MeCCz-d6			
	HOMO-3→LUMO+1	-0.12649			
T_1	HOMO-2→LUMO+1	-0.10718	2.5412	487.89	0.0000
	HOMO→LUMO	0.65544			
	HOMO-3→LUMO	-0.13954			
T_2	HOMO-2→LUMO	0.63875	2.9630	418.44	0.0000
	HOMO→LUMO+1	0.18626			

	HOMO-3→LUMO	-0.28670			
T_3	HOMO-2→LUMO	-0.25046	3.1570	392.73	0.0000
	HOMO→LUMO+1	0.54524			
		PhCCz			
T_1	HOMO-3→LUMO+1	-0.11595	2.5738	481.73	0.0000
	HOMO→LUMO	0.65475			
	HOMO-3→LUMO	-0.15012			
T_2	HOMO-2→LUMO	0.63102	3.0098	411.93	0.0000
	HOMO→LUMO+1	0.17829			
	HOMO-3→LUMO	-0.28930			
	HOMO-1→LUMO	-0.26597			
T_3	HOMO-1→LUMO+7	-0.10048	3.2016	387.25	0.0000
	HOMO→LUMO+1	0.49532			
	HOMO→LUMO+3	0.17015			
	HOMO→LUMO+5	0.11438			
		*p*CNPhCCz			
	HOMO→LUMO	0.12233			
T_1	HOMO→LUMO	0.57137	2.1562	575.02	0.0000
	HOMO→LUMO+2	-0.32383			
	HOMO-1→LUMO	0.57580			
T_2	HOMO-1→LUMO+2	-0.24680	2.8162	440.26	0.0000
	HOMO→LUMO	-0.16564			
	HOMO→LUMO+5	-0.12041			
	HOMO-3→LUMO	-0.24913			
	HOMO-3→LUMO+2	0.16727			
	HOMO-2→LUMO	-0.14445			
	HOMO-1→LUMO	0.21749			
T_3	HOMO→LUMO+2	-0.10128	2.9437	421.18	0.0000
	HOMO→LUMO+3	-0.12120			
	HOMO→LUMO+4	0.23845			
	HOMO→LUMO+5	0.41136			
		*p*CzPhCCz			
	HOMO-7→LUMO+5	-0.12913			
T_1	HOMO→LUMO	0.63592	2.5444	487.28	0.0000
	HOMO→LUMO+4	-0.10266			
T_2	HOMO-7→LUMO	-0.13321	2.9766	416.53	0.0000
	HOMO-3→LUMO	0.16916			

	HOMO-1→LUMO	0.60875			
	HOMO→LUMO+5	0.17178			
	HOMO-7→LUMO	-0.29217			
T_3	HOMO-1→LUMO	-0.23478	3.1539	393.11	0.0000
	HOMO→LUMO+3	-0.10118			
	HOMO→LUMO+5	0.49805			
		SpiroCCz			
T_1	HOMO-2→LUMO	0.64881	2.3946	517.77	0.0000
	HOMO→LUMO+3	0.10921			
T_2	HOMO→LUMO	0.68254	2.7905	444.30	0.0000
	HOMO-6→LUMO+2	0.10046			
	HOMO-4→LUMO+1	0.39397			
T_3	HOMO-2→LUMO+2	0.40339	3.0366	408.30	0.0000
	HOMO-1→LUMO+1	-0.17089			
	HOMO-1→LUMO+3	0.13238			

[a] Vertical excitation energy. [b] Excitation wavelength. [c] Oscillator strength. Calculations are based on S_0 geometries by TD-DFT at the B3LYP/6-31G(d) in toluene

Table S6. Calculated vertical absorption properties of the singlet states for seven molecules.

State	Main configuration	CI coefficient	E_{abs} (eV)[a]	λ_{abs} (nm)[b]	f[c]
		CCz			
	HOMO-1→LUMO	0.62765			
S_1	HOMO→LUMO+1	0.29408	3.5539	348.86	0.0926
	HOMO→LUMO+2	-0.10402			
	HOMO-7→LUMO	-0.12322			
S_2	HOMO-5→LUMO	-0.11635	3.5647	347.82	0.1377
	HOMO-1→LUMO	0.67860			
	HOMO-9→LUMO	-0.14050			
S_3	HOMO-8→LUMO	0.56841	4.0499	306.14	0.7008
	HOMO-4→LUMO	0.14083			
	HOMO-2→LUMO	0.35084			

		MeCCz or MeCCz-d6			
S_1	HOMO-1→LUMO<br>HOMO→LUMO+1	0.64739<br>0.25235	3.4600	358.34	0.1498
S_2	HOMO-1→LUMO<br>HOMO→LUMO+1	-0.21667<br>0.66593	3.5004	354.20	0.1238
S_3	HOMO-1→LUMO<br>HOMO→LUMO+1	-0.24879<br>0.65439	4.0097	309.21	0.7133
		PhCCz			
S_1	HOMO-1→LUMO<br>HOMO→LUMO+1	0.64668<br>0.23644	3.5106	353.17	0.1868
S_2	HOMO-1→LUMO<br>HOMO→LUMO+1	-0.20067<br>0.66741	3.5410	350.14	0.1328
S_3	HOMO-1→LUMO<br>HOMO→LUMO+1	-0.23508<br>0.65889	4.0342	307.33	0.7363
		*p*CNPhCCz			
S_1	HOMO-1→LUMO<br>HOMO→LUMO	-0.13699<br>0.68177	3.0182	410.79	0.3389
S_2	HOMO-1→LUMO<br>HOMO→LUMO+1<br>HOMO→LUMO	0.24251<br>0.64346	3.1912	388.52	0.0123
S_3	HOMO-1→LUMO<br>HOMO→LUMO<br>HOMO→LUMO+1<br>HOMO→LUMO+5<br>HOMO→LUMO+3	0.60119<br>0.11304<br>-0.27600<br>0.12347<br>-0.11199	3.2381	382.90	0.3894
		*p*CzPhCCz			
S_1	HOMO-3→LUMO<br>HOMO-1→LUMO<br>HOMO→LUMO+5	0.10514<br>0.65788<br>0.17551	3.4109	363.50	0.5493
S_2	HOMO-1→LUMO+5<br>HOMO→LUMO	-0.18374<br>0.66791	3.5015	354.09	0.1403
S_3	HOMO-3→LUMO<br>HOMO-2→LUMO+3<br>HOMO-1→LUMO+4<br>HOMO→LUMO+1<br>HOMO→LUMO+3<br>HOMO→LUMO+5	-0.15843<br>0.15626<br>0.42133<br>-0.12666<br>0.37558<br>-0.25704	3.7936	326.83	0.9204

			SpiroCCz		
S_1	HOMO-1→LUMO+3	-0.14202	3.1682	391.34	0.4052
	HOMO→LUMO	0.68357			
S_2	HOMO-1→LUMO	0.67542	3.3484	370.28	0.1370
	HOMO→LUMO+3	0.17226			
S_3	HOMO-2→LUMO	-0.20733	3.7053	334.61	0.0089
	HOMO→LUMO+1	0.64111			
	HOMO→LUMO+3	-0.15931			

[a] Vertical excitation energy. [b] Excitation wavelength. [c] Oscillator strength. Calculations are based on S_0 geometries by TD-DFT at the B3LYP/6-31G(d) in toluene.

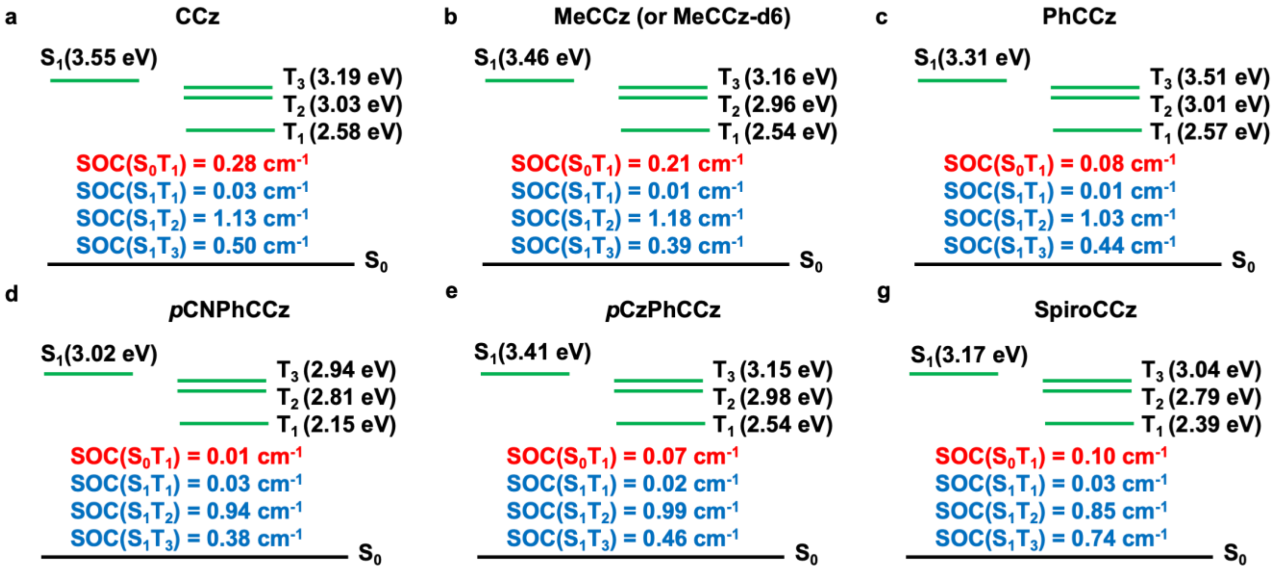

Figure S17. The calculated vertical energy level diagram, spin-orbit couplings (SOC) between singlet and triplet states based on the optimized S_0 geometries in toluene at B3LYP/6-31G(d) level.

V. Photophysical Properties

Fitting of time-resolved luminescence measurements

Time-resolved PL measurements were fitted to a single exponential decay model, with chi-squared (χ^2) values between 0.9 and 1.1, using the FLS980 software.

$$R(t) = B_1 e^{(-t/\tau_1)}$$

The reported parameters (RTP component of PLQY, rate constants of RTP, nonradiative decay, and ISC) in **Table 1**. of CCz emitters were calculated by equations S1-S4. [15-18]

$$\Phi_{RTP} = \Phi_{PL} - \Phi_{Fl} \qquad \text{(Eq S1)}$$

$$k_{RTP} = \Phi_{RTP}/\tau_{RTP} \qquad \text{(Eq S2)}$$

$$k_{nr} = (1 - \Phi_{Fl} - \Phi_{RTP})/\tau_{RTP} \qquad \text{(Eq S3)}$$

$$k_{ISC} = \Phi_{RTP}/\tau_{Fl} \qquad \text{(Eq S4)}$$

Where Φ_{PL} was determined under nitrogen atmosphere and Φ_{Fl} was determined under oxygen atmosphere in the integrating sphere system.

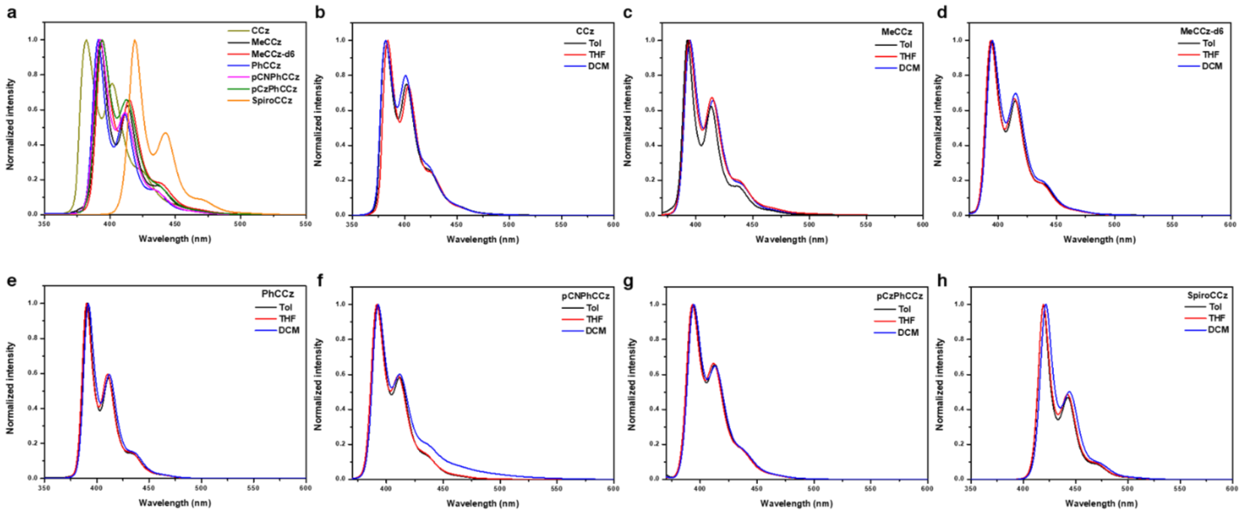

Figure S18. (a) Overlapped steady-state PL spectra for all CCz emitters in toluene solutions (10^{-5} M). (b)-(h) The steady-state PL spectra of all CCz emitters in toluene, tetrahydrofuran and dichloromethane solution (10^{-5} M).

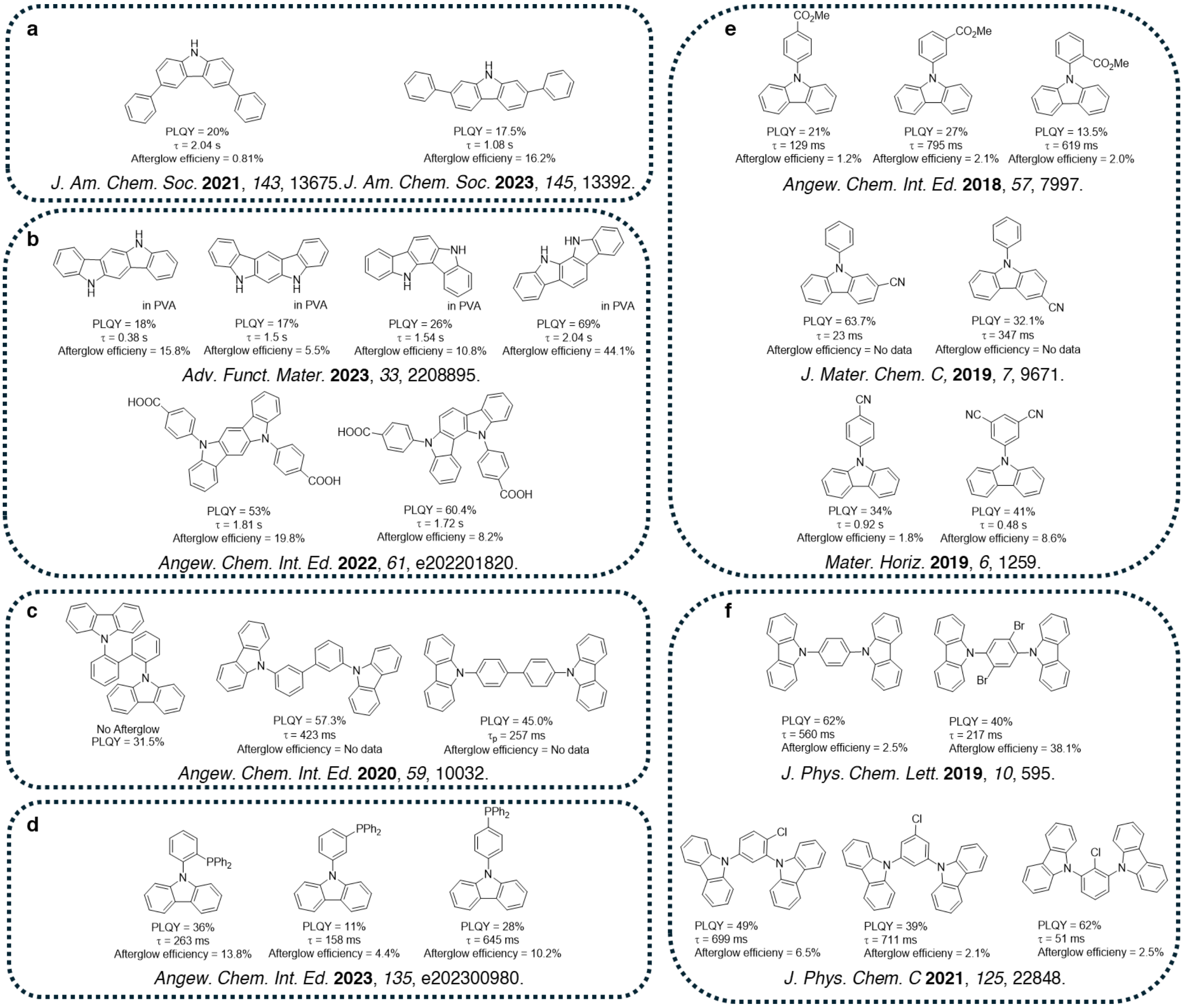

Figure S19. Summary of recent carbazole-based afterglow emitters' PLQYs and lifetimes. (a) Carbazole derivatives. (b) Indolocarbazole isomers. (c) *N*-arylated carbazoles. (d) *N*-arylated carbazoles with electron-donating groups. (e) *N*-arylated carbazoles with electron-withdrawing groups. (f) Carbazole derivatives with heavy atom effect.

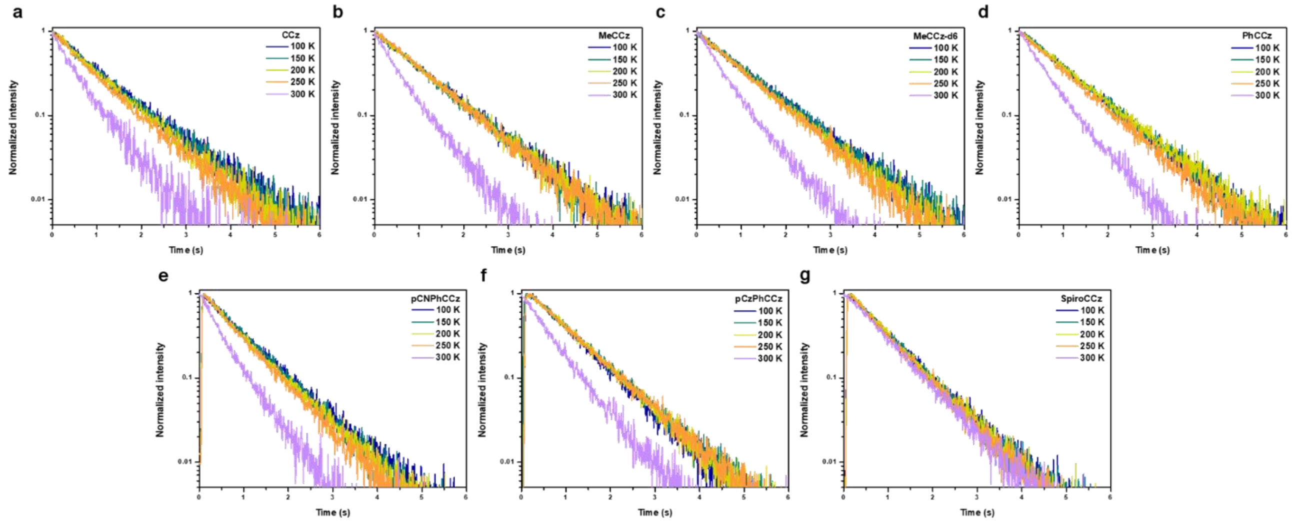

Figure S20. (a)-(g) Temperature-dependence transient PL spectra at RTP regions of the doped films (2 wt% CCz compounds in PMMA).

Table S7. Summary of the RTP and LTP properties of CCz compounds doped in 2 wt% PMMA films.

Compound	τ_{RTP}[a] [ms]	λ_{RTP}[b] [nm]	τ_{LTP}[c] [ms]	λ_{LTP}[d] [nm]
CCz	518	497, 531	1044	489, 528
MeCCz	530	505, 543	1148	499, 537
MeCCz-d6	534	504, 542	1065	500, 536
PhCCz	549	505, 541	1084	499, 537
*p*CNPhCCz	456	513, 548	854	505, 542
*p*CzPhCCz	590	507, 544	985	502, 539
SpiroCCz	719	537, 578	862	533, 576

[a] Lifetimes of the RTP (τ_{RTP}). [b] RTP emission peaks. [c] Lifetimes of the LTP (τ_{LTP}) at 80 K. [d] LTP emission peaks. Abbreviations: LTP (low-temperature time-gated PL) and RTP (room-temperature time-gated PL).

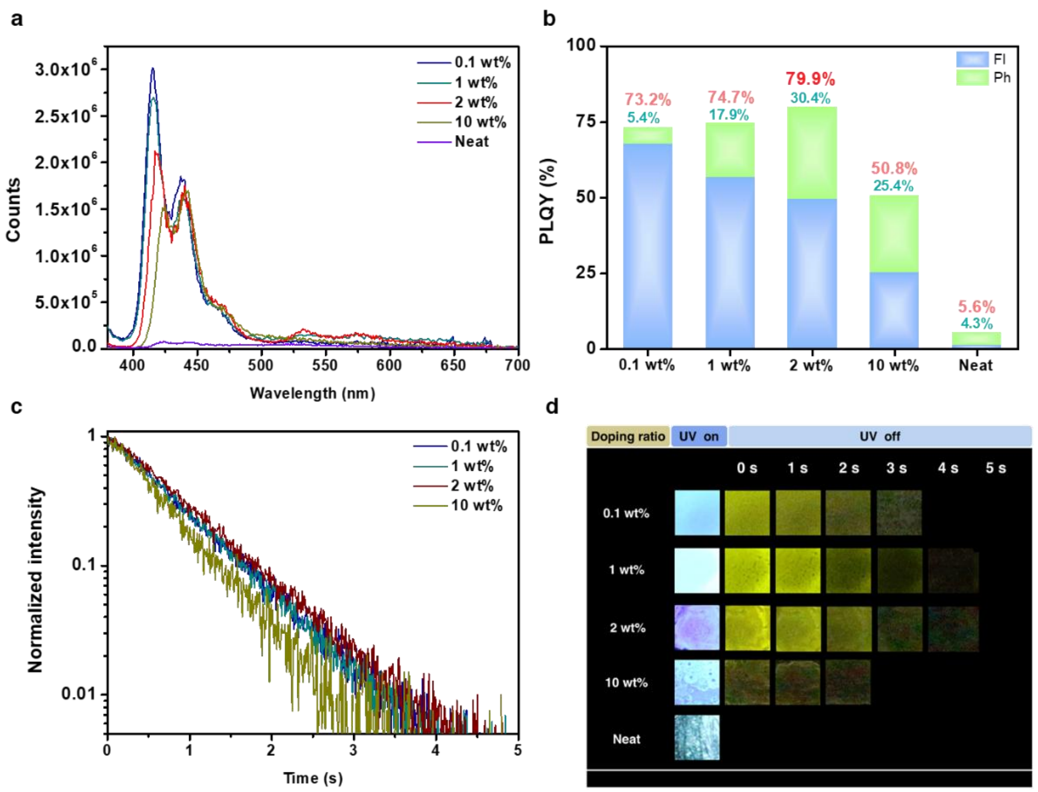

Figure S21. Dopant concentrations of 0.1, 1, 2, and 10 wt% SpiroCCz in PMMA films and a neat SpiroCCz film were used for the (a) steady-state PL spectrum, (b) afterglow decay curves, (c) Φ_{PL} and Φ_{RTP} values at room temperature, and (d) Camera-recorded images displaying RTP afterglows under nitrogen with a 365 nm UV lamp.

Table S8. Summary of the photophysical properties of SpiroCCz in different doping concentrations PMMA films and a neat SpiroCCz film.

Doping ratio	λ_{SSPL}[a] [nm]	λ_{RTP}[a] [nm]	Φ_{PL}[b] [%]	Φ_{RTP}[c] [%]	τ_{RTP}[d] [ms]
0.1	418, 440	536, 577	73.2	5.40	742
1	418, 440	537, 576	74.7	17.9	726
2	419, 440	537, 578	79.9	30.4	719
10	422, 441	540, 577	50.8	25.4	604
Neat	441	-	5.6	4.3	-

[a] Steady-state PL emission peaks and RTP maximum bands. [b] Absolute total PLQY value. [c] RTP component of PLQY. [d] RTP Lifetimes.

VI. Appendix

NMR spectra

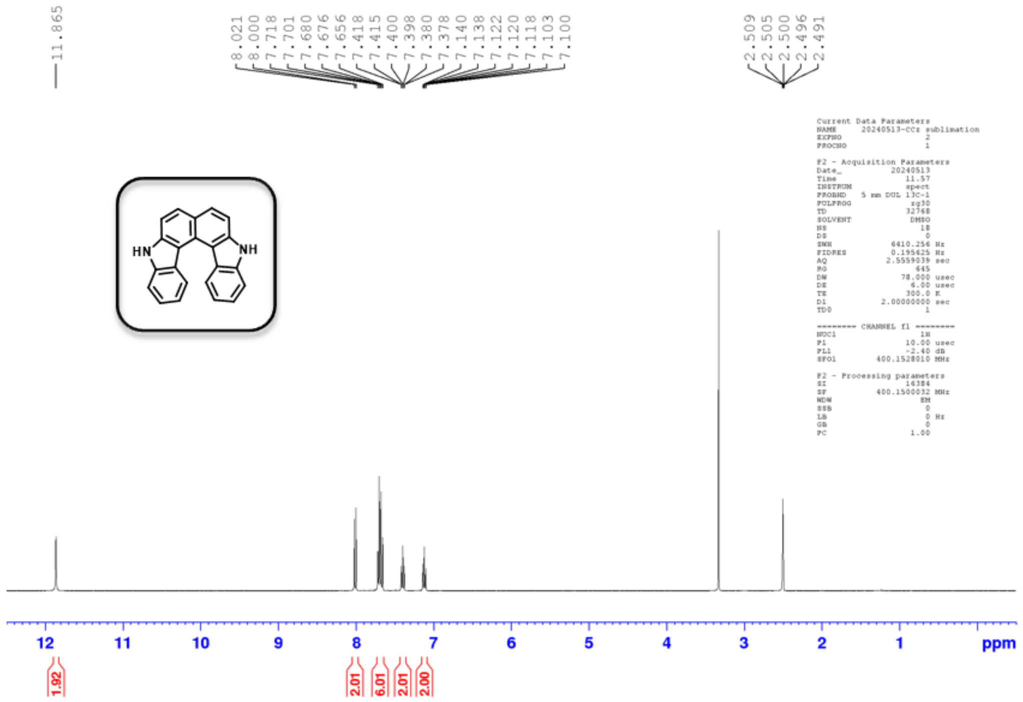

Figure S22. ^{1}H NMR spectrum of CCz.

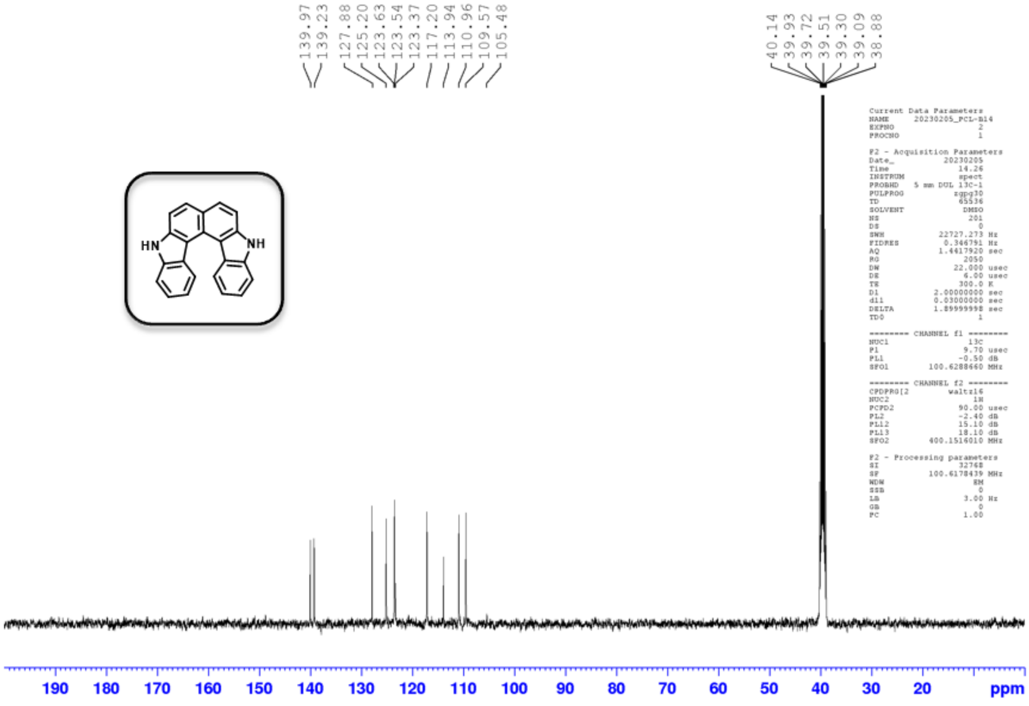

Figure S23. ^{13}C NMR spectrum of CCz.

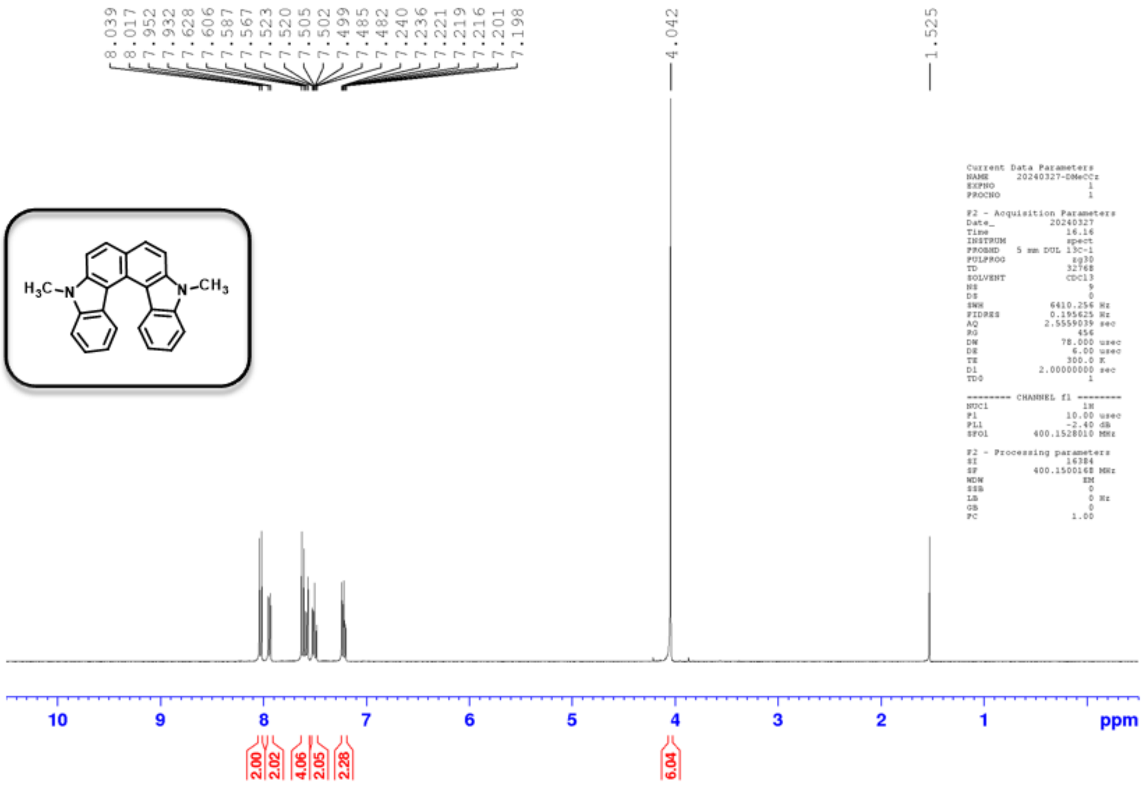

Figure S24. ^{1}H NMR spectrum of MeCCz.

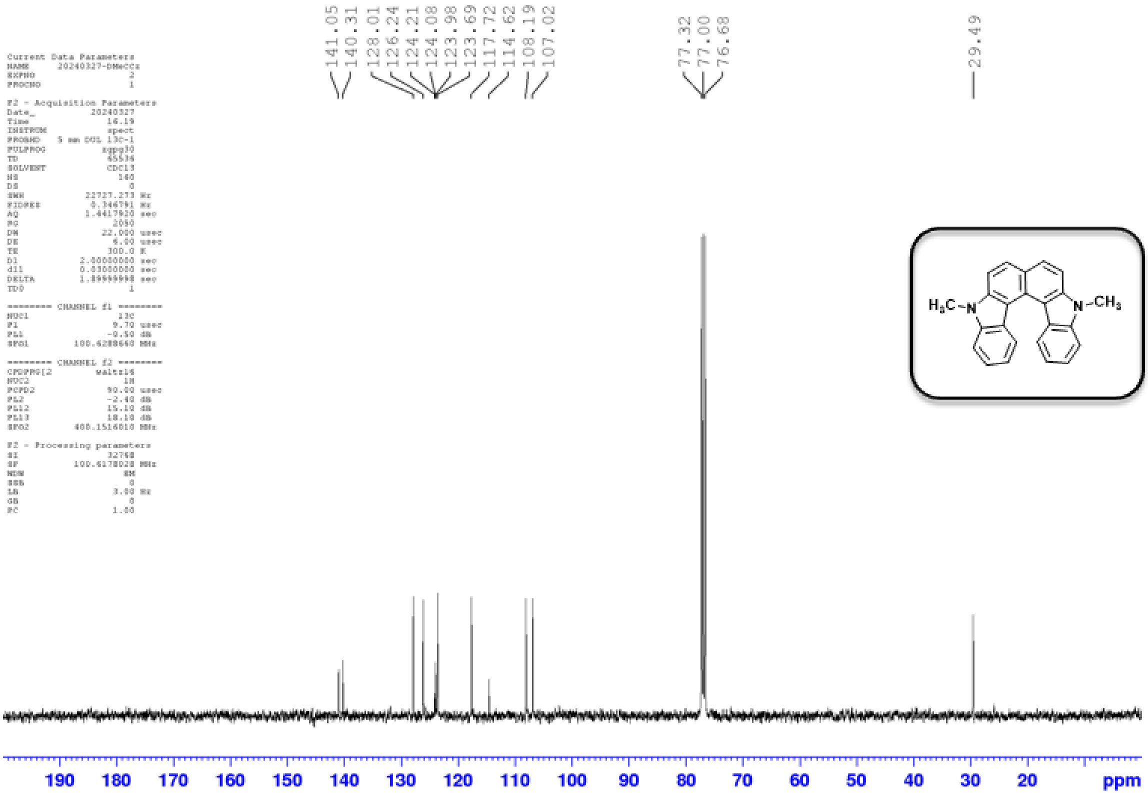

Figure S25. ^{13}C NMR spectrum of MeCCz.

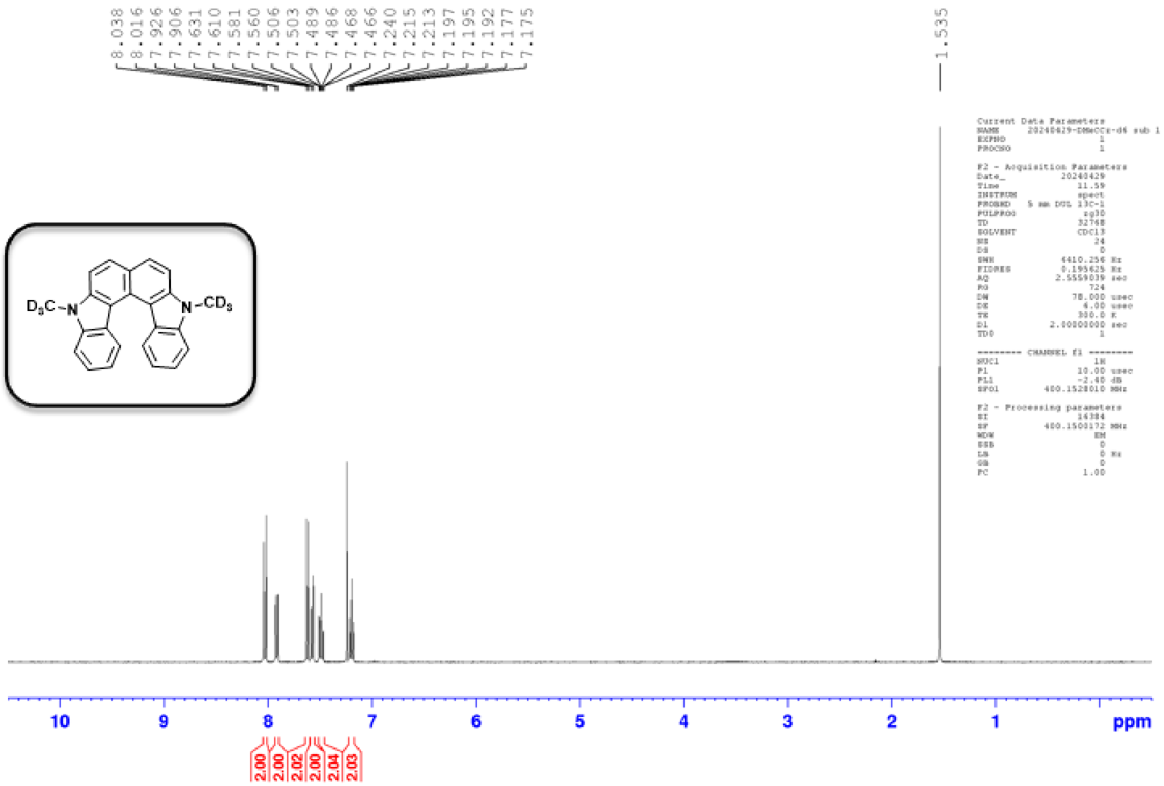

Figure S26. ^{1}H NMR spectrum of MeCCz-d6.

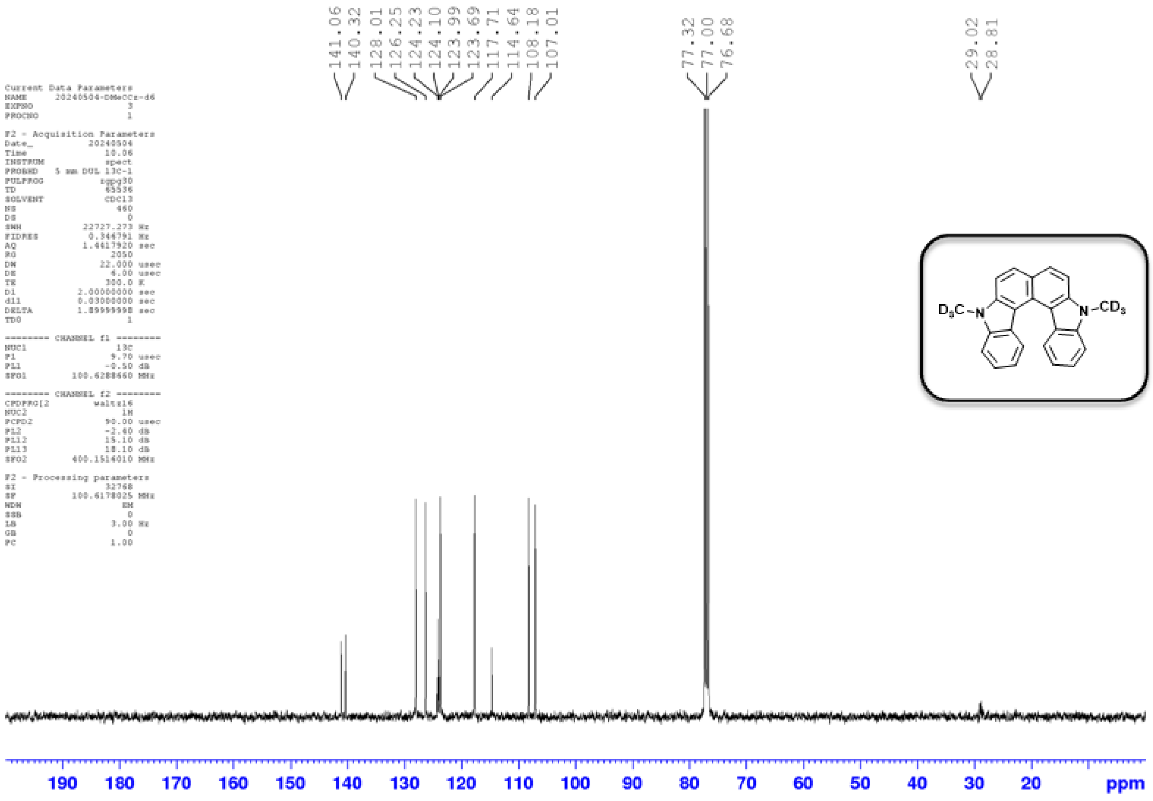

Figure S27. ^{13}C NMR spectrum of MeCCz-d6.

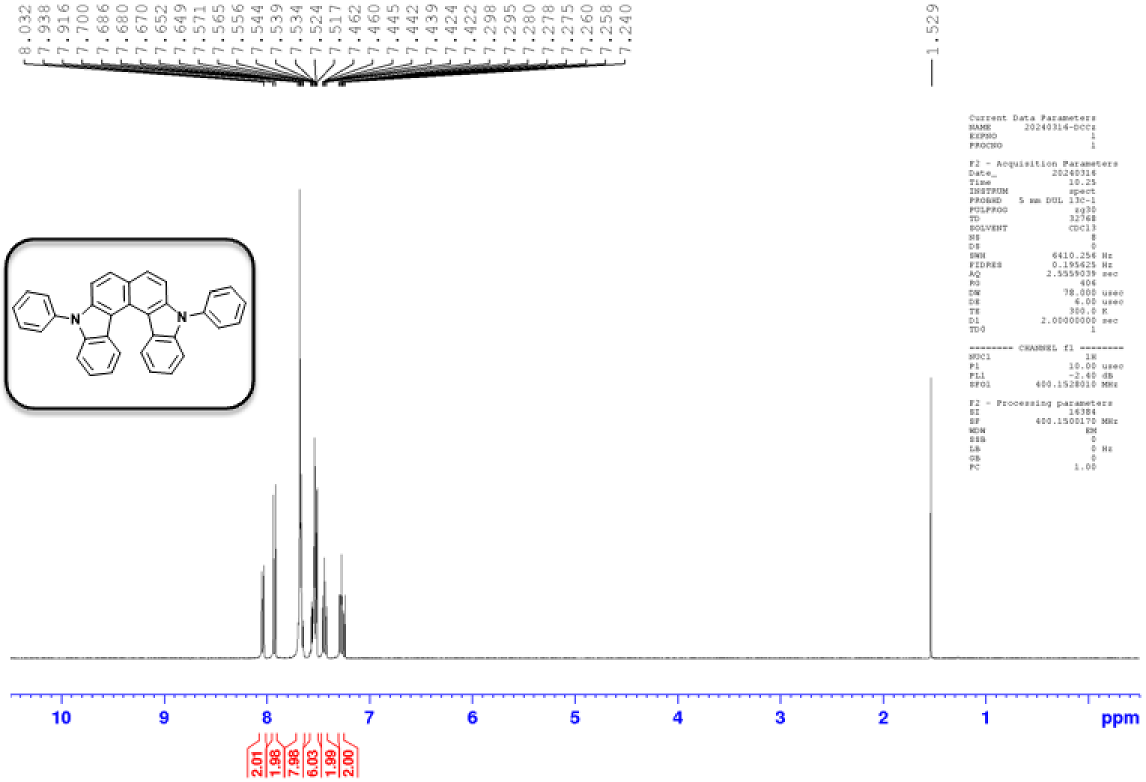

Figure S28. ^{1}H NMR spectrum of PhCCz.

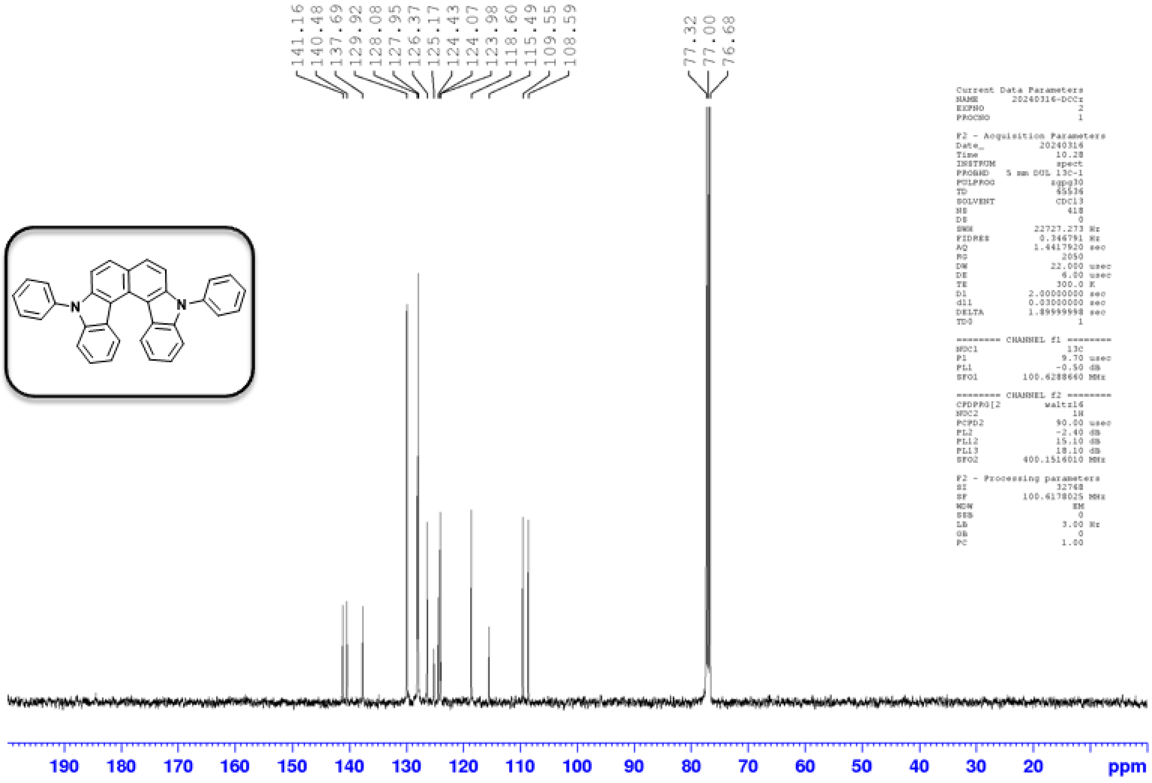

Figure S29. ^{13}C NMR spectrum of PhCCz.

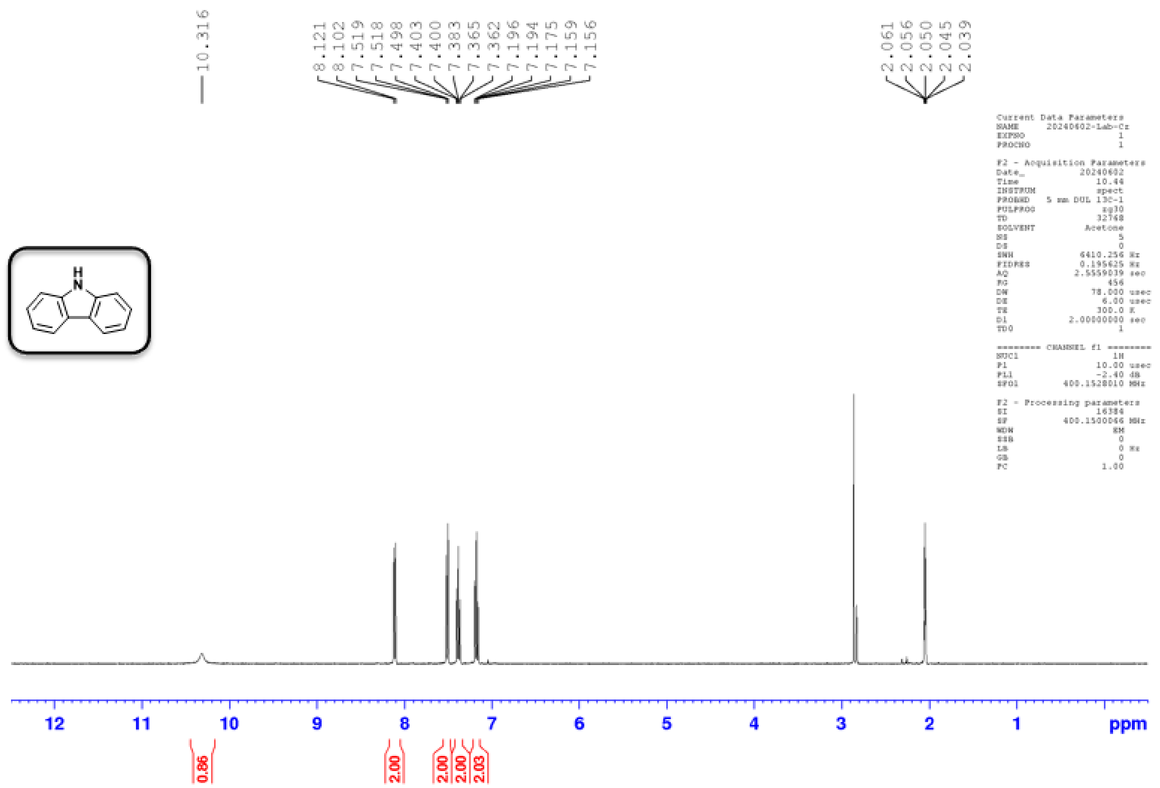

Figure S30. ^{1}H NMR spectrum of 9*H*-carbazole.

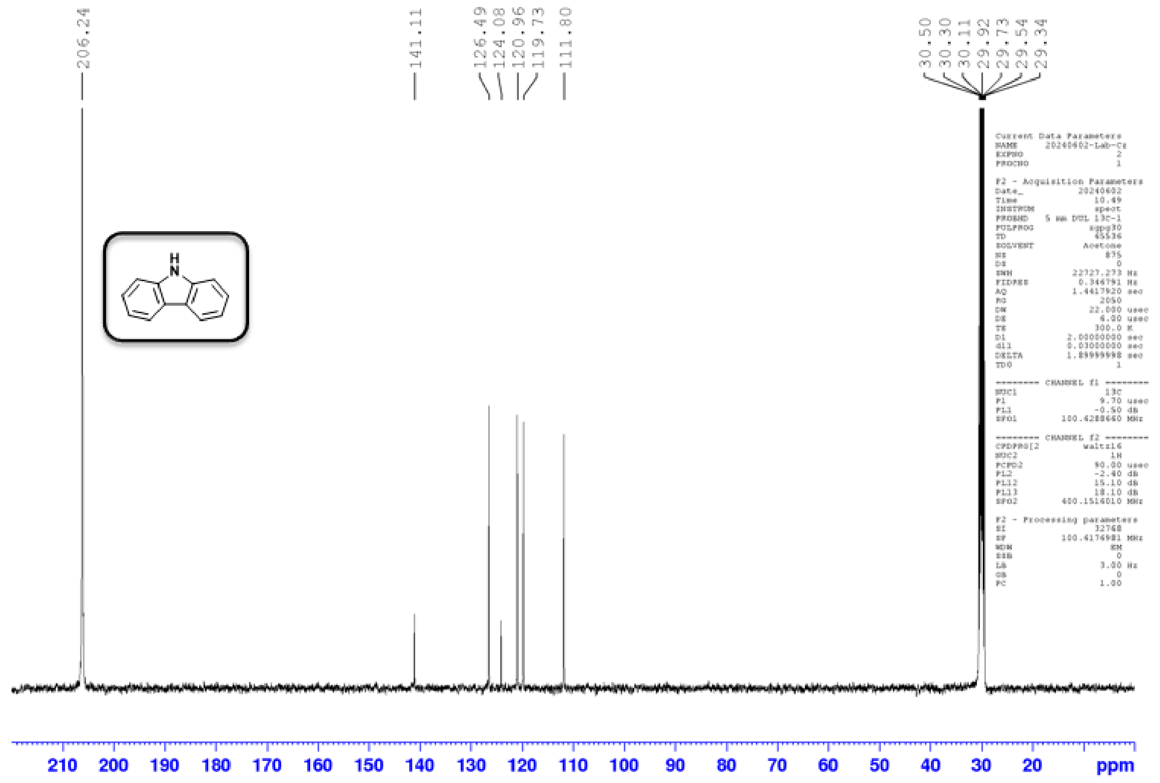

Figure S31. ^{13}C NMR spectrum of 9*H*-carbazole.

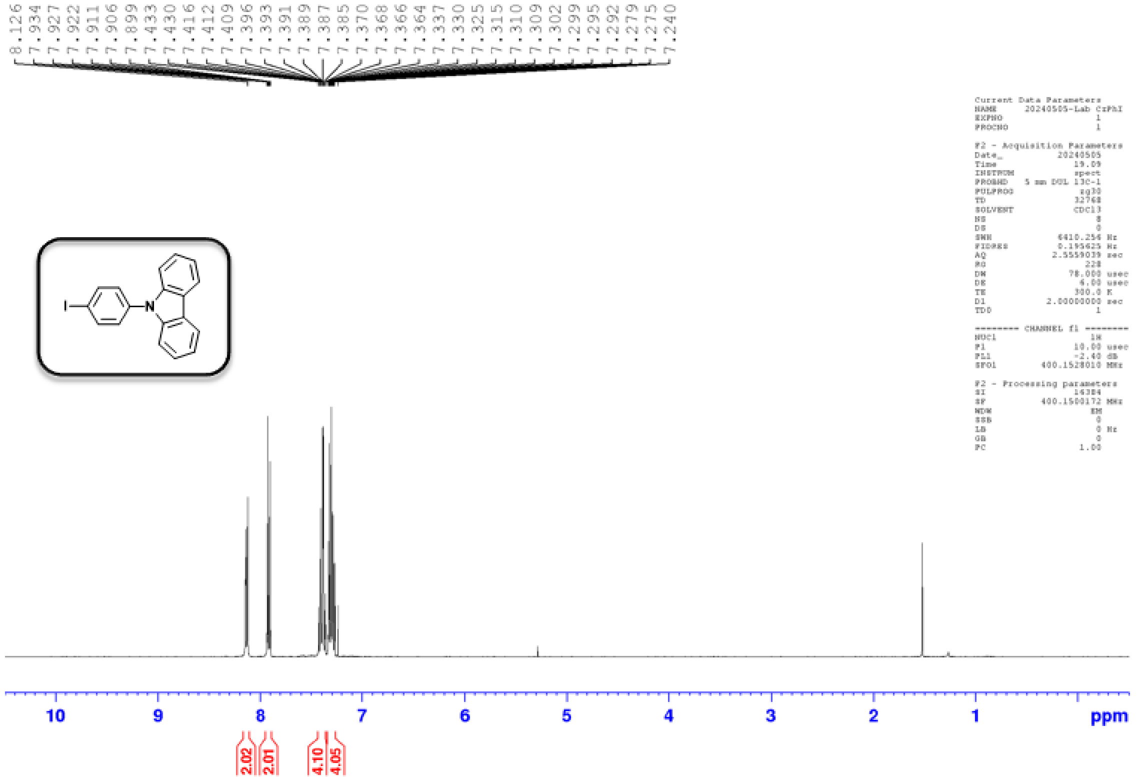

Figure S32. ^{1}H NMR spectrum of 9-(4-iodophenyl)-9*H*-carbazole.

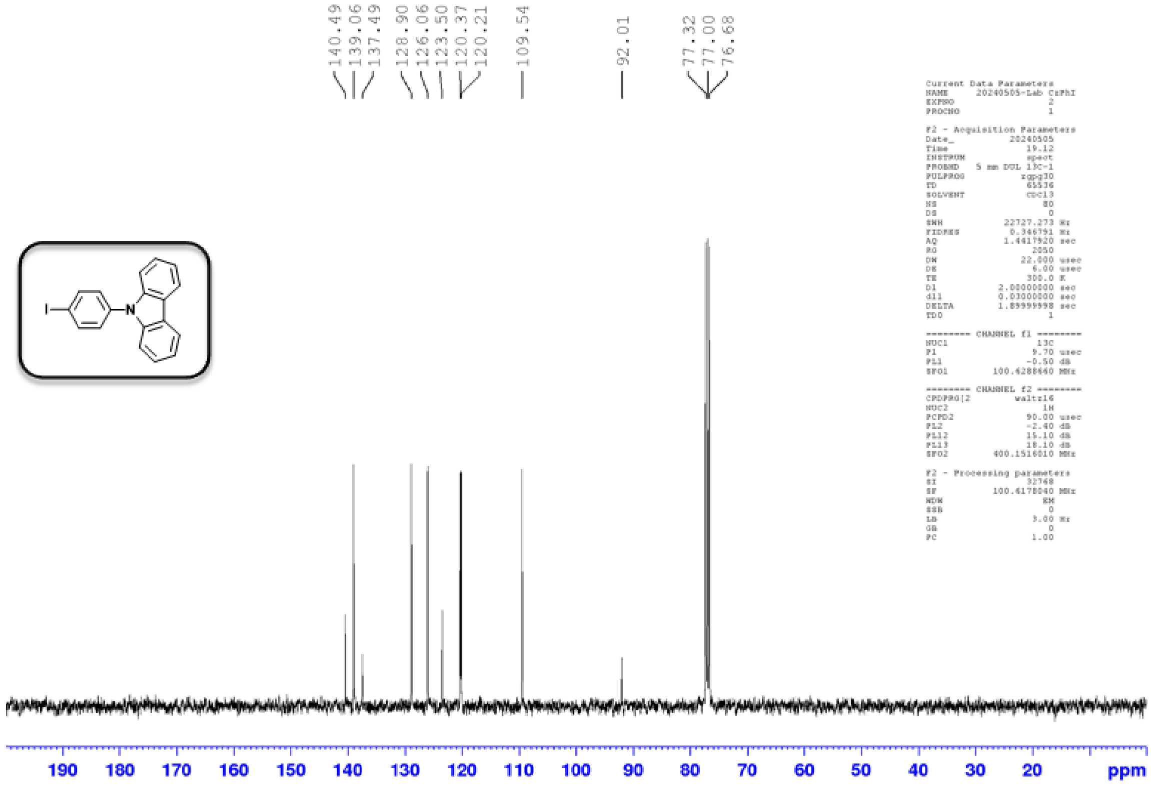

Figure S33. ^{13}C NMR spectrum of 9-(4-iodophenyl)-9*H*-carbazole.

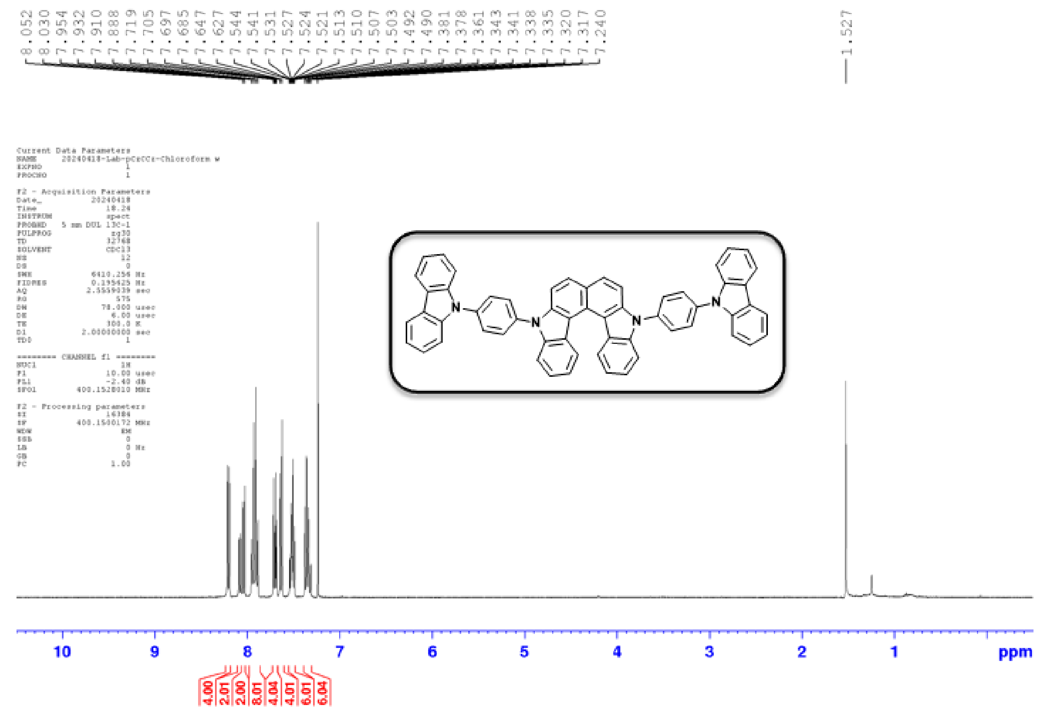

Figure S34. ^{1}H NMR spectrum of *p*CzPhCCz.

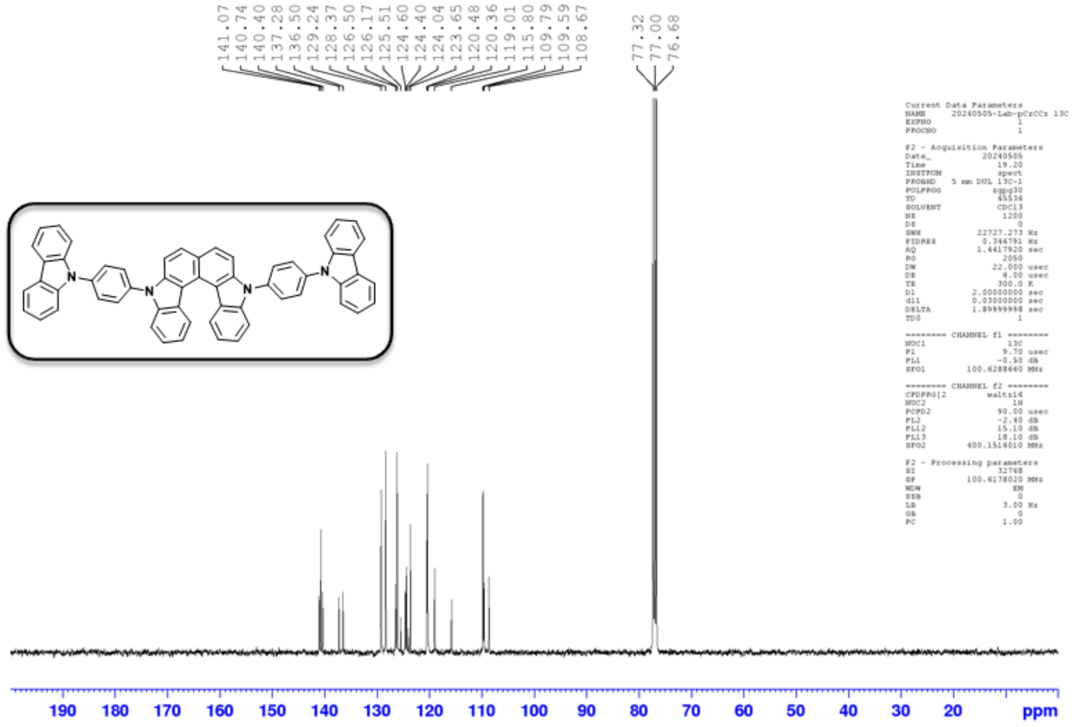

Figure S35. ^{13}C NMR spectrum of *p*CzPhCCz.

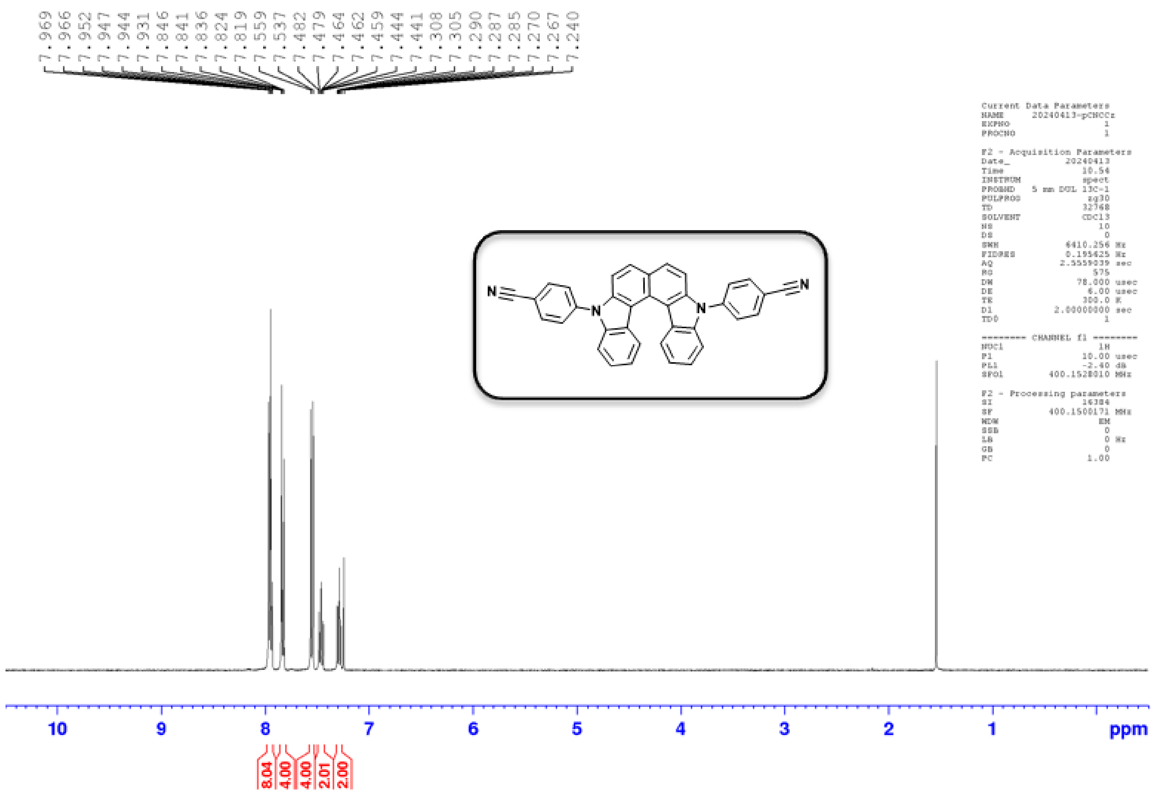

Figure S36. ^{1}H NMR spectrum of *p*CNPhCCz.

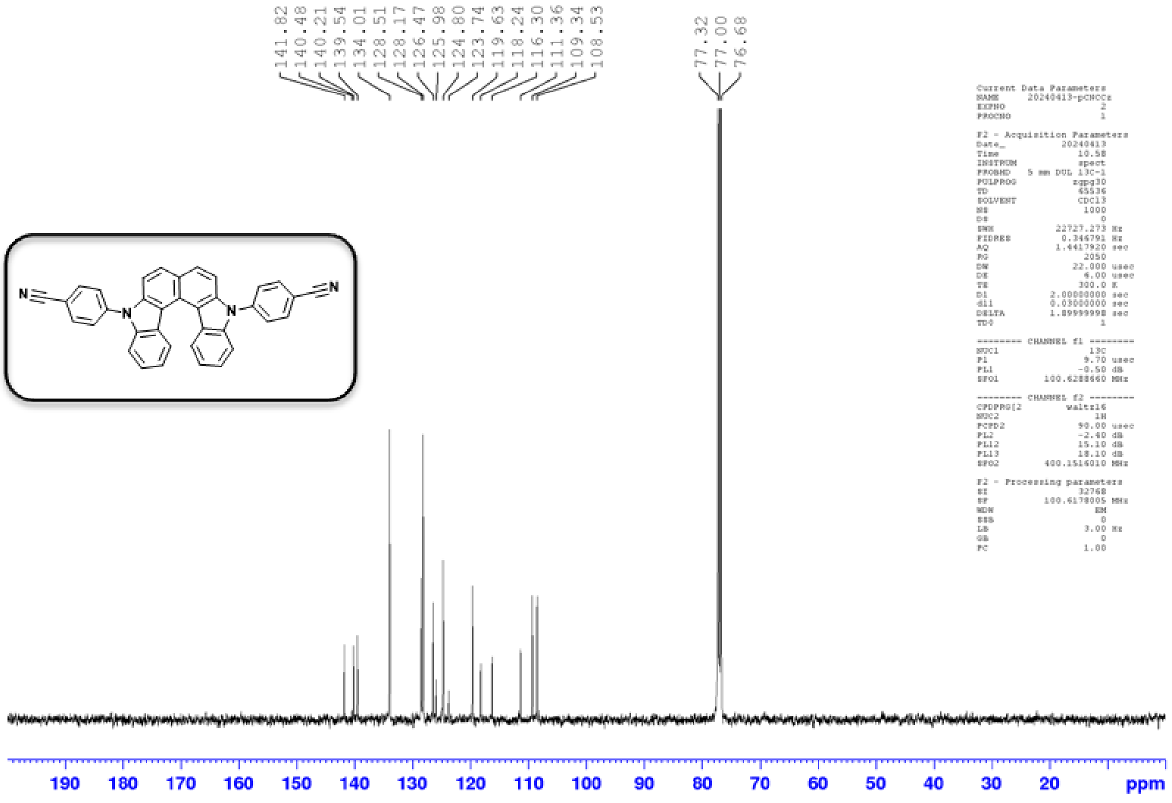

Figure S37. ^{13}C NMR spectrum of *p*CNPhCCz.

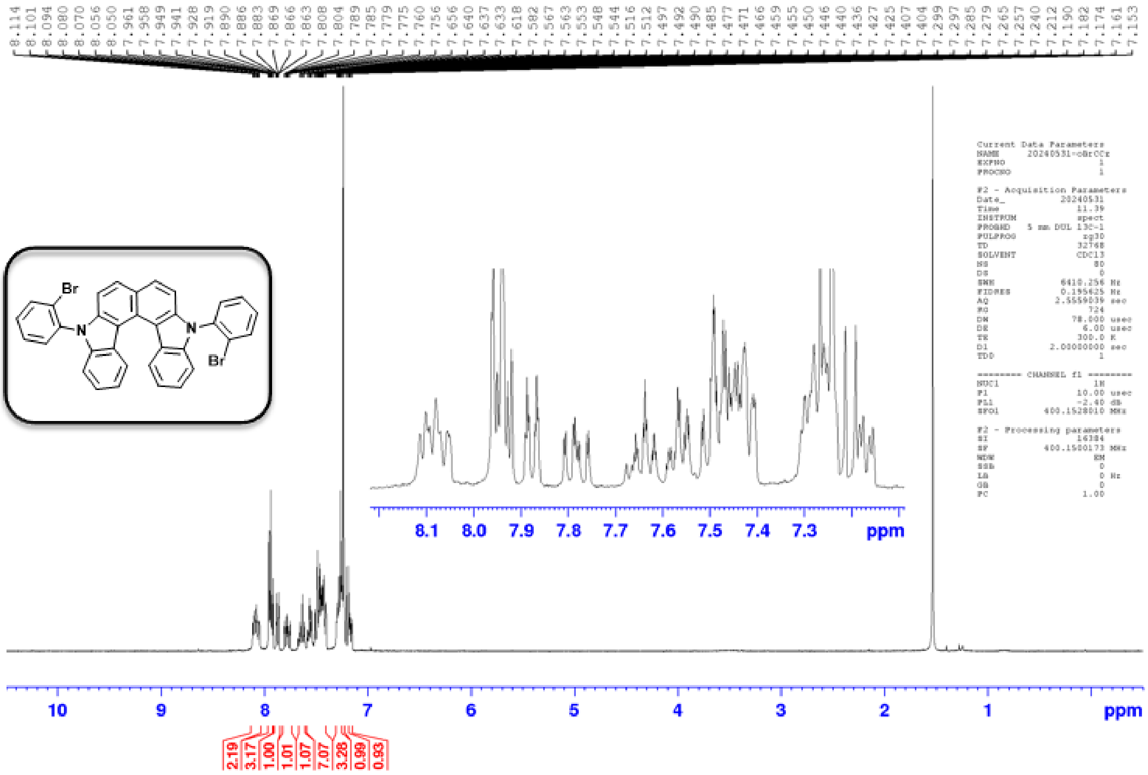

Figure S38. ^{1}H NMR spectrum of 5,10-bis(2-bromophenyl)-5,10-dihydrocarbazolo[3,4-*c*]carbazole.

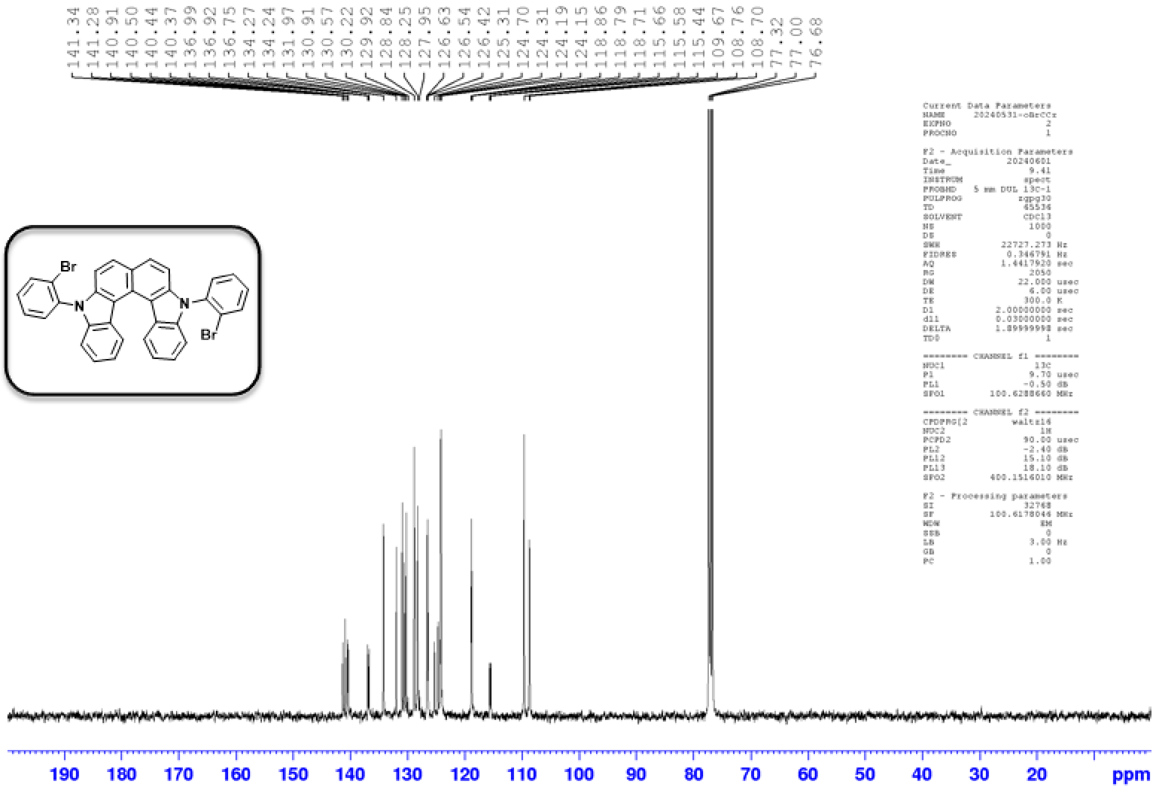

Figure S39. ^{13}C NMR spectrum of 5,10-bis(2-bromophenyl)-5,10-dihydrocarbazolo[3,4-*c*]carbazole.

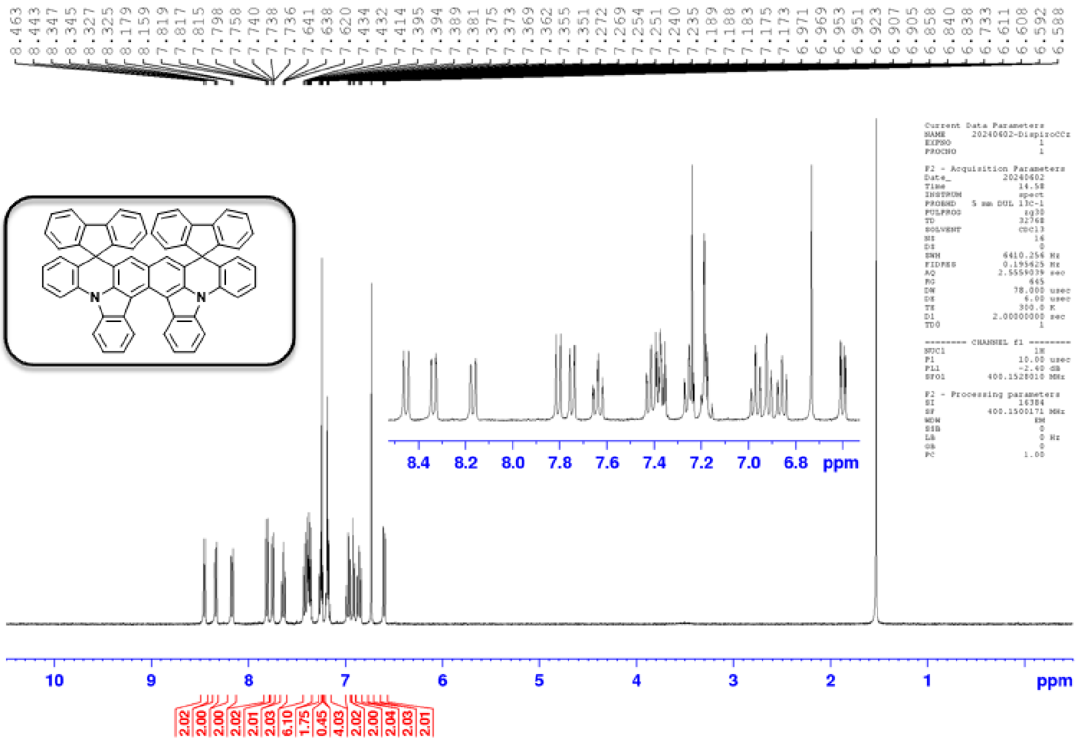

Figure S40. ^{1}H NMR spectrum of SpiroCCz.

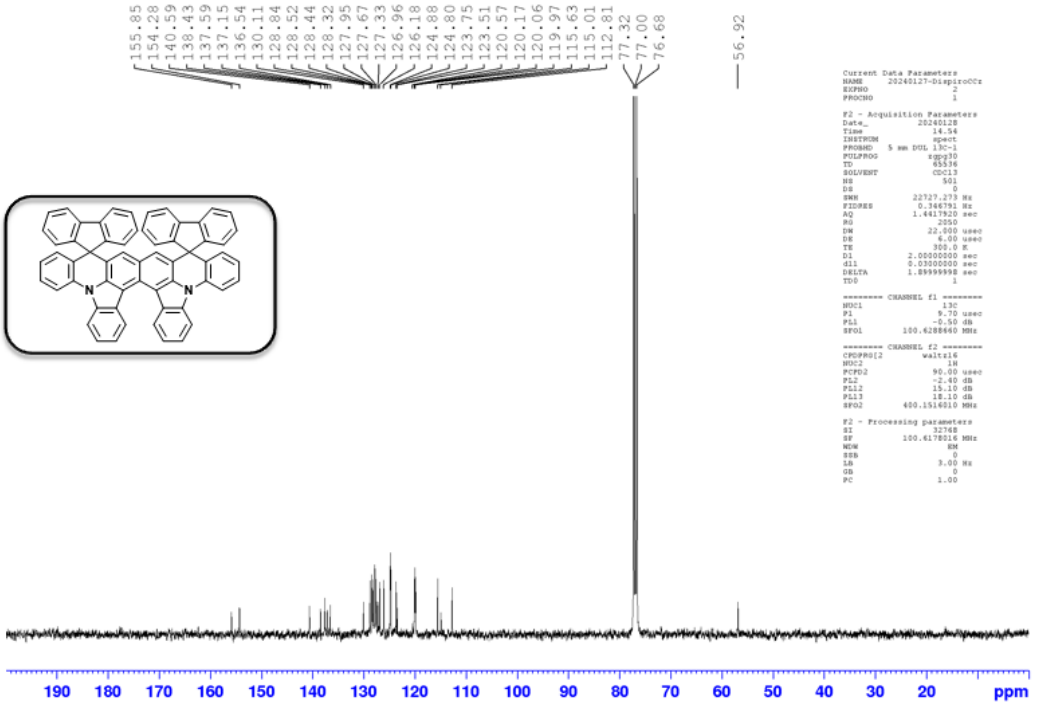

Figure S41. ^{13}C NMR spectrum of SpiroCCz.

HRMS spectra

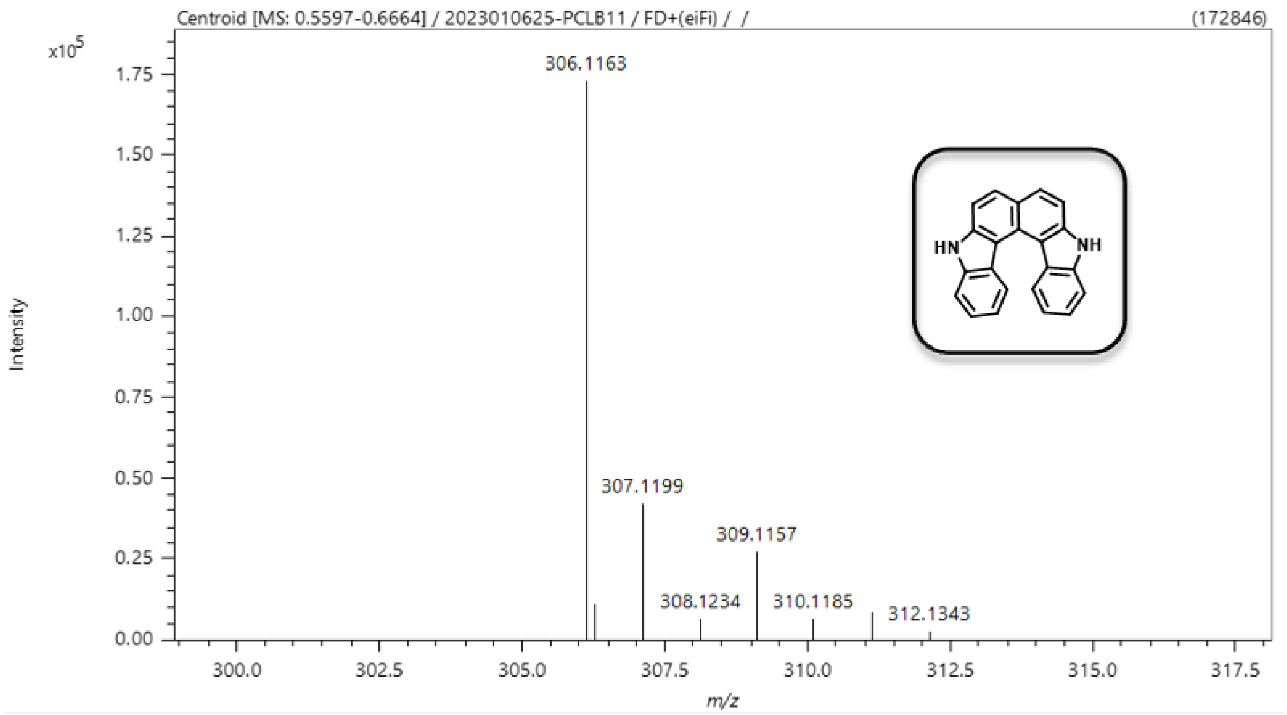

Figure S42. FD-HRMS of CCz.

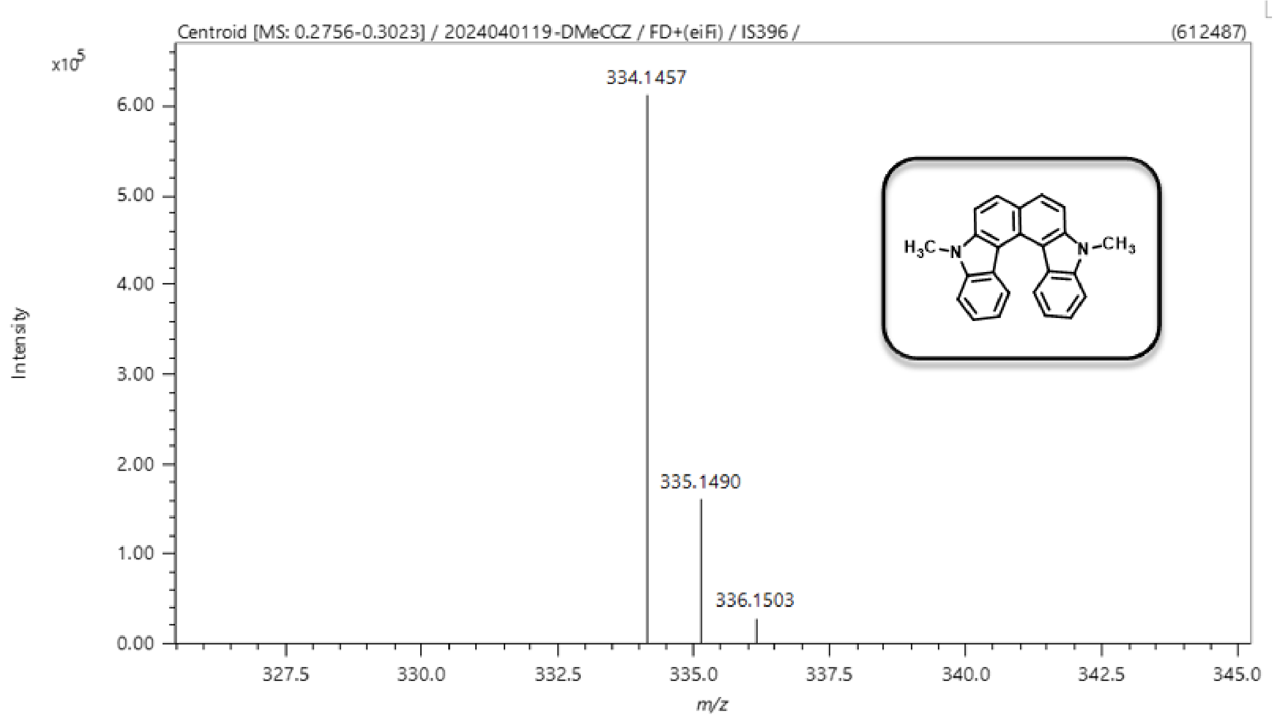

Figure S43. FD-HRMS of MeCCz.

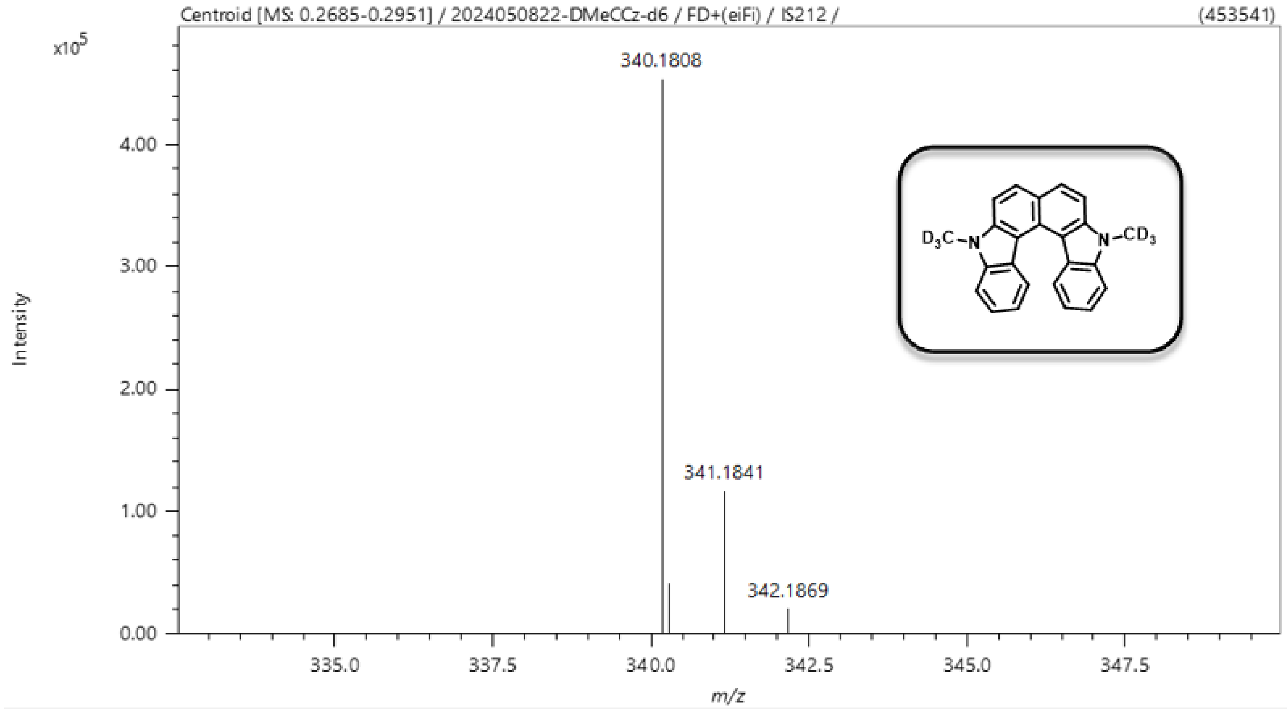

Figure S44. FD-HRMS of MeCCz-d6.

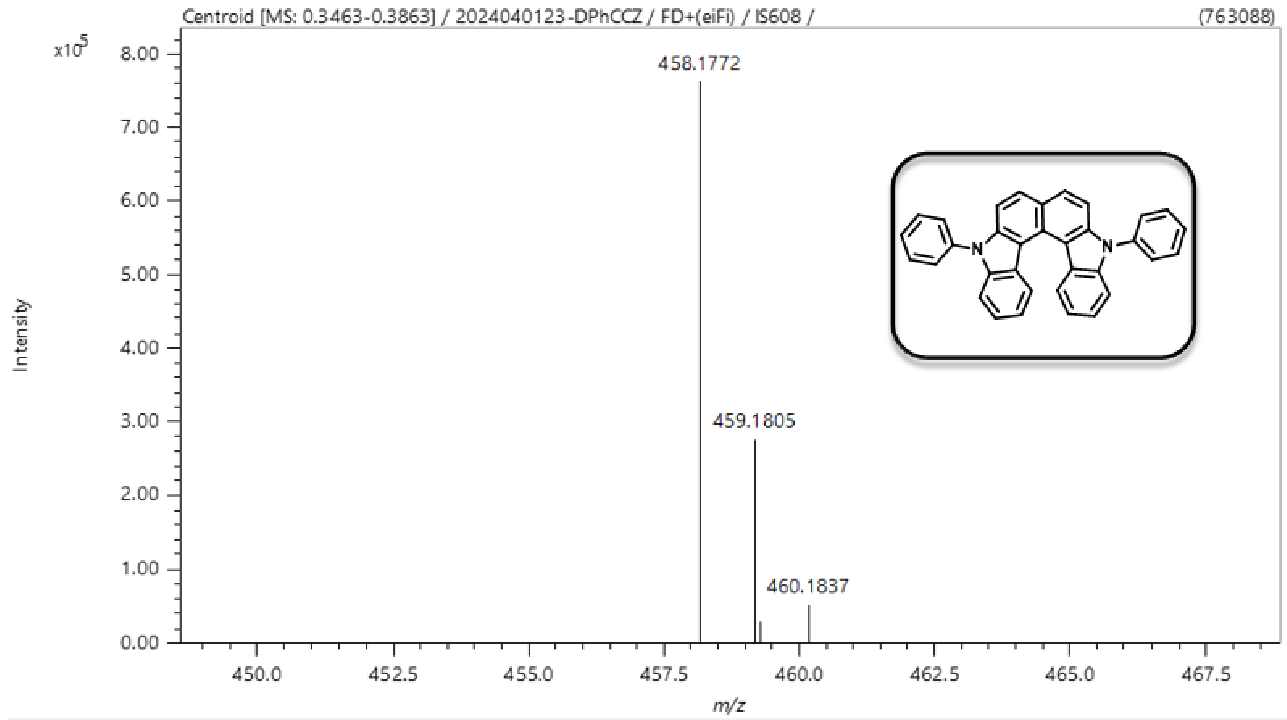

Figure S45. FD-HRMS of PhCCz.

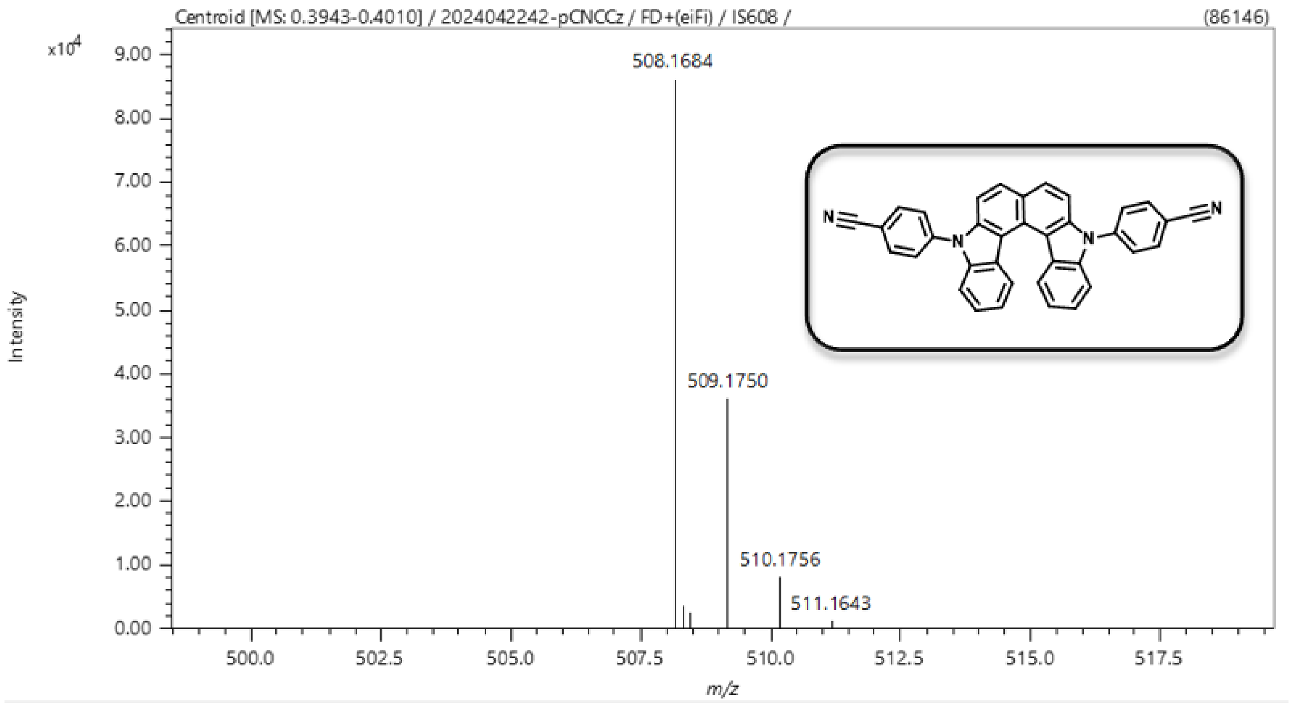

Figure S46. FD-HRMS of *p*CNPhCCz.

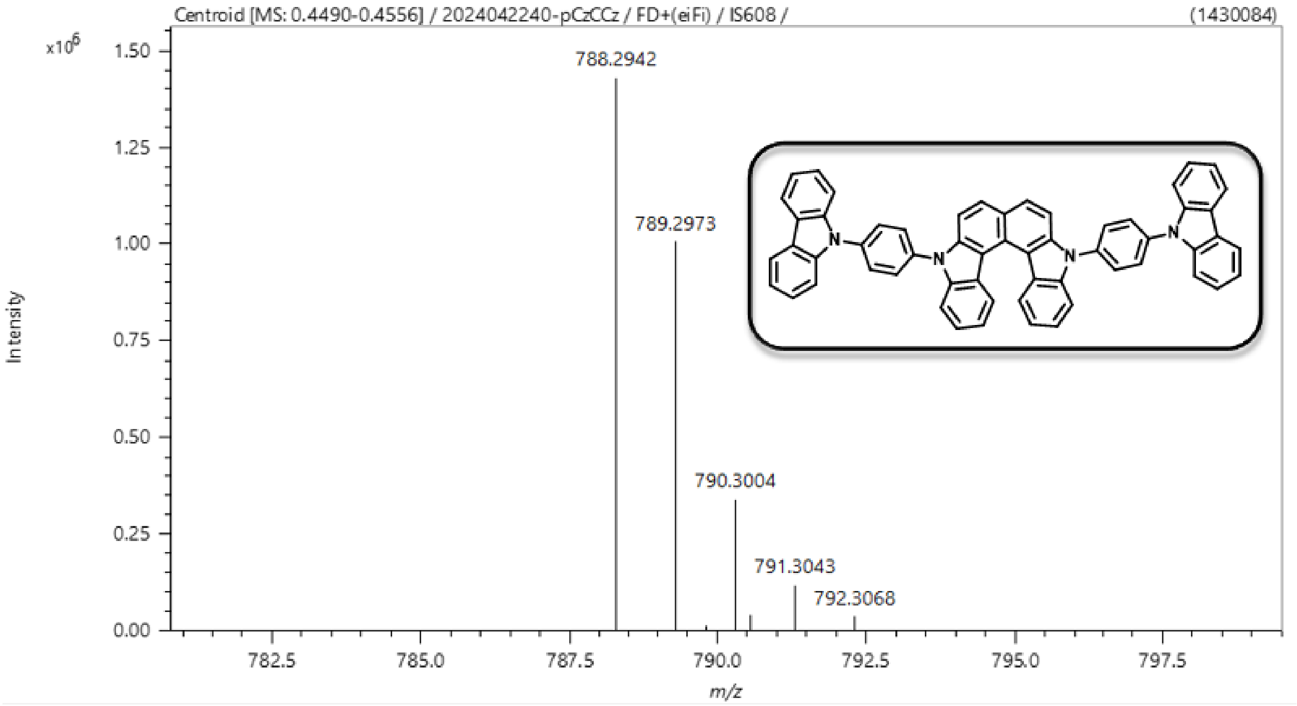

Figure S47. FD-HRMS of *p*CzPhCCz.

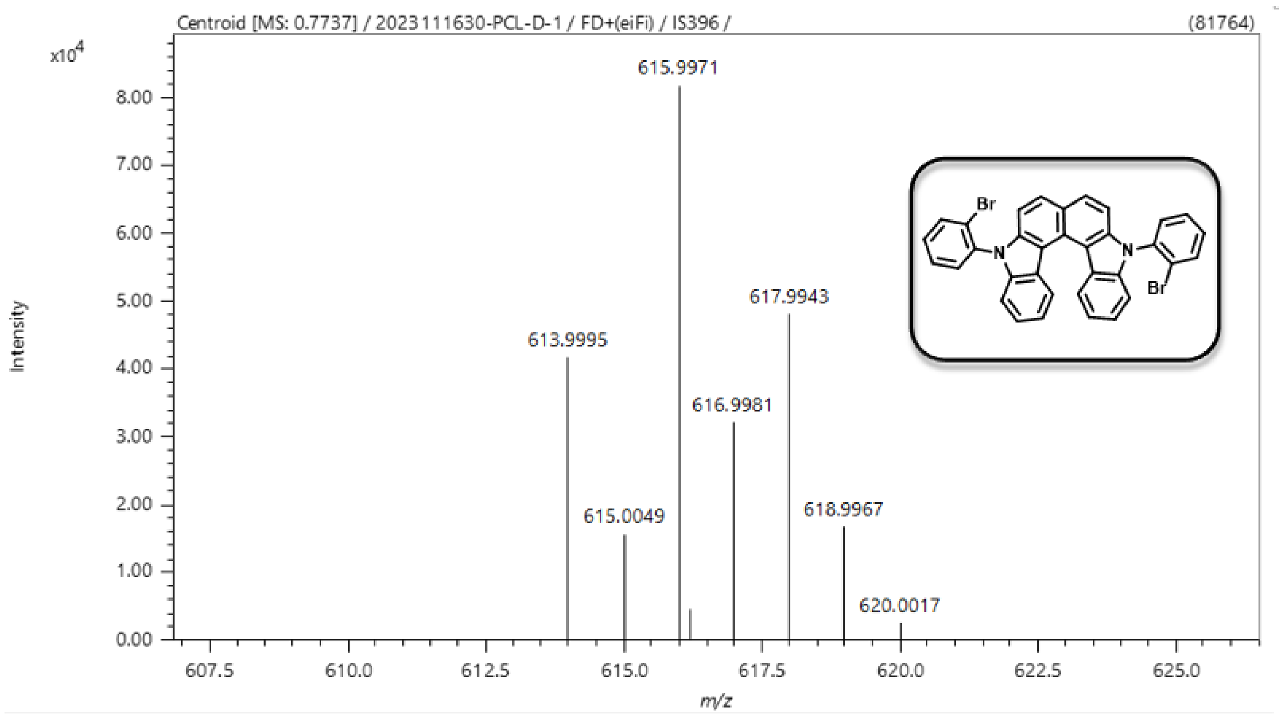

Figure S48. FD-HRMS of 5,10-bis(2-bromophenyl)-5,10-dihydrocarbazolo[3,4-*c*]carbazole.

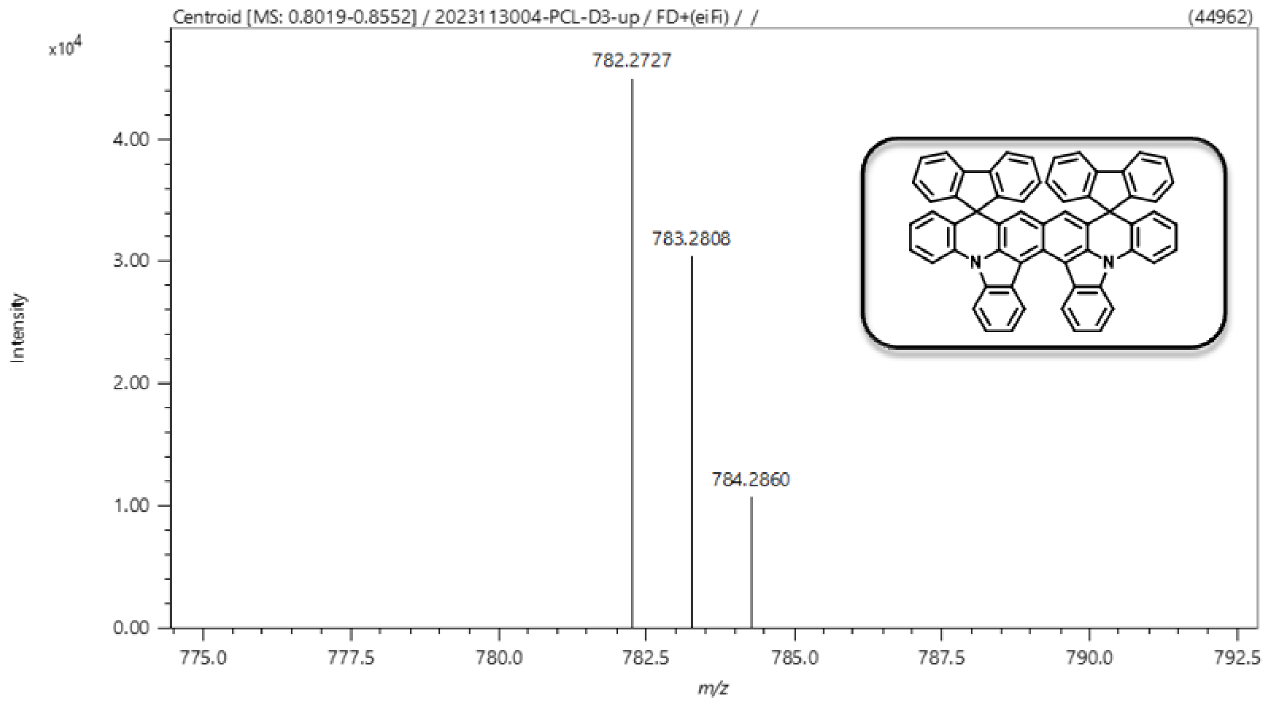

Figure S49. FD-HRMS of SpiroCCz.

Cartesian coordinates of optimized structures

Table S9. Cartesian coordinates of optimized S_0, S_1, and T_1 states of CCz.

Elements	X	Y	Z	X	Y	Z	X	Y	Z
		S_0			S_1			T_1	
C	-1.0244	2.1587	-2.9082	-2.0661	-2.9099	1.1125	-2.0689	-2.8769	1.1512
C	-0.6049	3.5003	-2.8816	-3.4029	-2.9136	0.7130	-3.4089	-2.8860	0.7413
C	0.0000	4.0344	-1.7474	-3.9772	-1.7823	0.1011	-3.9777	-1.7743	0.1030
C	0.1897	3.1878	-0.6532	-3.1595	-0.6794	-0.1011	-3.1641	-0.6704	-0.1210
C	-0.1635	1.8078	-0.6742	-1.7756	-0.6635	0.2422	-1.7804	-0.6507	0.2379
C	-0.8161	1.3166	-1.8190	-1.2438	-1.7949	0.8874	-1.2521	-1.7717	0.9094
H	-1.5288	1.7732	-3.7899	-1.6509	-3.7821	1.6095	-1.6599	-3.7393	1.6696
H	-0.7746	4.1355	-3.7467	-4.0191	-3.7909	0.8893	-4.0254	-3.7596	0.9344
H	0.2915	5.0804	-1.7021	-5.0265	-1.7664	-0.1792	-5.0248	-1.7681	-0.1861
H	-1.1748	0.2955	-1.8520	-0.2168	-1.8024	1.2288	-0.2259	-1.7699	1.2545
C	0.5599	2.3847	1.4204	-2.4321	1.4328	-0.5163	-2.4297	1.4324	-0.5429
C	0.7231	2.3390	2.8189	-2.3940	2.8052	-0.7044	-2.3898	2.7924	-0.7016
C	0.3829	1.1679	3.4512	-1.1898	3.4535	-0.4020	-1.1731	3.4515	-0.3754
C	0.0000	0.0000	2.7291	-0.0112	2.7464	-0.0191	0.0000	2.7464	0.0000
C	0.0000	0.0000	1.2865	-0.0068	1.2925	-0.0089	0.0000	1.2956	0.0000
C	0.1406	1.2758	0.6479	-1.2705	0.6526	-0.0915	-1.2713	0.6547	-0.1026
H	1.0374	3.2170	3.3758	-3.2713	3.3599	-1.0227	-3.2618	3.3583	-1.0155
H	0.4150	1.1100	4.5364	-1.1299	4.5355	-0.4827	-1.1239	4.5346	-0.4399
C	-0.1406	-1.2758	0.6479	1.2718	0.6534	0.1057	1.2713	0.6547	0.1026
C	-0.7231	-2.3390	2.8189	2.3646	2.8016	0.7081	2.3898	2.7924	0.7016
H	-1.0374	-3.2170	3.3758	3.2312	3.3747	1.0240	3.2618	3.3583	1.0155
C	-0.5599	-2.3847	1.4204	2.4175	1.4347	0.5517	2.4297	1.4324	0.5429
C	0.1635	-1.8078	-0.6742	1.7844	-0.6405	-0.2397	1.7804	-0.6507	-0.2379
C	-0.1897	-3.1878	-0.6532	3.1718	-0.6561	0.1235	3.1641	-0.6704	0.1210
C	0.6049	-3.5003	-2.8816	3.4252	-2.8737	-0.7290	3.4089	-2.8860	-0.7413
H	0.7746	-4.1355	-3.7467	4.0435	-3.7472	-0.9169	4.0254	-3.7596	-0.9344
C	1.0244	-2.1587	-2.9082	2.0846	-2.8693	-1.1455	2.0689	-2.8769	-1.1512
H	1.5288	-1.7732	-3.7899	1.6832	-3.7359	-1.6631	1.6599	-3.7393	-1.6696
C	0.0000	-4.0344	-1.7474	3.9912	-1.7586	-0.0928	3.9777	-1.7743	-0.1030
H	-0.2915	-5.0804	-1.7021	5.0374	-1.7482	0.1989	5.0248	-1.7681	0.1861
C	0.8161	-1.3166	-1.8190	1.2608	-1.7683	-0.9134	1.2521	-1.7717	-0.9094
H	1.1748	-0.2955	-1.8520	0.2377	-1.7686	-1.2666	0.2259	-1.7699	-1.2545

N	0.6632	3.4967	0.6087	-3.5017	0.5780	-0.5969	-3.5061	0.5620	-0.6644
N	-0.6632	-3.4967	0.6087	3.5029	0.5802	0.6570	3.5061	0.5620	0.6645
C	-0.3829	-1.1679	3.4512	1.1526	3.4481	0.3719	1.1731	3.4515	0.3754
H	-0.4150	-1.1100	4.5364	1.0989	4.5312	0.4415	1.1239	4.5346	0.4399
H	-0.8366	-4.4334	0.9423	4.4530	0.8960	0.7890	4.4612	0.8782	0.7516
H	0.8366	4.4334	0.9423	-4.4486	0.8721	-0.7903	-4.4612	0.8782	-0.7517

All calculations are at B3LYP/6-31G(d) in toluene.

Table S10. Cartesian coordinates of optimized S_0, S_1, and T_1 states of MeCCz.

Elements	X	Y	Z	X	Y	Z	X	Y	Z
		S_0			S_1			T_1	
C	2.0551	-3.0032	-1.1947	-1.9860	-3.0026	1.2336	-1.9853	-2.9711	1.2716
C	3.4229	-2.9853	-0.8734	-3.3427	-3.0086	0.9201	-3.3456	-2.9839	0.9400
C	4.0086	-1.8559	-0.3076	-3.9559	-1.8751	0.3480	-3.9575	-1.8722	0.3412
C	3.1872	-0.7548	-0.0475	-3.1523	-0.7720	0.0896	-3.1645	-0.7631	0.0652
C	1.7852	-0.7707	-0.3022	-1.7523	-0.7564	0.3485	-1.7617	-0.7428	0.3455
C	1.2394	-1.9084	-0.9215	-1.1797	-1.8832	0.9571	-1.1904	-1.8603	0.9844
H	1.6279	-3.8800	-1.6734	-1.5373	-3.8736	1.7031	-1.5429	-3.8319	1.7646
H	4.0398	-3.8525	-1.0933	-3.9470	-3.8851	1.1365	-3.9478	-3.8591	1.1687
H	5.0750	-1.8258	-0.1059	-5.0224	-1.8641	0.1475	-5.0219	-1.8760	0.1295
H	0.1945	-1.9310	-1.2044	-0.1333	-1.8896	1.2332	-0.1459	-1.8523	1.2691
C	2.4240	1.3149	0.3837	-2.4573	1.3387	-0.3642	-2.4605	1.3347	-0.3960
C	2.3874	2.7158	0.5453	-2.4326	2.7180	-0.5529	-2.4291	2.6976	-0.5503
C	1.1937	3.3484	0.2942	-1.2164	3.3666	-0.3327	-1.1943	3.3555	-0.3026
C	0.0000	2.6289	0.0000	-0.0131	2.6580	-0.0273	0.0000	2.6520	0.0000
C	0.0000	1.1870	0.0000	-0.0082	1.2005	-0.0178	0.0000	1.2016	0.0000
C	1.2826	0.5488	0.0446	-1.2720	0.5653	-0.0146	-1.2752	0.5611	-0.0246
H	3.2818	3.2822	0.7833	-3.3277	3.2781	-0.8013	-3.3164	3.2710	-0.7974
H	1.1412	4.4339	0.3277	-1.1630	4.4491	-0.4097	-1.1504	4.4388	-0.3681
C	-1.2826	0.5488	-0.0446	1.2762	0.5577	0.0256	1.2752	0.5611	0.0247
C	-2.3874	2.7158	-0.5453	2.3995	2.6988	0.5575	2.4291	2.6976	0.5503
H	-3.2818	3.2822	-0.7833	3.2828	3.2779	0.8068	3.3165	3.2710	0.7974
C	-2.4240	1.3149	-0.3837	2.4424	1.3293	0.4075	2.4605	1.3347	0.3960
C	-1.7852	-0.7707	0.3022	1.7630	-0.7323	-0.3512	1.7617	-0.7428	-0.3455
C	-3.1872	-0.7548	0.0475	3.1723	-0.7511	-0.0645	3.1645	-0.7631	-0.0653

C	-3.4229	-2.9853	0.8734	3.3611	-2.9732	-0.9319	3.3455	-2.9839	-0.9400
H	-4.0398	-3.8525	1.0933	3.9626	-3.8496	-1.1575	3.9477	-3.8591	-1.1686
C	-2.0551	-3.0032	1.1947	1.9970	-2.9612	-1.2728	1.9853	-2.9711	-1.2716
H	-1.6279	-3.8800	1.6734	1.5630	-3.8254	-1.7681	1.5429	-3.8319	-1.7645
C	-4.0086	-1.8559	0.3076	3.9704	-1.8631	-0.3344	3.9575	-1.8722	-0.3412
H	-5.0750	-1.8258	0.1059	5.0339	-1.8644	-0.1182	5.0219	-1.8760	-0.1296
C	-1.2394	-1.9084	0.9215	1.1960	-1.8562	-0.9966	1.1904	-1.8603	-0.9843
H	-0.1945	-1.9310	1.2044	0.1552	-1.8481	-1.2933	0.1459	-1.8523	-1.2691
N	3.5465	0.5043	0.4077	-3.5354	0.4884	-0.3851	-3.5481	0.4709	-0.4506
N	-3.5465	0.5043	-0.4077	3.5457	0.4792	0.4465	3.5481	0.4709	0.4506
C	-1.1937	3.3484	-0.2942	1.1703	3.3526	0.2961	1.1943	3.3554	0.3026
H	-1.1412	4.4339	-0.3277	1.1290	4.4362	0.3659	1.1504	4.4388	0.3681
C	4.9048	0.9508	0.6450	-4.9004	0.8715	-0.6826	-4.9144	0.8886	-0.6806
H	5.4885	0.1303	1.0688	-5.4182	0.0352	-1.1589	-5.4957	0.0456	-1.0616
H	5.3929	1.2936	-0.2768	-5.4460	1.1561	0.2265	-5.3927	1.2660	0.2340
H	4.9018	1.7680	1.3698	-4.8981	1.7145	-1.3762	-4.9297	1.6765	-1.4380
C	-4.9048	0.9508	-0.6450	4.9056	0.9027	0.7055	4.9144	0.8886	0.6806
H	-5.3929	1.2936	0.2768	5.3963	1.2907	-0.1974	5.3927	1.2660	-0.2341
H	-4.9018	1.7680	-1.3698	4.9028	1.6835	1.4703	4.9298	1.6765	1.4380
H	-5.4885	0.1303	-1.0688	5.4848	0.0590	1.0880	5.4957	0.0456	1.0615

All calculations are at B3LYP/6-31G(d) in toluene.

Table S11. Cartesian coordinates of optimized S_0, S_1, and T_1 states of MeCCz-d6.

Elements	X	Y	Z	X	Y	Z	X	Y	Z
		S_0			S_1			T_1	
C	2.0551	-3.0032	-1.1947	-1.9860	-3.0026	1.2336	-1.9853	-2.9711	1.2716
C	3.4229	-2.9853	-0.8734	-3.3427	-3.0086	0.9201	-3.3456	-2.9839	0.9400
C	4.0086	-1.8559	-0.3076	-3.9559	-1.8751	0.3480	-3.9575	-1.8722	0.3412
C	3.1872	-0.7548	-0.0475	-3.1523	-0.7720	0.0896	-3.1645	-0.7631	0.0652
C	1.7852	-0.7707	-0.3022	-1.7523	-0.7564	0.3485	-1.7617	-0.7428	0.3455
C	1.2394	-1.9084	-0.9215	-1.1797	-1.8832	0.9571	-1.1904	-1.8603	0.9844
H	1.6279	-3.8800	-1.6734	-1.5373	-3.8736	1.7031	-1.5429	-3.8319	1.7646
H	4.0398	-3.8525	-1.0933	-3.9470	-3.8851	1.1365	-3.9478	-3.8591	1.1687
H	5.0750	-1.8258	-0.1059	-5.0224	-1.8641	0.1475	-5.0219	-1.8760	0.1295
H	0.1945	-1.9310	-1.2044	-0.1333	-1.8896	1.2332	-0.1459	-1.8523	1.2691

C	2.4240	1.3149	0.3837	-2.4573	1.3387	-0.3642	-2.4605	1.3347	-0.3960
C	2.3874	2.7158	0.5453	-2.4326	2.7180	-0.5529	-2.4291	2.6976	-0.5503
C	1.1937	3.3484	0.2942	-1.2164	3.3666	-0.3327	-1.1943	3.3555	-0.3026
C	0.0000	2.6289	0.0000	-0.0131	2.6580	-0.0273	0.0000	2.6520	0.0000
C	0.0000	1.1870	0.0000	-0.0082	1.2005	-0.0178	0.0000	1.2016	0.0000
C	1.2826	0.5488	0.0446	-1.2720	0.5653	-0.0146	-1.2752	0.5611	-0.0246
H	3.2818	3.2822	0.7833	-3.3277	3.2781	-0.8013	-3.3164	3.2710	-0.7974
H	1.1412	4.4339	0.3277	-1.1630	4.4491	-0.4097	-1.1504	4.4388	-0.3681
C	-1.2826	0.5488	-0.0446	1.2762	0.5577	0.0256	1.2752	0.5611	0.0247
C	-2.3874	2.7158	-0.5453	2.3995	2.6988	0.5575	2.4291	2.6976	0.5503
H	-3.2818	3.2822	-0.7833	3.2828	3.2779	0.8068	3.3165	3.2710	0.7974
C	-2.4240	1.3149	-0.3837	2.4424	1.3293	0.4075	2.4605	1.3347	0.3960
C	-1.7852	-0.7707	0.3022	1.7630	-0.7323	-0.3512	1.7617	-0.7428	-0.3455
C	-3.1872	-0.7548	0.0475	3.1723	-0.7511	-0.0645	3.1645	-0.7631	-0.0653
C	-3.4229	-2.9853	0.8734	3.3611	-2.9732	-0.9319	3.3455	-2.9839	-0.9400
H	-4.0398	-3.8525	1.0933	3.9626	-3.8496	-1.1575	3.9477	-3.8591	-1.1686
C	-2.0551	-3.0032	1.1947	1.9970	-2.9612	-1.2728	1.9853	-2.9711	-1.2716
H	-1.6279	-3.8800	1.6734	1.5630	-3.8254	-1.7681	1.5429	-3.8319	-1.7645
C	-4.0086	-1.8559	0.3076	3.9704	-1.8631	-0.3344	3.9575	-1.8722	-0.3412
H	-5.0750	-1.8258	0.1059	5.0339	-1.8644	-0.1182	5.0219	-1.8760	-0.1296
C	-1.2394	-1.9084	0.9215	1.1960	-1.8562	-0.9966	1.1904	-1.8603	-0.9843
H	-0.1945	-1.9310	1.2044	0.1552	-1.8481	-1.2933	0.1459	-1.8523	-1.2691
N	3.5465	0.5043	0.4077	-3.5354	0.4884	-0.3851	-3.5481	0.4709	-0.4506
N	-3.5465	0.5043	-0.4077	3.5457	0.4792	0.4465	3.5481	0.4709	0.4506
C	-1.1937	3.3484	-0.2942	1.1703	3.3526	0.2961	1.1943	3.3554	0.3026
H	-1.1412	4.4339	-0.3277	1.1290	4.4362	0.3659	1.1504	4.4388	0.3681
C	4.9048	0.9508	0.6450	-4.9004	0.8715	-0.6826	-4.9144	0.8886	-0.6806
D	5.4885	0.1303	1.0688	-5.4182	0.0352	-1.1589	-5.4957	0.0456	-1.0616
D	5.3929	1.2936	-0.2768	-5.4460	1.1561	0.2265	-5.3927	1.2660	0.2340
D	4.9018	1.7680	1.3698	-4.8981	1.7145	-1.3762	-4.9297	1.6765	-1.4380
C	-4.9048	0.9508	-0.6450	4.9056	0.9027	0.7055	4.9144	0.8886	0.6806
D	-5.3929	1.2936	0.2768	5.3963	1.2907	-0.1974	5.3927	1.2660	-0.2341
D	-4.9018	1.7680	-1.3698	4.9028	1.6835	1.4703	4.9298	1.6765	1.4380
D	-5.4885	0.1303	-1.0688	5.4848	0.0590	1.0880	5.4957	0.0456	1.0615

All calculations are at B3LYP/6-31G(d) in toluene.

Table S12. Cartesian coordinates of optimized S_0, S_1, and T_1 states of PhCCz.

Elements	X	Y	Z	X	Y	Z	X	Y	Z
		S_0			S_1			T_1	
C	1.8898	-3.4018	-1.4034	1.7803	-3.3551	-1.4608	1.7885	-3.3398	-1.4764
C	3.2824	-3.4006	-1.2145	3.1642	-3.3840	-1.3041	3.1750	-3.3768	-1.2796
C	3.9355	-2.2737	-0.7233	3.8570	-2.2562	-0.8254	3.8657	-2.2678	-0.7702
C	3.1585	-1.1548	-0.4105	3.1105	-1.1276	-0.5077	3.1286	-1.1318	-0.4526
C	1.7388	-1.1606	-0.5059	1.6901	-1.1002	-0.5757	1.7045	-1.0963	-0.5649
C	1.1190	-2.2958	-1.0575	1.0315	-2.2218	-1.0980	1.0489	-2.2115	-1.1239
H	1.4064	-4.2769	-1.8287	1.2644	-4.2221	-1.8639	1.2819	-4.1987	-1.9068
H	3.8630	-4.2814	-1.4749	3.7242	-4.2764	-1.5687	3.7333	-4.2715	-1.5412
H	5.0142	-2.2558	-0.6081	4.9362	-2.2686	-0.7235	4.9427	-2.2918	-0.6465
H	0.0495	-2.3096	-1.2257	-0.0419	-2.2166	-1.2343	-0.0208	-2.1893	-1.2890
C	2.4487	0.9215	0.1227	2.4752	0.9856	0.0662	2.4887	0.9761	0.1042
C	2.4326	2.3227	0.2816	2.4766	2.3696	0.2542	2.4757	2.3384	0.2624
C	1.2198	2.9556	0.1592	1.2486	3.0197	0.1986	1.2220	2.9965	0.1587
C	0.0000	2.2364	0.0000	0.0142	2.3097	0.0553	0.0000	2.2933	0.0000
C	0.0000	0.7960	0.0000	0.0109	0.8482	0.0364	0.0000	0.8446	0.0000
C	1.2804	0.1565	-0.0935	1.2641	0.2185	-0.1349	1.2702	0.2045	-0.1292
H	3.3521	2.8804	0.4210	3.4012	2.9213	0.3796	3.3886	2.9055	0.4070
H	1.1723	4.0412	0.1946	1.2074	4.1023	0.2770	1.1871	4.0798	0.2298
C	-1.2804	0.1565	0.0935	-1.2690	0.2010	0.1498	-1.2702	0.2045	0.1292
C	-2.4326	2.3227	-0.2816	-2.4480	2.3387	-0.2123	-2.4757	2.3384	-0.2624
H	-3.3521	2.8804	-0.4210	-3.3630	2.9090	-0.3317	-3.3886	2.9056	-0.4069
C	-2.4487	0.9215	-0.1227	-2.4650	0.9650	-0.0819	-2.4887	0.9761	-0.1042
C	-1.7388	-1.1606	0.5059	-1.6957	-1.0871	0.5976	-1.7045	-1.0963	0.5649
C	-3.1585	-1.1548	0.4105	-3.1305	-1.1245	0.4943	-3.1286	-1.1318	0.4526
C	-3.2824	-3.4006	1.2145	-3.1719	-3.3762	1.3029	-3.1750	-3.3768	1.2796
H	-3.8630	-4.2814	1.4749	-3.7221	-4.2774	1.5589	-3.7333	-4.2715	1.5412
C	-1.8898	-3.4018	1.4034	-1.7778	-3.3318	1.5020	-1.7885	-3.3398	1.4764
H	-1.4064	-4.2769	1.8287	-1.2728	-4.1933	1.9301	-1.2819	-4.1987	1.9068
C	-3.9355	-2.2737	0.7232	-3.8661	-2.2717	0.8054	-3.8657	-2.2678	0.7702
H	-5.0142	-2.2558	0.6081	-4.9427	-2.2992	0.6798	-4.9427	-2.2918	0.6465
C	-1.1190	-2.2958	1.0575	-1.0370	-2.2059	1.1593	-1.0489	-2.2115	1.1239
H	-0.0495	-2.3096	1.2257	0.0311	-2.1800	1.3322	0.0208	-2.1893	1.2890
N	3.5751	0.1119	-0.0044	3.5619	0.1407	-0.0883	3.5851	0.1128	-0.0045

N	-3.5751	0.1119	0.0044	-3.5806	0.1128	0.0557	-3.5851	0.1128	0.0045
C	-1.2198	2.9556	-0.1592	-1.2016	3.0011	-0.1053	-1.2220	2.9965	-0.1587
H	-1.1723	4.0412	-0.1946	-1.1776	4.0856	-0.1649	-1.1871	4.0798	-0.2297
C	-4.9236	0.5397	-0.1588	-4.9291	0.4967	-0.1592	-4.9428	0.4990	-0.1612
C	-5.8315	0.4331	0.9021	-5.9027	0.2664	0.8242	-5.8670	0.3224	0.8773
C	-5.3403	1.0732	-1.3845	-5.2898	1.1255	-1.3602	-5.3565	1.0723	-1.3709
C	-7.1518	0.8493	0.7297	-7.2244	0.6475	0.5959	-7.1967	0.7036	0.6964
H	-5.4968	0.0349	1.8548	-5.6168	-0.1928	1.7648	-5.5387	-0.1015	1.8209
C	-6.6576	1.5045	-1.5428	-6.6109	1.5157	-1.5723	-6.6837	1.4672	-1.5365
H	-4.6329	1.1389	-2.2055	-4.5347	1.2920	-2.1220	-4.6371	1.1975	-2.1742
C	-7.5675	1.3891	-0.4896	-7.5847	1.2746	-0.5992	-7.6092	1.2795	-0.5071
H	-7.8526	0.7626	1.5554	-7.9719	0.4656	1.3632	-7.9081	0.5616	1.5054
H	-6.9747	1.9198	-2.4953	-6.8815	1.9990	-2.5071	-6.9965	1.9114	-2.4775
H	-8.5947	1.7183	-0.6180	-8.6144	1.5750	-0.7701	-8.6441	1.5810	-0.6416
C	4.9236	0.5397	0.1588	4.9175	0.5067	0.1203	4.9428	0.4990	0.1612
C	5.8315	0.4331	-0.9021	5.8761	0.2740	-0.8771	5.8670	0.3224	-0.8773
C	5.3403	1.0732	1.3845	5.2953	1.1128	1.3275	5.3565	1.0723	1.3709
C	7.1518	0.8493	-0.7297	7.2036	0.6389	-0.6580	7.1967	0.7036	-0.6965
H	5.4968	0.0349	-1.8548	5.5739	-0.1686	-1.8202	5.5387	-0.1015	-1.8209
C	6.6576	1.5045	1.5428	6.6239	1.4789	1.5326	6.6837	1.4672	1.5365
H	4.6329	1.1389	2.2055	4.5493	1.2769	2.0983	4.6371	1.1975	2.1742
C	7.5675	1.3891	0.4896	7.5830	1.2417	0.5438	7.6092	1.2795	0.5071
H	7.8526	0.7626	-1.5554	7.9409	0.4611	-1.4358	7.9081	0.5616	-1.5054
H	6.9747	1.9198	2.4953	6.9115	1.9418	2.4723	6.9965	1.9114	2.4774
H	8.5947	1.7183	0.6180	8.6182	1.5260	0.7084	8.6441	1.5810	0.6416

All calculations are at B3LYP/6-31G(d) in toluene.

Table S13. Cartesian coordinates of optimized S_0, S_1, and T_1 states of *p*CNPhCCz.

**Elements**	**X**	**Y**	**Z**	**X**	**Y**	**Z**	**X**	**Y**	**Z**
		S_0			S_1			T_1	
C	-1.8188	-3.5698	1.4785	1.8662	3.5420	1.5739	-1.7033	-3.4899	1.5562
C	-3.2155	-3.5811	1.3304	3.2591	3.5115	1.4380	-3.0959	-3.5399	1.4088
C	-3.8933	-2.4603	0.8582	3.9122	2.3820	0.9240	-3.8148	-2.4353	0.9314
C	-3.1366	-1.3337	0.5250	3.1222	1.3128	0.5343	-3.1022	-1.2895	0.5960
C	-1.7161	-1.3307	0.5720	1.7045	1.3395	0.5925	-1.6762	-1.2456	0.6476

C	-1.0688	-2.4586	1.1053	1.0816	2.4629	1.1601	-0.9888	-2.3559	1.1779
H	-1.3152	-4.4398	1.8902	1.3867	4.4118	2.0119	-1.1736	-4.3451	1.9654
H	-3.7807	-4.4668	1.6065	3.8492	4.3653	1.7569	-3.6359	-4.4414	1.6834
H	-4.9743	-2.4604	0.7696	4.9923	2.3255	0.8547	-4.8939	-2.4789	0.8401
H	0.0057	-2.4636	1.2365	0.0077	2.4954	1.2948	0.0865	-2.3247	1.2989
C	-2.4498	0.7470	-0.0493	2.4652	-0.7366	-0.0626	-2.4898	0.8212	0.0006
C	-2.4395	2.1489	-0.2029	2.4569	-2.1288	-0.3099	-2.4815	2.1861	-0.1475
C	-1.2242	2.7833	-0.1181	1.2448	-2.7537	-0.2689	-1.2271	2.8422	-0.0977
C	0.0000	2.0648	0.0000	0.0072	-2.0317	-0.1177	0.0000	2.1381	0.0000
C	0.0000	0.6252	0.0000	-0.0043	-0.5869	-0.0600	0.0000	0.6907	0.0000
C	-1.2762	-0.0148	0.1337	1.2516	0.0403	0.1346	-1.2633	0.0516	0.1851
H	-3.3609	2.7108	-0.3076	3.3895	-2.6606	-0.4497	-3.3967	2.7590	-0.2444
H	-1.1802	3.8688	-0.1514	1.1864	-3.8337	-0.3682	-1.1961	3.9257	-0.1636
C	1.2762	-0.0148	-0.1337	-1.2892	0.0521	-0.1624	1.2633	0.0516	-0.1850
C	2.4395	2.1489	0.2029	-2.4311	-2.1243	0.1104	2.4815	2.1861	0.1475
H	3.3609	2.7108	0.3076	-3.3410	-2.7058	0.2053	3.3967	2.7590	0.2444
C	2.4498	0.7470	0.0493	-2.4640	-0.7348	0.0123	2.4898	0.8212	-0.0006
C	1.7161	-1.3307	-0.5720	-1.7435	1.3657	-0.5366	1.6762	-1.2456	-0.6475
C	3.1366	-1.3337	-0.5250	-3.1691	1.3526	-0.4594	3.1022	-1.2895	-0.5960
C	3.2155	-3.5811	-1.3304	-3.2773	3.6222	-1.1787	3.0958	-3.5398	-1.4088
H	3.7807	-4.4668	-1.6065	-3.8507	4.5141	-1.4125	3.6359	-4.4414	-1.6834
C	1.8188	-3.5698	-1.4785	-1.8754	3.6339	-1.3502	1.7033	-3.4899	-1.5562
H	1.3152	-4.4398	-1.8902	-1.3929	4.5261	-1.7364	1.1736	-4.3451	-1.9654
C	3.8933	-2.4603	-0.8582	-3.9418	2.4846	-0.7406	3.8148	-2.4353	-0.9315
H	4.9743	-2.4604	-0.7696	-5.0211	2.4698	-0.6388	4.8939	-2.4789	-0.8401
C	1.0688	-2.4586	-1.1053	-1.1103	2.5234	-1.0362	0.9888	-2.3559	-1.1779
H	-0.0057	-2.4636	-1.2365	-0.0382	2.5425	-1.1852	-0.0865	-2.3247	-1.2989
N	-3.5729	-0.0672	0.1226	3.5529	0.0422	0.1116	-3.5842	-0.0420	0.1622
N	3.5729	-0.0672	-0.1226	-3.5910	0.0779	-0.1011	3.5842	-0.0420	-0.1622
C	1.2242	2.7833	0.1181	-1.1922	-2.7529	-0.0097	1.2271	2.8422	0.0978
H	1.1802	3.8688	0.1514	-1.1487	-3.8378	-0.0102	1.1961	3.9257	0.1637
C	4.9182	0.3502	0.0138	-4.9397	-0.3614	0.0174	4.9368	0.3267	-0.0089
C	5.8383	0.1331	-1.0229	-5.8262	-0.2072	-1.0559	5.8837	0.0217	-1.0019
C	5.3346	0.9894	1.1913	-5.3715	-0.9524	1.2113	5.3443	1.0198	1.1442
C	7.1597	0.5394	-0.8809	-7.1445	-0.6322	-0.9326	7.2130	0.3886	-0.8386
H	5.5104	-0.3379	-1.9429	-5.4793	0.2324	-1.9850	5.5702	-0.4812	-1.9097
C	6.6512	1.4116	1.3307	-6.6849	-1.3933	1.3326	6.6702	1.3968	1.3052

H	4.6259	1.1390	1.9989	-4.6825	-1.0517	2.0436	4.6179	1.2437	1.9180
C	7.5753	1.1844	0.2966	-7.5797	-1.2301	0.2620	7.6200	1.0808	0.3159
H	7.8696	0.3741	-1.6844	-7.8347	-0.5162	-1.7612	7.9388	0.1569	-1.6112
H	6.9724	1.9029	2.2431	-7.0243	-1.8500	2.2560	6.9810	1.9231	2.2017
C	-4.9182	0.3502	-0.0138	4.9255	-0.3759	-0.0261	-4.9368	0.3267	0.0089
C	-5.8383	0.1331	1.0229	5.6429	-0.8680	1.1010	-5.8837	0.0216	1.0019
C	-5.3346	0.9894	-1.1913	5.5781	-0.2790	-1.2869	-5.3443	1.0198	-1.1442
C	-7.1597	0.5394	0.8809	6.9516	-1.2616	0.9733	-7.2130	0.3886	0.8386
H	-5.5104	-0.3379	1.9429	5.1459	-0.9339	2.0656	-5.5702	-0.4812	1.9096
C	-6.6512	1.4116	-1.3307	6.8869	-0.6692	-1.4245	-6.6702	1.3968	-1.3052
H	-4.6259	1.1390	-1.9989	5.0315	0.1064	-2.1440	-4.6179	1.2437	-1.9180
C	-7.5753	1.1844	-0.2966	7.6364	-1.1843	-0.2988	-7.6200	1.0808	-0.3160
H	-7.8697	0.3741	1.6844	7.4898	-1.6399	1.8371	-7.9388	0.1568	1.6111
H	-6.9724	1.9029	-2.2431	7.3755	-0.5939	-2.3909	-6.9810	1.9231	-2.2017
C	8.9358	1.6096	0.4418	8.9667	-1.5910	-0.4345	8.9889	1.4630	0.4822
N	10.0407	1.9552	0.5597	10.0903	-1.9355	-0.5488	10.1028	1.7736	0.6179
C	-8.9358	1.6096	-0.4418	-8.9374	-1.6738	0.3885	-8.9889	1.4630	-0.4823
N	-10.0407	1.9552	-0.5597	-10.0387	-2.0336	0.4913	-10.1028	1.7736	-0.6179

All calculations are at B3LYP/6-31G(d) in toluene.

Table S14. Cartesian coordinates of optimized S_0, S_1, and T_1 states of *p*CzPhCCz.

**Elements**	**X**	**Y**	**Z**	**X**	**Y**	**Z**	**X**	**Y**	**Z**
		S_0			S_1			T_1	
C	-1.8049	4.6023	-0.5523	1.6620	4.4646	0.6901	-1.6909	4.47916	-0.68075
C	-3.2069	4.5769	-0.4613	3.0552	4.4842	0.6490	-3.0879	4.49107	-0.57731
C	-3.8885	3.3782	-0.2705	3.7829	3.2922	0.4890	-3.8106	3.30672	-0.37342
C	-3.1312	2.2092	-0.1568	3.0663	2.1064	0.3652	-3.0960	2.11888	-0.2632
C	-1.7088	2.2186	-0.1533	1.6449	2.0720	0.3068	-1.6677	2.09002	-0.27886
C	-1.0562	3.4387	-0.4031	0.9464	3.2684	0.5162	-0.9761	3.29147	-0.53277
H	-1.2963	5.5428	-0.7447	1.1141	5.3890	0.8504	-1.1569	5.40443	-0.87634
H	-3.7710	5.5000	-0.5626	3.5915	5.4222	0.7585	-3.6281	5.42873	-0.67376
H	-4.9725	3.3500	-0.2327	4.8664	3.3060	0.4718	-4.8935	3.32007	-0.32051
H	0.0222	3.4759	-0.4920	-0.1349	3.2748	0.5541	0.1027	3.29137	-0.62264
C	-2.4514	0.0572	-0.0386	2.4630	-0.0895	0.1961	-2.4891	-0.06891	-0.13473
C	-2.4405	-1.3448	-0.1885	2.4671	-1.4810	0.3314	-2.4807	-1.43248	-0.28158

C	-1.2198	-1.9493	-0.3647	1.2362	-2.1191	0.4169	-1.2195	-2.06598	-0.43509
C	0.0048	-1.2258	-0.2815	0.0009	-1.4116	0.2816	0.0070	-1.35929	-0.34316
C	-0.0011	0.1800	0.0321	0.0108	0.0205	-0.0205	-0.0004	0.05408	-0.02566
C	-1.2748	0.8383	-0.0052	1.2522	0.6849	0.1173	-1.2609	0.72117	-0.10524
H	-3.3645	-1.9104	-0.2370	3.3924	-2.0374	0.4289	-3.3978	-2.00836	-0.33463
H	-1.1708	-3.0169	-0.5636	1.1951	-3.1913	0.5853	-1.1848	-3.13852	-0.60243
C	1.2672	0.7701	0.3480	-1.2448	0.6128	-0.3901	1.2527	0.63544	0.33551
C	2.4508	-1.2747	-0.4117	-2.4696	-1.4140	0.3052	2.4949	-1.376	-0.41983
H	3.3793	-1.7998	-0.6070	-3.3987	-1.9548	0.4510	3.4180	-1.91055	-0.61412
C	2.4501	0.0581	0.0491	-2.4580	-0.0960	-0.1170	2.4889	-0.08096	0.03216
C	1.6904	1.9605	1.0689	-1.6162	1.7739	-1.1442	1.6455	1.80446	1.07731
C	3.1128	1.9616	1.0711	-3.0518	1.8147	-1.1917	3.0732	1.84824	1.08452
C	3.1702	3.9768	2.3518	-2.9996	3.8452	-2.4581	3.0425	3.86022	2.37904
H	3.7271	4.7739	2.8364	-3.5151	4.6657	-2.9489	3.5737	4.67137	2.86924
C	1.7681	3.9504	2.4417	-1.5945	3.7729	-2.5099	1.6455	3.79528	2.45812
H	1.2523	4.7164	3.0139	-1.0418	4.5285	-3.0616	1.1021	4.54533	3.02537
C	3.8611	2.9776	1.6718	-3.7452	2.8625	-1.8057	3.7768	2.88085	1.69499
H	4.9452	2.9767	1.6280	-4.8283	2.9067	-1.7880	4.8597	2.92276	1.6599
C	1.0284	2.9542	1.8114	-0.8994	2.7536	-1.8654	0.9422	2.77832	1.81399
H	-0.0501	2.9416	1.9055	0.1800	2.7055	-1.9278	-0.1368	2.73209	1.88882
N	-3.5703	0.8884	-0.0627	3.5477	0.7833	0.2674	-3.5797	0.81061	-0.13132
N	3.5623	0.8092	0.4261	-3.5518	0.6913	-0.5438	3.5702	0.72496	0.41093
C	1.2352	-1.9063	-0.5116	-1.2317	-2.0700	0.4658	1.2407	-2.02546	-0.55873
H	1.1951	-2.9574	-0.7861	-1.2228	-3.1176	0.7531	1.2169	-3.06648	-0.86738
C	4.9180	0.4127	0.2677	-4.9123	0.3536	-0.3666	4.9356	0.36956	0.26918
C	5.7577	0.2908	1.3821	-5.8140	0.4036	-1.4427	5.7878	0.33268	1.38154
C	5.4217	0.1357	-1.0093	-5.3754	-0.0544	0.8953	5.4421	0.03796	-0.99509
C	7.0892	-0.0828	1.2186	-7.1529	0.0774	-1.2527	7.1299	-0.00478	1.22673
H	5.3668	0.4942	2.3736	-5.4615	0.6907	-2.4273	5.3988	0.57154	2.3657
C	6.7427	-0.2753	-1.1688	-6.7079	-0.4076	1.0758	6.7747	-0.33579	-1.14315
H	4.7733	0.2371	-1.8738	-4.6848	-0.0908	1.7314	4.7852	0.06841	-1.8585
C	7.5889	-0.3774	-0.0569	-7.6121	-0.3371	0.0054	7.6320	-0.34967	-0.03471
H	7.7452	-0.1508	2.0804	-7.8483	0.1382	-2.0837	7.7925	-0.0069	2.08627
H	7.1230	-0.5157	-2.1563	-7.0544	-0.7387	2.0495	7.1566	-0.61471	-2.11992
C	-4.9225	0.4512	-0.0850	4.9027	0.3988	0.2370	-4.9409	0.41461	-0.13418
C	-5.7678	0.8081	-1.1431	5.8245	0.9267	1.1600	-5.8163	0.8538	-1.13706
C	-5.4169	-0.3471	0.9539	5.3475	-0.5361	-0.7156	-5.4203	-0.43709	0.87077

C	-7.0904	0.3725	-1.1611	7.1548	0.5295	1.1270	-7.1498	0.45215	-1.13036
H	-5.3802	1.4052	-1.9621	5.4840	1.6069	1.9324	-5.4456	1.48753	-1.93574
C	-6.7399	-0.7818	0.9368	6.6803	-0.9206	-0.7546	-6.7534	-0.83704	0.87755
H	-4.7685	-0.6058	1.7850	4.6483	-0.9240	-1.4486	-4.7492	-0.76506	1.65808
C	-7.5862	-0.4247	-0.1211	7.6009	-0.3945	0.1683	-7.6291	-0.39475	-0.12304
H	-7.7359	0.6284	-1.9950	7.8492	0.9085	1.8695	-7.8181	0.77428	-1.92257
H	-7.1272	-1.3789	1.7559	7.0224	-1.6068	-1.5220	-7.1261	-1.4747	1.67276
N	8.9412	-0.7761	-0.2209	-8.9750	-0.6850	0.1935	8.9966	-0.71093	-0.18855
C	9.8801	-0.1642	-1.0612	-9.8372	-0.1336	1.1494	9.9116	-0.10469	-1.05805
C	9.5651	-1.8462	0.4331	-9.6866	-1.6406	-0.5425	9.6579	-1.73021	0.5075
C	11.1173	-0.8480	-0.9440	-11.1134	-0.7403	1.0244	11.1717	-0.74096	-0.91775
C	10.9159	-1.9217	0.0070	-11.0171	-1.7024	-0.0540	11.0094	-1.77893	0.07929
N	-8.9345	-0.8677	-0.1395	8.9565	-0.7926	0.1331	-8.9892	-0.80294	-0.11632
C	-10.0653	-0.0483	-0.2510	10.0657	0.0667	0.1173	-10.1006	0.04869	-0.11096
C	-9.3611	-2.1988	-0.0480	9.4241	-2.1154	0.1016	-9.4471	-2.12616	-0.1098
C	-11.2270	-0.8618	-0.2300	11.2502	-0.7116	0.0748	-11.2824	-0.73598	-0.09995
C	-10.7781	-2.2329	-0.1029	10.8414	-2.1017	0.0667	-10.8656	-2.12297	-0.10118
C	9.8767	-3.7602	1.8291	-10.1676	-3.3615	-2.1290	10.0375	-3.56993	1.98415
H	9.4842	-4.4869	2.5351	-9.8493	-4.0185	-2.9339	9.6717	-4.27787	2.72288
C	12.0835	0.6998	-2.5152	-11.9150	0.6543	2.8161	12.0792	0.76925	-2.55905
H	12.9329	1.0502	-3.0946	-12.7142	0.9728	3.4793	12.9142	1.12264	-3.15716
C	10.8534	1.3741	-2.6090	-10.6478	1.2550	2.9171	10.8265	1.39686	-2.67596
H	10.7669	2.2419	-3.2572	-10.4829	2.0353	3.6551	10.7084	2.23215	-3.36088
C	-11.4181	1.9145	-0.4155	11.3606	2.0744	0.0507	-11.4046	2.05034	-0.06107
H	-11.5082	2.9953	-0.4821	11.4183	3.1591	0.0326	-11.4679	3.13492	-0.03994
C	-12.5795	1.1221	-0.4101	12.5443	1.3174	0.0270	-12.5861	1.2881	-0.06311
H	-13.5538	1.5977	-0.4771	13.5047	1.8238	-0.0034	-13.5489	1.79082	-0.04807
C	11.2218	-3.8414	1.4280	-11.4938	-3.4259	-1.6665	11.3834	-3.62377	1.58092
H	11.8554	-4.6262	1.8311	-12.1871	-4.1276	-2.1212	12.0441	-4.36884	2.01487
C	11.7450	-2.9287	0.5161	-11.9223	-2.6028	-0.6284	11.8732	-2.73471	0.62804
H	12.7823	-2.9986	0.1992	-12.9449	-2.6611	-0.2644	12.9114	-2.78372	0.31018
C	9.0304	-2.7677	1.3381	-9.2467	-2.4741	-1.5751	9.1570	-2.62849	1.45435
H	7.9914	-2.7187	1.6467	-8.2228	-2.4383	-1.9320	8.1178	-2.60007	1.76503
C	12.2221	-0.4076	-1.6830	-12.1543	-0.3395	1.8707	12.2582	-0.29592	-1.68065
H	13.1763	-0.9219	-1.6037	-13.1372	-0.7957	1.7865	13.2295	-0.77394	-1.58416
C	9.7382	0.9564	-1.8849	-9.5948	0.8745	2.0872	9.7287	0.97338	-1.92902
H	8.7958	1.4891	-1.9568	-8.6229	1.3502	2.1659	8.7680	1.46941	-2.01944

C	-8.6041	-3.3704	0.0442	8.7057	-3.3139	0.1477	-8.7180	-3.31844	-0.14394
H	-7.5198	-3.3420	0.0663	7.6227	-3.3234	0.2035	-7.6333	-3.3161	-0.16939
C	-9.2894	-4.5826	0.1011	9.4295	-4.5051	0.1301	-9.4321	-4.51515	-0.15041
H	-8.7224	-5.5066	0.1748	8.8926	-5.4490	0.1626	-8.8873	-5.455	-0.1754
C	-10.6939	-4.6336	0.0617	10.8337	-4.5086	0.0765	-10.8379	-4.5305	-0.12857
H	-11.1975	-5.5949	0.1098	11.3678	-5.4543	0.0623	-11.3642	-5.48075	-0.13237
C	-11.4420	-3.4643	-0.0437	11.5446	-3.3111	0.0501	-11.5587	-3.33965	-0.10736
H	-12.5273	-3.5056	-0.0842	12.6310	-3.3148	0.0234	-12.6455	-3.35259	-0.10022
C	-10.1489	1.3447	-0.3337	10.1081	1.4638	0.0919	-10.1492	1.4455	-0.08239
H	-9.2590	1.9653	-0.3294	9.2008	2.0582	0.0952	-9.2426	2.04149	-0.07216
C	-12.4903	-0.2639	-0.3141	12.4952	-0.0746	0.0334	-12.5312	-0.10291	-0.0791
H	-13.3892	-0.8748	-0.2997	13.4108	-0.6592	0.0004	-13.4451	-0.69123	-0.07106

All calculations are at B3LYP/6-31G(d) in toluene.

Table S15. Cartesian coordinates of optimized S_0, S_1, and T_1 states of SpiroCCz.

**Elements**	**X**	**Y**	**Z**	**X**	**Y**	**Z**	**X**	**Y**	**Z**
		S_0			S_1			T_1	
C	3.7352	-4.5919	-2.4849	3.8672	-4.2716	-2.8969	3.8436	-4.1521	-3.0155
C	3.5385	-3.3960	-3.1856	3.6850	-3.0004	-3.4545	3.7110	-2.8543	-3.5238
H	3.3771	-3.4205	-4.2598	3.5638	-2.8951	-4.5291	3.6151	-2.7022	-4.5954
C	3.5463	-2.1687	-2.5134	3.6547	-1.8640	-2.6388	3.6993	-1.7515	-2.6627
H	3.3912	-1.2415	-3.0586	3.5104	-0.8777	-3.0720	3.5955	-0.7442	-3.0577
C	3.7549	-2.1565	-1.1390	3.8107	-2.0188	-1.2664	3.8242	-1.9660	-1.2951
C	3.9508	-3.3572	-0.4315	3.9912	-3.2949	-0.7019	3.9554	-3.2696	-0.7806
C	4.1383	-3.0401	0.9914	4.1214	-3.1523	0.7551	4.0619	-3.1888	0.6833
C	4.0509	-1.6452	1.1583	4.0162	-1.7889	1.0872	3.9910	-1.8358	1.0662
C	4.1946	-1.0711	2.4157	4.1034	-1.3704	2.4091	4.0643	-1.4728	2.4057
H	4.1287	0.0056	2.5484	4.0173	-0.3180	2.6668	4.0073	-0.4287	2.7024
C	4.4259	-1.9042	3.5165	4.3013	-2.3295	3.4091	4.2139	-2.4755	3.3705
C	4.5121	-3.2916	3.3542	4.4070	-3.6866	3.0832	4.2858	-3.8216	2.9929
H	4.6920	-3.9249	4.2188	4.5591	-4.4192	3.8712	4.4011	-4.5885	3.7541
H	4.5391	-1.4688	4.5055	4.3720	-2.0170	4.4473	4.2740	-2.2065	4.4215
C	4.3691	-3.8701	2.0907	4.3169	-4.1085	1.7544	4.2098	-4.1889	1.6473
H	4.4371	-4.9483	1.9697	4.3961	-5.1641	1.5071	4.2643	-5.2359	1.3602
C	3.7913	-0.9487	-0.1939	3.8050	-0.9281	-0.1800	3.8249	-0.9231	-0.1649

C	4.9422	0.0228	-0.5270	4.9672	0.0645	-0.3634	5.0004	0.0689	-0.2966
C	6.1762	-0.4892	-0.9438	6.2379	-0.4289	-0.6792	6.2763	-0.4372	-0.5735
C	7.2560	0.3371	-1.2460	7.3339	0.4088	-0.8674	7.3867	0.3882	-0.7254
H	8.2006	-0.0961	-1.5611	8.3041	-0.0134	-1.1113	8.3614	-0.0424	-0.9345
C	7.1002	1.7202	-1.1606	7.1655	1.7935	-0.7683	7.2231	1.7707	-0.6303
H	7.9162	2.3859	-1.4271	7.9977	2.4655	-0.9565	8.0661	2.4387	-0.7838
C	5.8862	2.2608	-0.7481	5.9226	2.3193	-0.4466	5.9706	2.3073	-0.3534
H	5.7567	3.3344	-0.7403	5.7845	3.3914	-0.4320	5.8448	3.3812	-0.3406
H	6.2863	-1.5651	-1.0373	6.3636	-1.5016	-0.7888	6.3936	-1.5119	-0.6779
C	4.8160	1.4246	-0.3933	4.8256	1.4694	-0.2018	4.8601	1.4705	-0.1465
N	3.5783	1.9555	0.0283	3.5684	1.9788	0.1352	3.5891	1.9951	0.1413
C	3.1957	3.2419	0.4306	3.1537	3.2475	0.5714	3.1558	3.2515	0.5938
C	3.9759	4.3225	0.8558	3.9033	4.3250	1.0491	3.8853	4.3362	1.0766
H	5.0572	4.2791	0.8591	4.9832	4.2926	1.1036	4.9656	4.3252	1.1227
C	3.3262	5.4492	1.3569	3.2186	5.4425	1.5422	3.1842	5.4407	1.5802
H	3.9204	6.2923	1.6982	3.7875	6.2864	1.9210	3.7471	6.2879	1.9618
C	1.9291	5.4915	1.4651	1.8210	5.4626	1.5887	1.7858	5.4557	1.6291
C	1.1522	4.4101	1.0587	1.0660	4.3755	1.1360	1.0517	4.3666	1.1639
H	0.0787	4.4338	1.1979	-0.0120	4.3872	1.2311	-0.0281	4.3645	1.2457
H	1.4474	6.3661	1.8932	1.3099	6.3272	2.0035	1.2680	6.3128	2.0495
C	1.7693	3.2786	0.5035	1.7183	3.2560	0.5970	1.7201	3.2559	0.6180
C	1.2785	1.9648	0.1103	1.2624	1.9511	0.1641	1.2648	1.9617	0.1731
C	0.0000	1.3331	0.0000	0.0000	1.3190	0.0000	0.0000	1.3271	0.0000
C	-1.2785	1.9648	-0.1103	-1.2624	1.9511	-0.1641	-1.2648	1.9617	-0.1731
C	-1.7693	3.2786	-0.5035	-1.7183	3.2560	-0.5970	-1.7201	3.2559	-0.6180
C	-1.1522	4.4101	-1.0587	-1.0660	4.3755	-1.1360	-1.0517	4.3666	-1.1639
H	-0.0787	4.4338	-1.1979	0.0120	4.3872	-1.2311	0.0281	4.3645	-1.2458
C	-1.9291	5.4915	-1.4651	-1.8210	5.4626	-1.5887	-1.7858	5.4557	-1.6291
H	-1.4474	6.3661	-1.8932	-1.3099	6.3272	-2.0035	-1.2680	6.3128	-2.0495
C	-3.3262	5.4492	-1.3569	-3.2186	5.4425	-1.5422	-3.1842	5.4407	-1.5802
H	-3.9204	6.2923	-1.6982	-3.7875	6.2864	-1.9210	-3.7471	6.2879	-1.9618
C	-3.9759	4.3225	-0.8558	-3.9033	4.3250	-1.0491	-3.8853	4.3362	-1.0766
H	-5.0572	4.2791	-0.8591	-4.9832	4.2926	-1.1036	-4.9656	4.3252	-1.1227
C	-3.1957	3.2419	-0.4306	-3.1537	3.2475	-0.5714	-3.1558	3.2515	-0.5938
N	-3.5783	1.9555	-0.0283	-3.5684	1.9788	-0.1352	-3.5891	1.9951	-0.1413
C	-4.8160	1.4246	0.3933	-4.8256	1.4694	0.2018	-4.8601	1.4705	0.1465
C	-5.8862	2.2608	0.7481	-5.9226	2.3193	0.4466	-5.9706	2.3073	0.3534

H	-5.7567	3.3344	0.7403	-5.7845	3.3914	0.4320	-5.8448	3.3812	0.3406
C	-7.1002	1.7202	1.1606	-7.1655	1.7935	0.7683	-7.2231	1.7707	0.6302
H	-7.9162	2.3859	1.4271	-7.9977	2.4655	0.9565	-8.0661	2.4387	0.7838
C	-7.2560	0.3371	1.2460	-7.3339	0.4088	0.8674	-7.3867	0.3882	0.7254
C	-6.1762	-0.4892	0.9438	-6.2379	-0.4289	0.6792	-6.2763	-0.4372	0.5735
H	-6.2863	-1.5651	1.0372	-6.3636	-1.5016	0.7888	-6.3936	-1.5119	0.6779
H	-8.2006	-0.0961	1.5611	-8.3041	-0.0134	1.1113	-8.3614	-0.0424	0.9345
C	-4.9422	0.0228	0.5270	-4.9672	0.0645	0.3634	-5.0004	0.0689	0.2966
C	-3.7913	-0.9487	0.1939	-3.8050	-0.9281	0.1800	-3.8249	-0.9231	0.1649
C	-2.4467	-0.2178	0.1769	-2.4660	-0.2088	0.1327	-2.4971	-0.1824	0.1141
C	-1.2209	-0.8411	0.0992	-1.2263	-0.8661	0.0946	-1.2243	-0.8426	0.0818
H	-1.1662	-1.9262	0.1062	-1.1915	-1.9502	0.1361	-1.1878	-1.9257	0.1397
C	-4.0509	-1.6452	-1.1583	-4.0162	-1.7889	-1.0872	-3.9910	-1.8359	-1.0662
C	-4.1946	-1.0711	-2.4157	-4.1034	-1.3704	-2.4091	-4.0643	-1.4729	-2.4057
H	-4.1287	0.0055	-2.5484	-4.0173	-0.3180	-2.6668	-4.0073	-0.4287	-2.7024
C	-4.4259	-1.9042	-3.5165	-4.3013	-2.3295	-3.4091	-4.2139	-2.4755	-3.3705
H	-4.5391	-1.4688	-4.5055	-4.3720	-2.0170	-4.4473	-4.2740	-2.2065	-4.4215
C	-4.5121	-3.2916	-3.3542	-4.4070	-3.6866	-3.0832	-4.2858	-3.8216	-2.9929
H	-4.6920	-3.9249	-4.2188	-4.5591	-4.4192	-3.8712	-4.4011	-4.5886	-3.7541
C	-4.3691	-3.8701	-2.0907	-4.3169	-4.1085	-1.7544	-4.2098	-4.1889	-1.6472
H	-4.4371	-4.9483	-1.9697	-4.3961	-5.1641	-1.5071	-4.2643	-5.2360	-1.3602
C	-4.1383	-3.0401	-0.9914	-4.1214	-3.1523	-0.7551	-4.0619	-3.1888	-0.6833
C	-3.9508	-3.3572	0.4315	-3.9912	-3.2949	0.7019	-3.9554	-3.2696	0.7806
C	-3.9420	-4.5823	1.1035	-4.0212	-4.4290	1.5176	-3.9662	-4.3703	1.6412
H	-4.0919	-5.5142	0.5644	-4.1605	-5.4186	1.0901	-4.0672	-5.3805	1.2528
C	-3.7352	-4.5919	2.4849	-3.8672	-4.2716	2.8969	-3.8436	-4.1521	3.0155
C	-3.5385	-3.3960	3.1856	-3.6850	-3.0004	3.4545	-3.7110	-2.8543	3.5238
C	-3.5463	-2.1687	2.5134	-3.6547	-1.8640	2.6388	-3.6993	-1.7515	2.6627
H	-3.3912	-1.2415	3.0586	-3.5104	-0.8777	3.0720	-3.5955	-0.7442	3.0577
H	-3.3771	-3.4205	4.2598	-3.5638	-2.8951	4.5291	-3.6151	-2.7022	4.5954
H	-3.7255	-5.5374	3.0206	-3.8877	-5.1449	3.5434	-3.8502	-4.9986	3.6969
C	-3.7549	-2.1565	1.1390	-3.8107	-2.0188	1.2664	-3.8242	-1.9660	1.2951
C	-2.4290	1.1807	0.0621	-2.4347	1.1702	-0.0039	-2.4673	1.1744	-0.0140
C	0.0000	-0.1130	0.0000	0.0000	-0.1579	0.0000	0.0000	-0.1287	0.0000
C	1.2209	-0.8411	-0.0992	1.2263	-0.8661	-0.0946	1.2243	-0.8426	-0.0818
H	1.1662	-1.9262	-0.1062	1.1915	-1.9502	-0.1361	1.1878	-1.9257	-0.1397
C	2.4290	1.1807	-0.0621	2.4347	1.1702	0.0039	2.4673	1.1744	0.0140

C	2.4467	-0.2178	-0.1769	2.4660	-0.2088	-0.1327	2.4971	-0.1824	-0.1141
H	3.7255	-5.5374	-3.0206	3.8877	-5.1449	-3.5434	3.8502	-4.9986	-3.6969
C	3.9420	-4.5823	-1.1035	4.0212	-4.4290	-1.5176	3.9662	-4.3704	-1.6412
H	4.0919	-5.5142	-0.5644	4.1605	-5.4186	-1.0901	4.0672	-5.3805	-1.2528

All calculations are at B3LYP/6-31G(d) in toluene.

VII. References

(1) Ullah, E.; McNulty, J.; Robertson, A. Highly Chemoselective Mono-Suzuki arylation reactions on all three dichlorobenzene isomers and applications development. *Eur. J. Org. Chem.* **2012**, *2012*, 2127-2131.

(2) Chen, C.; Chi, Z.; Chong, K. C.; Batsanov, A. S.; Yang, Z.; Mao, Z.; Yang, Z.; Liu, B. Carbazole isomers induce ultralong organic phosphorescence. *Nat. Mater.* **2021**, *20*, 175-180.

(3) Frisch, M.; Trucks, G.; Schlegel, H.; Scuseria, G.; Robb, M.; Cheeseman, J.; Scalmani, G.; Barone, V.; Petersson, G.; Nakatsuji, H. Gaussian 16, Revision A. 03, Gaussian. *Inc., Wallingford CT* **2016**, *3*.

(4) Sun, H.; Zhong, C.; Bredas, J.-L. Reliable prediction with tuned range-separated functionals of the singlet–triplet gap in organic emitters for thermally activated delayed fluorescence. *J. Chem. Theory Comput.* **2015**, *11*, 3851-3858.

(5) Tomasi, J.; Mennucci, B.; Cammi, R. Quantum mechanical continuum solvation models. *Chem. Rev.* **2005**, *105*, 2999-3094.

(6) Chung, L. W.; Sameera, W.; Ramozzi, R.; Page, A. J.; Hatanaka, M.; Petrova, G. P.; Harris, T. V.; Li, X.; Ke, Z.; Liu, F. The ONIOM method and its applications. *Chem. Rev.* **2015**, *115*, 5678-5796.

(7) Rappé, A. K.; Casewit, C. J.; Colwell, K.; Goddard III, W. A.; Skiff, W. M. UFF, a full periodic table force field for molecular mechanics and molecular dynamics simulations. *J. Am. Chem. Soc.* **1992**, *114*, 10024-10035.

(8) Huang, S.; Zhang, Q.; Shiota, Y.; Nakagawa, T.; Kuwabara, K.; Yoshizawa, K.; Adachi, C. Computational prediction for singlet-and triplet-transition energies of charge-transfer compounds. *J. Chem. Theory Comput.* **2013**, *9*, 3872-3877.

(9) Jiang, Y.; Hu, Z.; Zhou, B.; Zhong, C.; Sun, Z.; Sun, H. Accurate prediction for dynamic hybrid local and charge transfer excited states from optimally tuned range-separated density functionals. *J. Phys. Chem. C* **2019**, *123*, 5616-5625.

(10) Liang, K.; Zheng, C.; Wang, K.; Liu, W.; Guo, Z.; Li, Y.; Zhang, X. Theoretical investigation of the singlet–triplet splittings for carbazole-based thermally activated delayed fluorescence emitters. *Phys. Chem. Chem. Phys.* **2016**, *18*, 26623-26629.

(11) Fang, C.; Oruganti, B.; Durbeej, B. How method-dependent are calculated differences between vertical, adiabatic, and 0–0 excitation energies? *J. Phys. Chem. A* **2014**, *118*, 4157-4171.

(12) Salman, S.; Kim, D.; Coropceanu, V.; Brédas, J.-L. Theoretical investigation of triscarbazole derivatives as host materials for blue electrophosphorescence: effects of topology. *Chem. Mater.* **2011**, *23*, 5223-5230.
(13) Neese, F. Software update: the ORCA program system, version 4.0. *WIREs Comput Mol Sci.* **2018**, *8*, e1327.
(14) Aidas, K.; Angeli, C.; Bak, K.; Bakken, V.; Bast, R.; Boman, L.; Christiansen, O.; Cimiraglia, R.; Coriani, S.; Dahle, P. DALTON, A molecular electronic structure program, release Dalton2016 (2016). *WIREs Comput. Mol. Sci* **2014**, *4*, 269-284.
(15) Byeon, C. C.; McKerns, M. M.; Sun, W.; Nordlund, T. M.; Lawson, C. M.; Gray, G. M. Excited state lifetime and intersystem crossing rate of asymmetric pentaazadentate porphyrin-like metal complexes. *Appl. Phys. Lett.* **2004**, *84*, 5174-5176.
(16) Yoon, S.-J.; Kim, J. H.; Kim, K. S.; Chung, J. W.; Heinrich, B.; Mathevet, F.; Kim, P.; Donnio, B.; Attias, A.-J.; Kim, D.; et al. Mesomorphic Organization and Thermochromic Luminescence of Dicyanodistyrylbenzene-Based Phasmidic Molecular Disks: Uniaxially Aligned Hexagonal Columnar Liquid Crystals at Room Temperature with Enhanced Fluorescence Emission and Semiconductivity. *Adv. Funct. Mater.* **2012**, *22*, 61-69.
(17) Chow, P. C. Y.; Albert-Seifried, S.; Gélinas, S.; Friend, R. H. Nanosecond Intersystem Crossing Times in Fullerene Acceptors: Implications for Organic Photovoltaic Diodes. *Adv. Mater.* **2014**, *26*, 4851-4854.
(18) An, Z.; Zheng, C.; Tao, Y.; Chen, R.; Shi, H.; Chen, T.; Wang, Z.; Li, H.; Deng, R.; Liu, X.; et al. Stabilizing triplet excited states for ultralong organic phosphorescence. *Nat. Mater.* **2015**, *14*, 685-690.
